# Efficacy and safety of clozapine in psychotic disorders—a systematic quantitative meta-review

**DOI:** 10.1038/s41398-021-01613-2

**Published:** 2021-09-22

**Authors:** Elias Wagner, Spyridon Siafis, Piyumi Fernando, Peter Falkai, William G. Honer, Astrid Röh, Dan Siskind, Stefan Leucht, Alkomiet Hasan

**Affiliations:** 1 Department of Psychiatry and Psychotherapy, University Hospital, LMU Munich, Munich, Germany; 2 Department of Psychiatry and Psychotherapy, School of Medicine, Technische Universität München, Klinikum rechts der Isar, Munich, Germany; 3 Department of Psychiatry, Psychotherapy and Psychosomatics of the University Augsburg, Bezirkskrankenhaus Augsburg, Medical Faculty, University of Augsburg, Augsburg, Germany; 4 Department of Psychiatry, The University of British Columbia, Vancouver, Canada; 5 School of Medicine, University of Queensland, Brisbane, Australia; 6 Metro South Addiction and Mental Health Service, Brisbane, Australia

**Keywords:** Scientific community, Schizophrenia

## Abstract

A recent increase in the literature regarding the evidence base for clozapine has made it increasingly difficult for clinicians to judge “best evidence” for clozapine use. As such, we aimed at elucidating the state-of-the-art for clozapine with regard to efficacy, effectiveness, tolerability, and management of clozapine and clozapine-related adverse events in neuropsychiatric disorders. We conducted a systematic PRISMA-conforming quantitative meta-review of available meta-analytic evidence regarding clozapine use. Primary outcome effect sizes were extracted and transformed into relative risk ratios (RR) and standardized mean differences (SMD). The methodological quality of meta-analyses was assessed using the *AMSTAR-2* checklist. Of the 112 meta-analyses included in our review, 61 (54.5%) had an overall high methodological quality according to *AMSTAR-2*. Clozapine appears to have superior effects on positive, negative, and overall symptoms and relapse rates in schizophrenia (treatment-resistant and non-treatment-resistant subpopulations) compared to first-generation antipsychotics (FGAs) and to pooled FGAs/second-generation antipsychotics (SGAs) in treatment-resistant schizophrenia (TRS). Despite an unfavorable metabolic and hematological adverse-event profile compared to other antipsychotics, hospitalization, mortality and all-cause discontinuation (ACD) rates of clozapine surprisingly show a pattern of superiority. Our meta-review outlines the superior overall efficacy of clozapine compared to FGAs and most other SGAs in schizophrenia and suggests beneficial efficacy outcomes in bipolar disorder and Parkinson’s disease psychosis (PDP). More clinical studies and subsequent meta-analyses are needed beyond the application of clozapine in schizophrenia-spectrum disorders and future studies should be directed into multidimensional clozapine side-effect management to foster evidence and to inform future guidelines.

## Introduction

Clozapine—considered the most effective antipsychotic—was introduced in the early 1970s for the treatment of schizophrenia. First, clozapine was believed to have not only superior efficacy but also to have overall better tolerability compared to first-generation antipsychotics (FGA) due to a low risk for extrapyramidal symptoms (EPS). However, in 1975, clozapine was voluntarily withdrawn since 17 out of 2660 (0.7%) patients treated with clozapine in Finland developed agranulocytosis and eight patients subsequently died [1]. In 1988, Kane et al. confirmed clozapine’s safety and superiority vs. chlorpromazine in treatment-resistant schizophrenia (TRS) [2], and subsequently, the Federal Drug Agency (FDA) and other health authorities approved its re-introduction for the indication of TRS with regular hematological monitoring.

Evidence-based treatment guidelines for the management of difficult-to-treat schizophrenia currently recommend clozapine [3–5]. Nevertheless, definitions of TRS, typically involving two failed trials of different non-clozapine antipsychotics, differ significantly across guidelines [6] as do criteria for TRS in clinical trials: if TRS is operationalized at all, it differs in up to 95% of trials [7]. A lack of consensus is also represented in the extent and frequency of mandatory safety monitoring procedures beyond hematological monitoring during clozapine treatment according to the respective national regulations [6]. Further indications or recommendations, when clozapine can be applied in clinical practice, are poorly harmonized: in certain European countries, (e.g. Germany, the Netherlands) clozapine is indicated for the treatment of Parkinson’s disease psychosis (PDP), whereas in the US it was given a Level B recommendation by the American Academy of Neurology (AAN) for this indication. Furthermore, the FDA approved clozapine as the first agent indicated for suicidality in people with schizophrenia and schizoaffective disorder. Furthermore, the American Psychiatric Association (APA) recommends (1B) that patients with TRS be treated with clozapine and recommends (1B) patients with schizophrenia be treated with clozapine if the risk for suicide attempts or suicide remains substantial despite other treatments and suggests (2C) that patients with schizophrenia be treated with clozapine if the risk for aggressive behavior remains substantial despite other treatments [8]. Of note, clozapine is recommended in some clinical guidelines for treatment-refractory bipolar disorder [9] with an uncertain body of evidence suggesting beneficial effects on e.g. mania, depression, rapid cycling and psychotic symptoms [10].

Even though clozapine is considered one of the most effective medications and is listed in the WHO Model List of Essential Medicines [11], there is frequently a delay in clozapine initiation, leading to poorer mental health and functional outcomes [10, 12], preceded by attempts of polypharmacy treatment without evidence for effectiveness [13].

The scientific literature regarding clozapine is vastly increasing and evidence-based psychiatry might help clinicians to judge the best evidence and decision-makers and clinicians are overstrained by the number of individual studies, reviews and meta-analyses [14].

Thus, with our quantitative meta-review of meta-analyses we aimed at elucidating the state-of-the-art of efficacy, effectiveness, tolerability and management of clozapine and clozapine-related adverse-events in order to synthesize evidence, provide orientation for decision-makers and clinicians and identify treatment gaps for future research.

## Methods

### Information sources and search

**Fig. 1:**
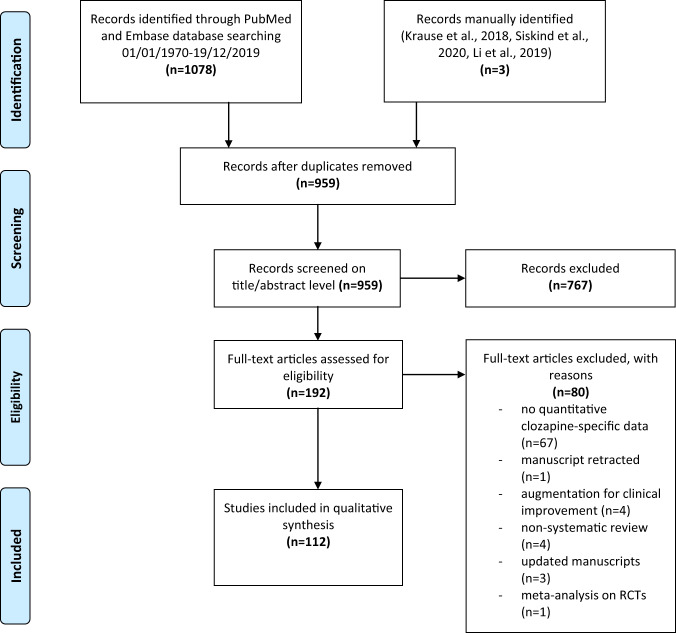
Flowchart for literature search and study selection process [19]. Study selection flow diagram of meta-analyses providing quantitative data. Three meta-analyses were manually identified.

This meta-review was pre-registered on PROSPERO (*CRD42020164135*). Following the structure of the International Statistical Classification of Diseases and Related Health Problems 10th Revision (ICD-10 WHO Version, 2015), we searched the PubMed/MEDLINE database and the EMBASE databases using the following search terms with limitation to systematic reviews and/or meta-analyses: “clozapine” OR “leponex” OR “clozaril”. The literature searches and selection were independently performed by EW and PiyF and validated by AH. The titles and the abstracts of each citation were screened manually, and the full text of each potentially relevant citation was retrieved for detailed review. Pharmacological or non-pharmacological clozapine augmentation/combination strategies with the purpose of clinical improvement were excluded a priori since evidence in this field was already meta-reviewed by members of our group [15]. Furthermore, studies focusing on genetics and/or pharmacogenetics, brain-imaging studies, cost-effectiveness studies, and animal studies were excluded. Three publications [16–18] were added by hand since two were published after the search period [16, 18] and one included sub-analyses for a new domain [17] (see Fig. 1).

### Eligibility criteria

The inclusion criteria were all meta-analyses published in English between January 1, 1970, and December 19, 2019 (PubMed) and 1970–2019 (EMBASE) with quantitative data of people treated with clozapine alone or clozapine vs any control (clozapine, placebo, or non-clozapine antipsychotics). The major exclusion criteria were the absence of clozapine-specific meta-analytic data. We extracted clozapine-specific meta-analytic data on effectiveness, efficacy, and tolerability of clozapine, management of clozapine, and clozapine-related adverse events. The applied search strategy according to The Preferred Reporting Items for Systematic Reviews and Meta-Analyses (PRISMA) guidelines [19] is shown in Fig. 1.

### Data collection process

After full-text review, one researcher (EW) extracted quantitative data from pairwise meta-analyses with validation by a second researcher (PiyF). Network meta-analytic data was extracted if pairwise analyses were presented. If standardized mean difference (SMD), mean difference (MD), risk difference (RD) > 0 demonstrated a beneficial outcome for clozapine (e.g. more response or less adverse events) then the direction ‘clozapine’, was extracted, however, if <0 then the direction “control” was extracted. If RR, odds ratio (OR), hazard ratio (HR) > 1 meant a beneficial outcome for clozapine (e.g. more response or less adverse events/dropouts) then the direction ‘clozapine’, otherwise ‘control’, was extracted. Furthermore, we grouped outcomes into short-term (up to 12 weeks), medium-term (13–26 weeks), and long-term (over 26 weeks).

### Data transformation

The data transformation process was conducted by two authors (EW and SS) with validation by a third author (SL) using R statistical software version 4.0.3 [20] and the package tidyverse version 1.1.3 [21] OR and RD were transformed to RR [22] while HR and incidence rate ratio (IRR) was used as RR. MD was transformed into SMD [23], and in case the total number of participants in the control and experimental group were not given, equal groups were assumed. A beneficial outcome for the experimental intervention was represented with SMD > 0 or OR > 1, and minus or inverse transformations were applied whenever the opposite direction was reported. Due to limited data, adverse events of clozapine add-on strategies were not able to be included in the analyses.

### Endpoints

Endpoints were defined as (1) efficacy of clozapine (SMD and RR), (2) tolerability/adverse events of clozapine (SMD and RR), and (3) efficacy of add-on strategies to improve clozapine-related adverse events (SMD and RR).

### Methodological quality assessment of included meta-analyses

The *Assessing the Methodological Quality of Systematic Reviews 2 (AMSTAR-2)* checklist [24] was used independently by two reviewers (EW, PiyF). Disagreements were solved by consensus with a third reviewer (AH). Then, meta-analyses were categorized into different domains according to their objectives, taking into consideration participant characteristics, comparisons, and outcomes. In case of an overlap of two domains within one meta-analysis, categorization was performed with a primary focus on population characteristics (e.g. first-episode schizophrenia) before outcomes (e.g. metabolic outcomes) (see Table 1).

**Table 1:** Description of included meta-analyses.

Author (year)	Inclusion criteria for study type	Specific domain(s) of interest	CLZ-specific MA	Population	Studies included for MA, total (*n*)	CLZ studies analyzed	Statistical model	Measure	N total	N CLZ + controls	Duration of studies (total)	Primary outcome
*Autonomic nervous system dysfunction*												
Alvares (2014)	OBS	Autonomic nervous system dysfunction and psychotropic medication	No	Psychiatric disorders	173	3	Random- effects	Hedge’s *g*	13,527	63	NS	Heart rate variability
*Bipolar disorder (BD)*												
Delgado (2020)	RCTs, OBS	CLZ for treatment of mania in BD	Yes	CLZ users with BD	3 RCTS	3	Random effects	MD	202	100	4–6 w	mainly BRMS
*Cardiological complications*												
Salvo (2016)	OBS	Sudden cardiac/unexpected death (SCD/SUD)	No	AP-users vs. non-AP controls	6	1	Random effects	OR	1729	19	NS	SCD/SUD
Siskind (2020)	RCTs, OBS	Myocarditis/cardiomyopathy	Yes	CLZ users	28	28	Random effects	Incidence rate ratio	258,961	258,961	24 m (median)	Event rates of myocarditis
Lally (2016)	RCTs	Pharmacological interventions for CLZ-induced sinustachycardia	Yes	CLZ users	0	0	NA	RR	0	0	NA	Change in heart rate
*Children and adolescents*												
Arango (2019)	RCTs	Efficacy and tolerability of Lurasidone vs. other SGAs	No	Sz-spectrum	13	1	Fixed	SMD, OR	NS	25	6–12 w	CGI, PANSS, weight gain
Cohen (2012)	RCTs, OBS	Adverse effects of SGAs	No	psychiatric disorders	41 controlled trials	5	Bayesian MA	OR, MD	4015	79	3–12 w	Metabolic parameters
Krause (2018)	RCTs	Efficacy and tolerability of FGAs and SGAs	No	Sz-spectrum	28	2	Random effects	SMD	3003	22	6 weeks (median)	Overall change in symptoms
Kumar (2013)	RCTs	Efficacy of SGAs	No	Sz-spectrum	13	1	Random effects	RR	1112	21	3 w–6m	Global state, clinical response
Sarkar (2013)	RCTs	Efficacy and tolerability of FGAs and SGAs	No	Sz-spectrum	15	3	Random effects	Cohen’s *d*	NS	85	4–12 w	PANSS, BPRS, CGI
Pringsheim (2011)	RCTs	Metabolic and neurological complications of SGAs	no	psychiatric disorders	35	3	Random effects	OR, MD	NS	85	32 trials <12 w	Metabolic parameters
*Childhood-onset Sz*												
Kennedy (2007)	RCTs	Efficacy and tolerability of FGAs and SGAs	No	Sz-spectrum	6	1	Random effects	RR	NS	21	6–12 w	Overall change in symptoms
*Cognition*												
Nielsen (2015)	RCTs	Efficacy of SGAs and FGAs on cognitive domains	No	Sz-spectrum	37	9 (+3 CLZ + X studies)	Random effects regression	Cohen’s *d*	3526	238	23.6 w (mean)	Cognitive domains
Thornton (2006)	RCTs, OBS	Efficacy of SGAs and FGAs on long-term memory	No	Sz-spectrum	17	5	Fixed effects	Cohen’s *d*	939	188	3–52 w	Change in long-term memory
Woodward (2005)	RCTs	Efficacy of SGAs and FGAs on cognitive domains	No	Sz-spectrum	44	3 in 1^st^, 17 in 2^nd^ analysis	Fixed effects	Hedge’s *g*	NS	73 in 1st, 344 in 2nd	31 w (mean)	Change in cognitive domains
*Comorbid depression*												
Furtado (2014)	RCTs	Efficacy of SGAs vs. FGAs/SGAs for Sz + depression	No	Sz-spectrum	3	1	Random-effects	WMD, RR, NNT	310	29	≤12 w	Overall outcome
*Comorbid substance abuse*												
Krause (2018)	RCTs	Efficacy and tolerability of APs in Sz + substance abuse	No	Sz-spectrum	19	4	Random-effects	OR, SMD	1742	97	4–72 w	Reduction of substance use
Temmingh (2018)	RCTs	RIS vs other APs in severe mental illness + substance abuse	No	Psychiatric disorders	8	2	Random-effects	MD, RR	1073	50	4–52 w	Overall outcome
*Constipation and gastrointestinal hypomotility*												
Every-Palmer (2017)	RCTs	Pharmacological treatment for AP-related constipation	No	Psychiatric disorders	2 Chinese trials in qualitative synthesis (quality unclear)	2	Fixed-effects	RR	480	306	1–14 days	Change in constipation
Shirazi (2016)	RCTs, OBS	Prevalence and predictors of CLZ-associated Constipation	Yes	Sz-spectrum	32	11	Random effects	OR	2013	2013	NS	Constipation rate
*Discontinuation of treatment in schizophrenia*												
Beasley (2007)	RCTs	All-cause treatment discontinuation	No	Sz-spectrum	16	3	Cox regression	Mean HR	NS	487	18–104 w	Rate of treatment discontinuation
Masuda (2019)	OBS	Hospitalization and all-cause treatment discontinuation, CLZ vs. other oral SGAs	Yes	Sz-spectrum	63	63	Random effects	Hedge’s *g*, RR	109,341	109,341	19.1 m (mean)	Hospitalization and ACD rate
Soares-Weiser (2012)	RCTs, OBS	Time to all-cause treatment discontinuation	No	Sz-spectrum	60 RCTs, 27 OBS	8 RCTs, 9 OBS	Random effects	HR, RR	33,360 (RCTs) +202,591 (OBS)	1754 (RCTs) + 13,911 (OBS)	3–24 m (RCTs), 3 m- 6 yrs (OBS)	Time-to-discontinuation
*Dose-response relationship and disposition of clozapine*												
Leucht (2014)	RCTs	Minimum effective dose of SGAs	No	Sz-spectrum	73	1	Fixed-effects	SMD/Hedge’s *g*	NS	NS	6.9 w (mean)	PANSS, BPRS
Subramanian (2017)	RCTs	Clozapine dose for Sz	Yes	Sz-spectrum	5	5	Random-effects	RR	452	452	6–48 w	Overall outcome
Tsuda (2014)	OBS	Effects of smoking on disposition of OLA and CLZ	Yes	Psychiatric disorders	7 OLA, 4 CLZ	4	Random-effects	WMD	1094 OLA + 196 CLZ	196	NA	C/D ratio
*Efficacy and tolerability for non-first-episode and non-treatment-resistant schizophrenia-spectrum disorders*												
Asenjo Lobos (2014)	RCTs	Efficacy and adverse events of CLZ vs. other oral SGAs	Yes	Sz-spectrum	27	27	Random-effects	RR, MD, NNT	3099	3099	12 w (20), 12–26 w (5), >26 w (2)	Overall outcome
Asmal (2013)	RCTs	Efficacy and tolerability of QUE vs other oral SGAs	No	Sz-spectrum	35	5	Random-effects	RR, MD	1486	334	2–12 w (26), 6 medium, 2 long-term	Overall outcome
Bai (2016)	RCTs	Comparative efficacy and tolerability of 8 SGAs	No	acute Sz (Chinese)	60	8	Random-effects	OR	6418	NS	9 w (mean)	Overall outcome
Davis (2003)	RCTs	Efficacy of SGAs vs. FGAs, SGAs vs. SGA	No	Sz-spectrum	124	31	Fixed-effects	Hedge’s g	NS	NS	NS	PANSS, BPRS
Duggan (2005)^a^	RCTs	Efficacy and tolerability OLA vs. PLC, FGAs, SGAs	No	Sz-spectrum	55	8	Random effects	RR, MD	>10,000	NS	<3 m (31), 9–12 m (23), >1 yr (2)	Overall outcome
Essali (2009)	RCTs	Efficacy and tolerability CLZ vs. FGAs (different Sz populations)	Yes	Sz-spectrum	52	52	Fixed-effects	RR, SMD	4746	4746	>26 w (7), max. 12 w (44), 1 trial both short and long term	Overall outcome
Geddes (2000)	RCTs	Efficacy and tolerability of SGAs vs. FGAs	No	Sz-spectrum	52	12 trials on efficacy, 20 on tolerability	Fixed-effects	OR	12,649	NS	6 w (median)	Overall outcome
Glick (2011)	RCTs	Comparative Mid- and Long-Term Efficacy and Tolerability of SGAs	No	Sz-spectrum	NS	NS	Personalized, data-driven approach	RR, HR	NS	NS	NS	ACD, relapse, drop-out, side-effects
Hartling (2012)	RCTs, OBS	Comparative efficacy and tolerability of FGAs vs. SGAs	No	Sz-spectrum	114	max. 4	Random-effects	MD	Max. 118,522	Max. 607	8 w (median)	Overall outcome
Khanna (2014)	RCTs	Comparative efficacy and tolerability of ARI vs. SGAs	No	Sz-spectrum	174	29	Random-effects	RR, MD	17,244	2132	Mostly short-term 3–8 w	Overall outcome
Kishi (2017)	RCTs	Efficacy and tolerability of SGAs, HAL and PLC	No	Sz-spectrum, Japanese	18	NS	Bayesian network	OR	3446	47	8.3 w (mean)	Response rate, ACD
Kishimoto (2019)	RCTs	Long-term effectiveness and tolerability of SGAs vs. SGAs	No	Sz-spectrum	59	8 in total (CLZ in subanalyses)	Random- effects	RR, SMD	45,787	30–1202 (only subanalyses)	47.4 w (mean)	Overall outcomes
Klemp (2011)	RCTs	Efficacy and tolerability of 4 SGAs	No	Sz-spectrum	30	5	Joint model	RR	7743	1108	2–12 w (21), 12–52 w (9)	Response ratio
Komossa (2013)	RCTs	Efficacy and tolerability of OLA vs. other SGAs	No	Sz-spectrum	50	12	Random-effects	RR, WMD	9476	NS (only subanalyses)	Mostly short-term, only 9 studies >26 w	Overall outcome
Komossa (2014)	RCTs	Efficacy and tolerability of QUE vs. other SGAs	No	Sz-spectrum	21	5	Random-effects	RR, WMD	4101	NS (only subanalyses)	2–12 w (15), medium-term (3), long-term (2)	Overall outcome
Komossa (2010)	RCTs	Efficacy and tolerability of ZOT vs. other SGAs	No	Sz-spectrum	2	2	Random-effects	RR, WMD	109	109	Short term (2)	Overall outcome
Komossa (2009)	RCTs	Efficacy and tolerability of ZIP vs. other SGAs	No	Sz-spectrum	9	1	Random-effects	RR, WMD	3361	146	6–12 w (4), 18–26 w (3), 28-78 w (2)	Overall outcome
Komossa (2011)	RCTs	Efficacy and tolerability of RIS vs. other SGAs	No	Sz-spectrum	45	11	Random-effects	RR, WMD	7760	NS	< 12 w (31), 13–26 w (6), >26 w (8)	Overall outcome
Leucht (2009a)	RCTs	Efficacy of SGAs vs. SGAs Head-to-Head	No	Sz-spectrum	78	28	Random-effects, fixed-effects	WMD, Hedges’ *g*, RR	13,558	Max. 619 (subanalyses)	NS	PANSS
Leucht (2009b)	RCTs	Efficacy of SGAs vs. PLC	No	Sz-spectrum	38	1	Random-effects	Hedges’ *g*, SMD	7323	22	2-50 w	Overall symptoms
Leucht (2009c)	RCTs	Efficacy and tolerability of SGAs vs. FGAs	No	Sz-spectrum	150	23	Random-effects	Hedges’ *g*, SMD	21,533	1997	≤12 w (121), up to 6 m (12), >6 m (12)	Overall symptoms
Leucht (2013)	RCTs	Comparative efficacy and tolerability of 15 AP drugs	No	Sz-spectrum	212	22	Bayesian framework	OR, SMD	43,049	NS	4–52 w	Overall symptoms
Okhuijsen-Pfeifer (2020)	OBS	Demographic and clinical CLZ-response predictors	Yes	Sz-spectrum	34	34	Random-effects	Hedges’ g	9386	9386	NS	Response predictors
Samara (2014)	RCTs	Efficacy of CPZ vs. FGAs/SGAs	No	Sz-spectrum	128	10	Random-effects	RR, SMD	10,667	778	3–52 w	Response to treatment
Sherwood (2012)	RCTs	Response profile to CLZ	Yes	Sz	19	19	Regression analyses	Paired *t*-test, Cohen’s *d*	1745	1745	4–18 w	Response profile
Subramanian (2012)	RCTs	Efficacy of ZOT vs. SGAs	No	Sz-spectrum	3	2	Random-effects	MD, RR	289	239	4 w, 6 w, 12 w	PANSS-EC
Szegedi (2012)	RCTs	Efficacy of Asenapine vs. PLC, SGAs	No	acute Sz	58	1	Random effects	OR, Hedges’ *g*	NS	NS	NS	PANSS
Tuunainen (2002)	RCTs	Efficacy and tolerability of SGAs vs. CLZ	Yes	Sz-spectrum	8	8	Fixed-effects	SMD, RR	795	795	7 short-term, 18 w (1)	Overall outcome
Tuunainen (2000)	RCTs	Efficacy and tolerability of CLZ vs. SGAs	Yes	Sz-spectrum	8	8	Random-effects	SMD, RR	795	795	7 short-term, 18 w (1)	Overall outcome
Wahlbeck (1999)^b^	RCTs	Efficacy and tolerability of CLZ vs. FGAs	Yes	Sz-spectrum	31	31	Random-effects	OR, SMD	2589	2589	Mostly <13 w (26)	Overall outcome
*Elderly patients with schizophrenia*												
Krause (2018)	RCTs	Efficacy and tolerability of SGAs and FGAs	No	Sz-spectrum	18	3	Pairwise random-effects	SMD, OR	1225	54	10 w (median)	PANSS
*Extrapyramidal symptoms (EPS), Tardive dyskinesia (TD)*												
Bergman (2018)	RCTs	Antipsychotic reduction and/or cessation in TD	No	Sz-spectrum	13	1	Random-effects, fixed-effects	RR, MD	711	39	<6 m (8), >6 m (5)	Reduction in TD
Carbon (2018)	RCTs	TD risk with FGAs and SGAs	No	Sz-spectrum	32	6	Random-effects	RR, annualized RR	10,706	348	1 yr (median)	TD risk
Leucht (2003)	RCTs	SGAs vs. FGAs in terms of risk of EPS	No	Sz-spectrum	31	11	Random-effects, fixed-effects	RD	2320	758	6 w (median)	Number of patients with at least one EPS
Mentzel (2018)	RCTs, OBS	CLZ-Monotherapy as treatment for TD	Yes	Sz-spectrum	17	17	Random-effects	MD	1217	1217	1.5 m–5 yrs	Change in TD rating scale score
Rummel-Kluge (2010)	RCTs	SGAs vs. SGAs in terms of risk for EPS	No	Sz-spectrum	54	15	Random-effects, fixed-effects	RR	NS	NS	NS	Use of antiParkinson medication at least once
*First-episode schizophrenia-spectrum (FES)*												
Tek (2015)	RCTs	weight gain in FGAs and SGAs vs. PLC	No	Sz-spectrum	28	2	Random-effects, fixed-effects	MD	4139	NS	NS	Change in weight
Zhang (2013)	RCTs	Efficacy and tolerability of SGAs vs. FGAs	No	acute Sz-spectrum	13	2	Random-effects	Hedges’ *g*, RR	2509	NS	32.1 w (mean)	Overall outcome
*Hospitalization rate in schizophrenia*												
Land (2017)	RCTs, OBS	Impact of CLZ on hospital use	Yes	Sz-spectrum	37 (3 RCTs, 34 OBS)	37	Random-effects	MD, RR	12,631 + 35,337 controls	12,631 + 35,337 controls	Up to 364 w	Hospital use for any reason
*Hypersalivation/Sialorrhea*												
Chen (2019)	RCTs	Treatment strategies for CLZ-induced hypersalivation	Yes	Sz-spectrum	19	19	Random-effects	RR, NNT	NS	NS	10 days–6w	Change in sialorrhea
Syed (2012)	RCTs	Treatment of CLZ-induced hypersalivation	Yes	Sz-spectrum	15	15	Random-effects	RR, NNT, MD	NS	NS	All <3 m	Change in sialorrhea
*Schizophrenia with intellectual disabilities*												
Ayub (2015)	RCTs	CLZ for psychotic disorders + intellectual disabilities	Yes	Sz-spectrum	0	0	NA	NA	0	0	NA	Overall outcome
*Metabolic complications*												
Bak (2014)	RCTs	Weight gain of FGAs and SGAs	No	Sz-spectrum	307	Dependent on subanalysis	Random-effects	Pooled absolute changes	NS	NS	Short-, mid- and long-term	Change in metabolic parameters
Bartoli (2015a)	OBS	SGAs and adiponectin levels	No	Sz-spectrum	8	Dependent on subanalysis	Random- effects	SMD	1515	NS	NS	Adiponectin levels
Bartoli (2015b)	OBS	SGAs and adiponectin levels	No	Sz-spectrum	18	2	Random-effects	SMD	2735	NS	NS	Adiponectin levels
Buhagiar (2019)	OBS	FGAs vs. SGAs and lipid abnormalities	No	psychiatric disorders	18	2	Random-effects	OR, SMD	NS	3415	12 m (median)	Lipid metabolism
Correll (2016)	RCTs	Efficacy and safety of TOP- Cotreatment for Body Weight	No	Sz-spectrum	8	4	Random-effects	SMD, WMD, RR	439	172 for clozapine augmentation subanalyses	13.6 w (mean)	Overall outcomes
Mitchell (2011)	OBS	Metabolic syndrome and abnormalities	No	Sz-spectrum	126	13	Random-effects	Proportion with CI	25,692	673	NS	MetS rates in Sz
Pillinger (2019)	RCTs	Effects of 18 APs on metabolic outcomes + psychopathology	No	Sz-spectrum	100	5	Random-effects network	MD, SMD	25,952	NS	6 w (median)	Change in metabolic outcomes
Potvin (2015)	OBS	AP-induced changes in blood levels of leptin	No	Sz-spectrum	28	4	Random-effects	Hedges’ *g*	NS	NS	NS	Leptin blood level change
Rummel-Kluge (2010)	RCTs	Metabolic side-effects of SGAs	No	Sz-spectrum	48	11	Random-effects, fixed-effects	SMD	NS	NS	NS	Weight change
Siskind (2016)	RCTs	Metformin vs. PLC for CLZ-associated obesity	Yes	Sz-spectrum	8	8	Random-effects	MD, RR	478	478	NS	Weight loss, BMI
Siskind (2018)	RCTs	GLP-1 Receptor agonizts for AP-associated cardiometabolic risk factors	No	Sz-spectrum	4	3	Random-effects	SMD	164	113	16.2 w (mean)	Change in body weight
Smith (2008)	RCTs, OBS	FGAs vs. SGAs and risk for diabetes	No	Sz-spectrum	11	7	Random-effects	RR	NS	NS	12 m (median) without CS	Risk for diabetes with FGA vs. SGA
Srisurapanont (2015)	RCTs	Efficacy and safety of CLZ + ARI for cardiometabolic risk reduction	Yes	Sz-spectrum	4	4	Random-effects	RR, SMD	347	347	8–24 w	Overall outcomes
Vancampfort (2015)	OBS	Prevalence of MetS	No	Psychiatric disorders	198	30	Random-effects	RR	52,678	NS	NS	Prevalence of MetS
Zhang (2017)	RCTs	Metabolic side-effects on glucose of 12 APs	No	Sz-spectrum	47	6	Network	MD	NS	NS	NS	Changes in blood glucose levels
Zheng (2016)	RCTs	Efficacy and safety of adjunctive TOP for weight reduction	No	Sz-spectrum	16	4	Random-effects	SMD, WMD, RR	934	213	11.8 w (mean)	Overall outcomes
Zimbron (2016)	RCTs	Treatment strategies for CLZ-induced obesity and MetS	Yes	Sz-spectrum	15	15	Random-effects	MD	NS	NS	All ≤6 m	Change in metabolic outcomes
*Mortality in schizophrenia*												
Vermeulen (2019)	OBS, RCT	CLZ and Long-Term Mortality Risk	Yes	Sz-spectrum	24 (1 RCT, 23 OBS)	24	Random-effects	RR	NA, 217,691 patient years	NA, 217691 patient years	5.4 yrs (median)	Mortality rate
*Multi-episode schizophrenia (MES)*												
Huhn (2019)	RCTs	Comparative efficacy and tolerability of 32 oral APs for acute treatment	No	Acute MES	402	31	Random-effects	SMD	53,463	NS	NS	PANSS, BPRS
*Negative symptoms in schizophrenia*												
Krause (2018)	RCTs	APs for predominant negative symptoms	No	Sz-spectrum	21	1	Pairwise random-effects	SMD	3451	21	12 w (median)	Negative symptoms
*Neutropenia*												
Li (2019)	OBS	Prevalence of agranulocytosis in CLZ users	Yes	Sz-spectrum	36	36	Random- effects	Prevalence rate	260,948	260,948	Months-years	Rate of agranulocytosis
Myles (2018	OBS	Epidemiology of CLZ-associated neutropenia	Yes	Sz-spectrum	108	108	Random-effects	Estimated event rates	119,592	119,592	12 m (median)	Rates of neutropenia
Myles (2019)	RCTs, OBS	Association between CLZ and other APs and neutropenia risk	Yes	Sz-spectrum	20 (17 RCTs, 3 OBS)	20	Random-effects	RR	1260	1260	3.5 m (median)	Rates of neutropenia
*Parkinson’s disease psychosis (PDP) and drug-induced psychosis in PD*												
Frieling (2007)	RCTs	Efficacy of treatment strategies for DIP in PD	No	PD + DIP	7	3	Fixed-effects	WMD, SMD, RR	419	NS	NS	Change in psychotic symptoms
Iketani (2017)	RCTs	Comparative utility of SGAs for treatment of PDP	No	PDP	10	4	Random-effects	MD	NS	64	4–56 w	BPRS, UPDRSM
Jethwa (2015)	RCTs	Efficacy of APs in treatment of PDP	No	PDP	9	2	Random-effects	MD	NS	35	4–12 w	BPRS, UPDRSM
Zhang (2019)	RCTs	Efficacy of SGAs for PDP	No	PDP	13	2	Fixed-effects, random-effects	WMD	1142	298	4–12 w	Various psychosis outcome scales
*Pneumonia*												
Dzahini (2018)	OBS	FGAs and SGAs and risk for pneumonia	No	Psychiatric disorders	14	2	Random-effects, fixed-effects	RR	NS	NS	NS	Risk for pneumonia
*Psychosocial function in schizophrenia*												
Olagunju (2018)	RCTs	CLZ and psychosocial function	Yes	Sz-spectrum	9	9	Random-effects	SMD	1279	1279	10–104 w	Change in psychosocial function
*Relapse prevention in schizophrenia*												
Kishimoto (2013)	RCTs	Relapse prevention of SGAs vs. FGAs	No	Sz-spectrum	23	4	Random-effects	RR, NNT	4504	355	61.9 w (mean)	Study-defined relapse
Leucht (2003)	RCTs	Relapse prevention of SGAs	No	Sz-spectrum	17	3	Random-effects, fixed-effects	RD	3015	NS	NS	Relapse rate
*Second-line treatment in schizophrenia*												
Cheine (1998)	RCTs	Pharmacological treatment of Sz resistant to first-line treatment	No	Sz-spectrum	21	4	Random-effects	OR, NNT	NS	772	NS	Psychotic symptom outcome
Okhuijsen-Pfeifer (2018)	RCTs, OBS	CLZ as first- or second-line treatment in Sz	Yes	Sz-spectrum	15	15	Random-effects	Hedges’ *g*	1114	1114	NS	Treatment response
*Sexual dysfunction (SD)*												
Serretti (2011)	RCTs, OBS	Association of sexual dysfunction and intake of APs	no	Psychiatric disorders	34	NS	Random-effects	OR	NS	NS	4 w–12 m	Rate of total sexual dysfunction related to AP
*Suicidality and hostility/aggression vs. others in schizophrenia*												
Faay (2018)	RCTs, OBS	Efficacy of FGAs and SGAs on hostility	No	Sz-spectrum	18	5	Random-effects	Hedges’g	6799	290 (only clozapine) + 247 HAL	4–78 w	Change in hostility scores
Hennen (2004)	RCTs, OBS	Efficacy of CLZ on suicidal risk	Yes	Sz-spectrum	6	6	Random-effects	RR	240,564	240564	NA, 104,796 yrs of CLZ exposure vs. 44,7281 other AP exposure	Risk of suicidal behavior, suicide attempts
Khushu (2016)	RCTs	Efficacy of HAL vs. other APs for long-term aggression	No	Sz-spectrum	1	1	Random-effects	RR	83	83	12 w	Change in aggression score
*Treatment-resistant schizophrenia-spectrum (TRS)*												
Chakos (2001)	RCTs	Efficacy of SGAs	No	TRS	12	7	ANCOVA, weighted least squares, Cohen’s *d*, C–M–H method for categorical data	Mean, Cohen’s *d*	1916	>1000 dependet on subanalysis	NS	Overall outcome
Mizuno (2019)	RCTs	Efficacy of APs for Sz with or without TRS	No	Sz-spectrum	10 TRS studies, 29 non-TRS	12 and 33 treatment arms respectively	Random-effects	Hedges’ *g*	822 and 2566, respectively	Dependent on subanalysis	11 and 8 w, respectively (median)	Total symptoms
Moncrieff (2003)	RCTs	Efficacy of CLZ vs. FGAs	Yes	TRS	10	10	Fixed-effects, random-effects	SMD	NS	NS	NS	Change in psychotic symptom scores
Samara (2016)	RCTs	Efficacy and tolerability of APs	No	TRS	40	20	Random-effects, Bayesian setting	OR, SMD	5172	NS	11w (median)	Overall change in symptoms
Siskind (2016)	RCTs	Efficacy of CLZ vs. FGAs and SGAs	Yes	TRS	21	21	Random-effects	SMD, RR	2364	2364	6–52 w	Change in symptoms, response rates
Siskind (2017)^c^	RCTs	CLZ response rates among people with TRS	Yes	TRS	21	21	Arcsine transformation for binomial variables	Proportion or response	2364	2364	6–-52 w	Response rates
Souza (2013)	RCTs	Efficacy of OLA vs. CLZ	Yes	TRS	7	7	Fixed-effects	RR, SMD	648	648	NS	Response rate

*d* days, m months, *w* weeks, *yrs* years, *ACD* all-cause discontinuation, ANCOVA analysis of covariance, *AP* antipsychotic, *ARI* Aripiprazole, *BRMS* Bech–Rafaelsen Mania Scale, *BMI* body mass-index, *BPRS* Brief Psychiatric Rating Scale, *CGI* Clinical Global Impression Severity Scale, *CI* confidence interval, *CLZ* clozapine, *C/D* ratio concentration to dose ratio, *C–O–H* Cochrane–Mantel–Haenszel, *CPZ* chlorpromazine, *DIP* drug-induced psychosis, *FGA* first-generation antipsychotic, *HAL* haloperidol, *HR* hazard ratio, *MD* mean difference, *MES* multi-episode schizophrenia, MetS, *NA* not applicable, *NNT* number needed to treat, *NS* not specified, *OBS* observational study, *OLA* olanzapine, *PANSS* Positive and Negative Syndrome Scale, *PD* Parkinson disease, *PDP* Parkinson disease psychosis, *PLC* placebo, *RCT* randomized controlled trial, *RD* risk difference, *RR* relative risk, *SCD* sudden cardiac death, *SD* standard deviation, *SGA* second-generation antipsychotic, *SMD* standardized mean difference, *SUD* sudden unexpected death, *SZ* schizophrenia, *TD* tardive dyskinesia, *TOP* topiramate, *TRS* treatment-resistant schizophrenia, *UPDRS-III* Unified Parkinson’s Disease Rating Scale parts III, *UPDRSM* Unified Parkinson’s Disease Rating Scale–Motor Subscale, *WMD* weighted mean difference.

^a^Meta-analysis Duggan et al., 2005 was published as third update (after 2000 and 2003) in *Cochrane Database of systematic reviews* and thus only this version (2005) was reviewed.

^b^Meta-analysis “Evidence of clozapine’s effectiveness in Schizophrenia: A Systematic Review and Meta-Analysis of Randomized Trials” Am J Psychiatry, 1999;156:990–999 was published as Cochrane review in The Cochrane Library 1999, Issue 4. Only the publication in Am J Psychiatry was reviewed since clozapine-related findings were identical.

^c^Used data from Siskind et al., 2016.

## Results

1078 records were identified and the publications were added manually [16–18]. After the removal of duplicates, 959 records remained. A total of 767 records were excluded on the title/abstract level. The remaining 192 publications were retrieved as full texts and were further assessed for eligibility. From these, 112 records were included in this meta-review. 80 records were excluded as they met at least one of the exclusion criteria on full text-level (see Fig. 1). Since no evidence is considered an important finding according to the Cochrane Handbook [25], two clozapine-specific Cochrane Database reviews/meta-analyses that yielded no quantitative data due to a lack of relevant studies [26, 27], were included in our umbrella review.

### Study characteristics/AMSTAR ratings

From the 112 included meta-analyses [10, 16–18, 26–131] a majority reported data on clozapine as subgroup or sensitivity analysis, whereas 34 exclusively targeted populations of clozapine users (see Table 1). According to *AMSTAR-2*, 61 (54.5%) meta-analyses were rated as high-quality. A description of the results of each meta-analysis along with their overall quality is presented in the Supplementary Tables (see Supplementary Tables S1 and S2, https://github.com/sksiafis/clozapine_meta_review).

### Endpoints

#### Efficacy of clozapine (SMD and RR)

##### Positive symptoms in schizophrenia

**Fig. 2:**
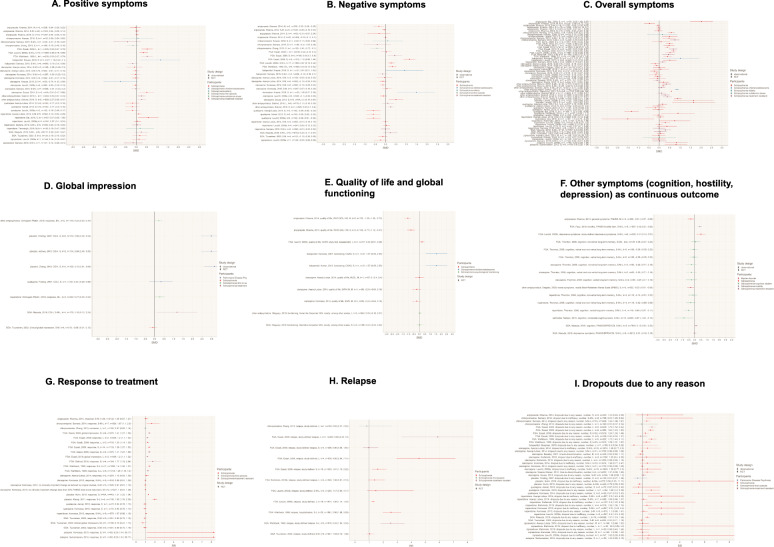
Quantitative meta-review of clozapine-specific meta-analytic data: Efficacy. **A** Positive symptoms. FGA first-generation antipsychotic, *k* number of studies, L long-term, M medium-term, *n* number of participants, RCT randomized controlled trial, S short-term, SGA second-generation antipsychotic. Abbreviated study descriptions: Leucht et al., 2009a [76], Leucht et al., 2009b [76], Leucht et al., 2009c [79]. For continuous outcomes, SMD > 0 means a beneficial outcome for clozapine (e.g. more response or less adverse events. **B** Negative symptoms. FGA first-generation antipsychotic, *k* number of studies, L long-term, M medium-term, *n* number of participants, RCT randomized controlled trial, S short-term, SGA second-generation antipsychotic. Abbreviated study descriptions: Leucht et al., 2009a [76], Leucht et al., 2009b [76], Leucht et al., 2009c [79]. For continuous outcomes, SMD > 0 means a beneficial outcome for clozapine (e.g. more response or less adverse events. **C** Overall symptoms. FGA first-generation antipsychotic, *k* number of studies, L long-term, M medium-term, *n* number of participants, RCT randomized controlled trial, S short-term, SGA second-generation antipsychotic. Abbreviated study descriptions: Leucht et al., 2009a [76], Leucht et al., 2009b [76], Leucht et al., 2009c [79]. For continuous outcomes, SMD > 0 means a beneficial outcome for clozapine (e.g. more response or less adverse events. **D** Global impression. FGA first-generation antipsychotic, *k* number of studies, L long-term, M medium-term, *n* number of participants, RCT randomized controlled trial, S short-term, SGA second-generation antipsychotic. For continuous outcomes, SMD > 0 means a beneficial outcome for clozapine (e.g. more response or less adverse events. **E** Quality of life and global functioning. CGAS Children’s Global Assessment Scale, FGA first-generation antipsychotic, *k* number of studies, L long-term, M medium-term, MLDL Münchner Lebensqualitäts–Dimensionen–Liste, *n* number of participants, RCT randomized controlled trial, S short-term, SGA second-generation antipsychotic, SWN Subjective Wellbeing under Neuroleptics Scale, WHO-QOL: WHO-Quality of life. For continuous outcomes, SMD > 0 means a beneficial outcome for clozapine (e.g. more response or less adverse events. **F** Other symptoms (cognition, hostility, depression) as a continuous outcome. BPRS Brief Psychiatric Rating Scale, CGI clinical global impressions, FGA first-generation antipsychotic, *k* number of studies, L long-term, M medium-term, *n* number of participants, PANSS Positive and Negative Syndrome Scale, RCT randomized controlled trial, S short-term, SGA second-generation antipsychotic. For continuous outcomes, SMD > 0 means a beneficial outcome for clozapine (e.g. more response or less adverse events. **G** Response to treatment. FGA first-generation antipsychotic, *k* number of studies*,* L long-term, M medium-term, *n* number of participants, RCT randomized controlled trial, S short-term, SGA second-generation antipsychotic. For dichotomous outcomes, RR > 1 means a beneficial outcome for clozapine (e.g. more response or less adverse events/dropouts). **H** Relapse. FGA first-generation antipsychotic, *k* number of studies*,* L long-term, M medium-term, *n* number of participants, RCT randomized controlled trial, S short-term, SGA second-generation antipsychotic. abbreviated study descriptions: Leucht et al., 2009a [76], Leucht et al., 2009b [76], Leucht et al., 2009c [79]. For dichotomous outcomes, RR > 1 means a beneficial outcome for clozapine (e.g. more response or less adverse events/dropouts). **I** Dropouts. FGA first-generation antipsychotic, *k* number of studies, L long***-***term, M medium-term, *n* number of participants, RCT randomized controlled trial, S short-term, SGA second-generation antipsychotic. For dichotomous outcomes, RR > 1 means a beneficial outcome for clozapine (e.g. more response or less adverse events/dropouts).

Clozapine appears to be superior to FGAs in RCTs (short, medium, and long-term) with small to medium effects sizes [48, 79, 125]. Clozapine appears to be superior to risperidone in Japanese populations with a medium effect size [29, 31]. For TRS, clozapine appears to be not significantly superior to pooled SGAs in observational studies [82], and not significantly superior to other single SGAs [100] in RCTs. When FGAs/SGAs are pooled, clozapine appears to be superior in improving positive symptoms in RCTs in TRS with a small effect size [106] see Fig. 2A).

##### Negative symptoms in schizophrenia

Clozapine is not superior to SGAs in observational studies [82], but to most FGAs in RCTs with both small and large effect sizes [48, 79, 125]—except short-term data vs chlorpromazine [100, 128]. There is conflicting evidence regarding the superiority of clozapine vs. pooled SGAs in TRS [100, 106] and clozapine appears inferior to quetiapine (short-term, only 2 studies with *n* total = 142) with medium effect sizes [29, 30, 76] and aripiprazole medium-term in RCTs with a small effect size [61] (see Fig. 2B).

##### Overall symptoms in schizophrenia

Clozapine appears to be superior to placebo in short-term RCTs with large effect sizes [76, 78], superior to FGAs in RCTs with small to medium effect sizes [44, 48, 79, 99, 125] and to SGAs in observational studies with a small effect size [82] and quetiapine in long-term RCTs with a large effect size [65]. For TRS, clozapine appears to be superior vs. CPZ with a medium effect size [100], superior vs. mixed FGAs/SGAs in RCTs with small effect sizes [85, 106], but the evidence is suggestive that clozapine is not superior vs. other antipsychotics in long-term RCTs [100, 106]. (see Fig. 2C).

##### Other efficacy measures in schizophrenia

Clozapine has a favorable profile in terms of dropout due to inefficacy compared to placebo with a large effect size [57] and to CPZ with a medium effect size [99] and SGAs, namely risperidone with medium effect sizes [29, 65, 67, 70, 76] and in terms of ACD rates compared to FGAs with small effect sizes [48, 99, 125], grouped SGAs in observational studies with a small effect size [82] and some single SGAs (e.g. risperidone and quetiapine) with small effect sizes [65] (see Fig. 2I).

With regard to relapse, clozapine appears to be superior to FGAs long-term [79, 125], but evidence from meta-analyses is inconsistent [64] (see Fig. 2H). With regard to response, clozapine appears to be superior to placebo with large effect sizes [57, 66], superior to FGAs short-term with small effect sizes [48, 99, 106, 125], but not superior to single SGAs (e.g. quetiapine, risperidone, olanzapine) [29, 30, 61, 67, 70, 122] (see Fig. 2G). As a second-line agent, clozapine appears to be superior to risperidone and other antipsychotics with small effect sizes (see Fig. 2D) [90]. Evidence does not support superiority of clozapine for hospitalization rate vs. SGAs (see Supplementary Fig. 16) or reduction of suicide/self-injurious behavior vs. SGAs in observational studies [82] (see Supplementary Fig. 17), and does not support superiority for anti-suicidal effects in long-term RCTs vs. olanzapine [29], but meta-analytic evidence from one long-term trial (*n* = 980) showed superior effects of clozapine vs. olanzapine [67] (see Supplementary Fig. 17). Meta-analytic evidence suggests superior effects of clozapine on hostility compared to FGAs in RCTs in mixed short-, medium-, and long-term RCTs with a medium effect size [50] (see Fig. 2F) and on cognition vs. SGAs in TRS in observational studies with a small effect size [82] (see Fig. 2F), whereas mostly nonsignificant effects on cognition compared to FGAs and SGAs [119] were observed in RCTs and even inferior effects vs. single FGAs, e.g. sertindole [89] (see Fig. 2F). With regard to psychosocial functioning, clozapine appears not to have significantly more beneficial effects compared to SGAs [92] (see Fig. 2E). For quality of life, available data is scarce (see Fig. 2E). A detailed report with regard to different disease entities and levels is presented in the Supplementary Results S1 (https://github.com/sksiafis/clozapine_meta_review). For additional outcomes, please see Supplementary Figs. S1–S19.

##### Other efficacy measures in BP and PDP

No superior efficacy of clozapine vs. other antipsychotics could be shown for mania in bipolar disorder short-term [45] (see Fig. 2F). For PDP, clozapine seems to be superior vs. quetiapine short-term in terms of clinical global impression with large effect sizes [51] (see Fig. 2D). A detailed report with regard to different disease entities and levels is presented in the Supplementary Results S1 (https://github.com/sksiafis/clozapine_meta_review).

### Tolerability of clozapine (SMD and RR)

**Fig. 3:**
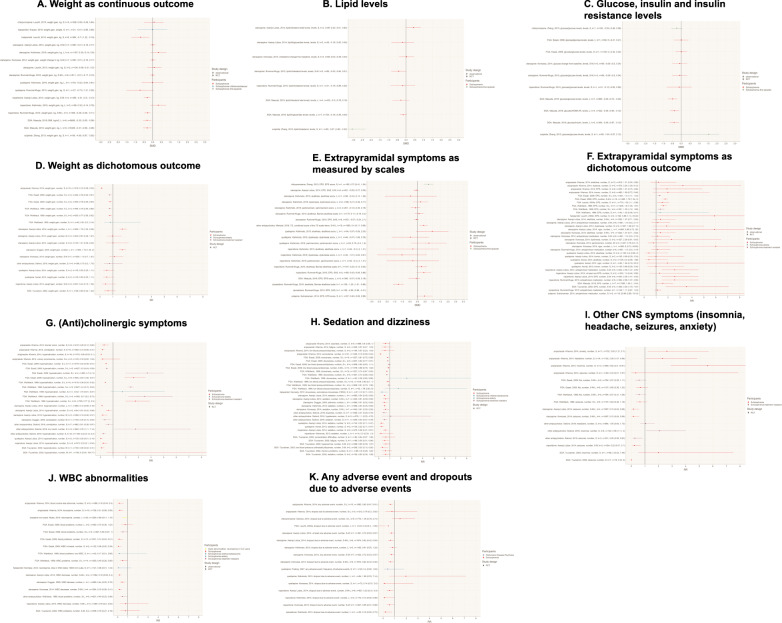
Quantitative meta-review of clozapine-specific meta-analytic data: adverse-events. **A** Weight as continuous outcome. BMI body-mass-index, *k* number of studies, kg kilogram, L long-term, M medium-term, *n* number of participants, RCT randomized-controlled trial, S short-term, SGA second-generation antipsychotic. For continuous outcomes, SMD > 0 means a beneficial outcome for clozapine (e.g. more response or less adverse events. **B** Lipid levels. *k* number of studies, L long-term, M medium-term, *n* number of participants, RCT randomized-controlled trial, S short-term, SGA second-generation antipsychotic. **C** Glucose, insulin and inulin resistance levels. FGA first-generation antipsychotic, *k* number of studies, L long-term, M medium-term, *n* number of participants, RCT randomized-controlled trial, S short-term, SGA second-generation antipsychotic. For continuous outcomes, SMD > 0 means a beneficial outcome for clozapine (e.g. more response or less adverse events. **D** Weight as dichotomous outcome. FGA first-generation antipsychotic, *k* number of studies, L long-term, M medium-term, *n* number of participants, RCT randomized-controlled trial, S short-term, SGA second-generation antipsychotic. For dichotomous outcomes, RR > 1 means a beneficial outcome for clozapine (e.g. more response or less adverse events/dropouts). **E** Extrapyramidal symptoms as measured by scales. *k* number of studies, L long-term, M medium-term, *n* number of participants, RCT randomized-controlled trial, S short-term, SAS Simpson–Angus Scale, SGA second-generation antipsychotic, TD tardive dyskinesia. For continuous outcomes, SMD > 0 means a beneficial outcome for clozapine (e.g. more response or less adverse events. **F** Extrapyramidal symptoms as dichotomous outcome. EPS extrapyramidal symptoms, FGA first-generation antipsychotic, *k* number of studies, L long-term, M medium-term, *n* number of participants, RCT randomized-controlled trial, S short-term, SGA second-generation antipsychotic. For dichotomous outcomes, RR > 1 means a beneficial outcome for clozapine (e.g. more response or less adverse events/dropouts). **G** (Anti-)cholinergic symptoms. FGA first-generation antipsychotic, *k* number of studies, L long-term, M medium-term, *n* number of participants, RCT randomized-controlled trial, S short-term, SGA second-generation antipsychotic. For dichotomous outcomes, RR > 1 means a beneficial outcome for clozapine (e.g. more response or less adverse events/dropouts). **H** Sedation and dizziness. FGA first-generation antipsychotic, *k* number of studies, L long-term, M medium-term, *n* number of participants, RCT randomized-controlled trial, S short-term, SGA second-generation antipsychotic. For dichotomous outcomes, RR > 1 means a beneficial outcome for clozapine (e.g. more response or less adverse events/dropouts). **I** Other CNS symptoms (insomnia, headache, seizures, anxiety). FGA first-generation antipsychotic, *k* number of studies, L long-term*,* M medium-term, *n* number of participants, RCT randomized-controlled trial, S short-term, SGA second-generation antipsychotic. For dichotomous outcomes, RR > 1 means a beneficial outcome for clozapine (e.g. more response or less adverse events/dropouts). **J** WBC abnormalities*.* FGA first-generation antipsychotic, *k* number of studies, L long-term, M medium***-***term, *n* number of participants, RCT randomized-controlled trial, S short-term, WBC white blood count. For dichotomous outcomes, RR > 1 means a beneficial outcome for clozapine (e.g. more response or less adverse events/dropouts). **K** Any adverse event and dropouts due to adverse events. FGA first-generation antipsychotic*,* *k* number of studies, L long-term, M medium-term, *n* number of participants, RCT randomized-controlled trial, S short-term. For dichotomous outcomes, RR > 1 means a beneficial outcome for clozapine (e.g. more response or less adverse events/dropouts).

Clozapine is equivocally associated with a significantly higher risk for weight gain with small to medium effect sizes (see Fig. 3A) and an increased risk to develop type 2 diabetes compared to most other antipsychotics [93] and with significantly fewer EPS or use of antiparkinson medication compared to FGAs with small effect sizes [48, 81, 125], SGAs [121] with large effect size and especially risperidone with a medium effect size [29, 96] (see Fig. 3E and F). Despite an unfavorable profile regarding sedation/dizziness, anticholinergic, hematological, and cardiac events, different metabolic outcomes and dropouts due to adverse events compared to both FGAs and SGAs with small to large effect sizes (see Fig. 2B, C, G, H, J, K) clozapine is associated with a significantly lower mortality [124]. A detailed report with regard to different diseases entities and levels is presented in the Supplementary Results S1 (https://github.com/sksiafis/clozapine_meta_review). For additional outcomes, please see Supplementary Figs. S1–S19.

### Efficacy of pharmacological add-on strategies to improve clozapine-related adverse-events

**Fig. 4:**
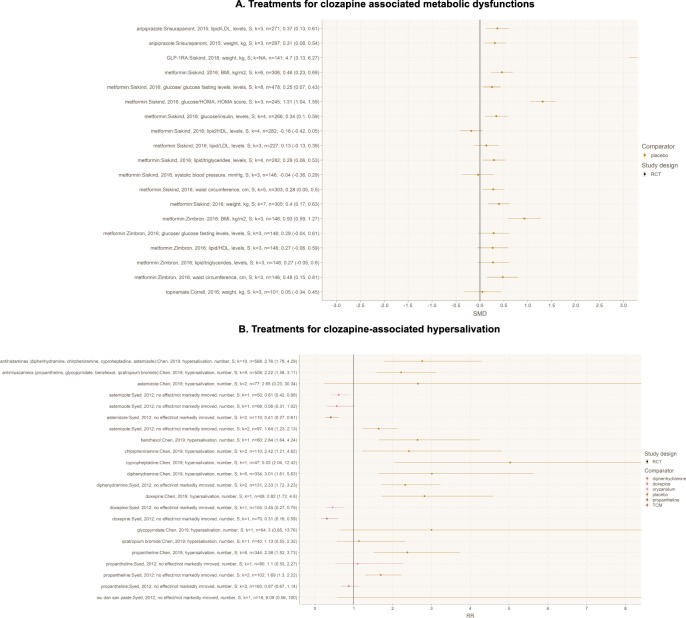
Add-on strategies for adverse-event management. **A** Treatment options for clozapine-associated metabolic dysfunctions. BMI body-mass index, cm centimeter, GLP-1RA GLP-1 receptor agonist, HDL high-density lipoprotein, HOMA homeostatic model assessment for insulin resistance, k number of studies, kg kilogram, L long-term, LDL low-density lipoprotein, M medium-term, *n* number of participants, RCT randomized-controlled trial, S short-term. For continuous outcomes, SMD > 0 means a beneficial outcome for clozapine (e.g. more response or less adverse events. **B** Treatment options for clozapine-associated hypersalivation. *k* number of studies, L long-term, M medium-term, *n* number of participants, RCT randomized-controlled trial, S short-term. For dichotomous outcomes, RR > 1 means a beneficial outcome for clozapine (e.g. more response or less adverse events/dropouts).

Metformin and GLP1-RA as add-on strategies seem promising for improving metabolic outcomes short-term with mostly small effect sizes [108, 131], but also aripiprazole appears effective in terms of short-term weight reduction and reduction of lipid levels with small effect sizes [112]. Limited evidence is available for the efficacy of topiramate for weight reduction [43]. Evidence is scarce for clozapine-related hypersalivation and constipation treatment [41, 49, 115] (see Fig. 4A, B). A detailed report with regard to different disease entities and levels is presented in the Supplementary Results S1 (https://github.com/sksiafis/clozapine_meta_review) and in the Supplementary Figs. S18, 19.

## Discussion

In our meta-review, we aimed at synthesizing all available evidence for clozapine’s efficacy and safety across all medical conditions where clozapine is used. We were able to give a systematic overview of all relevant clozapine indications and clozapine-associated endpoints derived from a total of 112 meta-analyses. Based on this overview and the methodological evaluation of all included meta-analyses, guideline developers and clinicians are now able to provide a strict risk-benefit evaluation taking into consideration all dimensions of clozapine treatment.

### Symptomatic endpoints

Clozapine is significantly superior to placebo and superior to FGAs with regard to overall and positive symptoms according to high-quality meta-analytic evidence from RCTs [48, 76]. Meta-analytic evidence suggests significant superiority of clozapine in terms of efficacy on overall and positive symptoms compared to most SGAs [29, 85, 121, 122, 125] even though results are inconsistent [79].

With regard to evidence for clozapine’s effectiveness derived from observational studies, clozapine is associated with significantly lower hospitalization and ACD rate compared with other SGAs [65, 82]. For multi-episode schizophrenia and TRS, the superiority of clozapine compared to other SGAs is challenged according to meta-analytic evidence derived from RCTs: specifically for multi-episode schizophrenia (excluding TRS), clozapine appears to be not significantly different from e.g. amisulpride, olanzapine, zotepine and risperidone in terms of overall symptoms [57]. For TRS, clozapine is presumed to be not more efficacious than olanzapine, risperidone or ziprasidone in the subanalyses including only TRS trials in overall symptoms in the meta-analysis from Leucht et al. [79] being in line with the evidence from the meta-analysis from Samara et al. [100], where also only blinded RCTs were included and clozapine was not significantly superior to most other APs with regard to overall symptom reduction [100].

For treatment-resistant positive symptoms, clozapine seems to have significantly superior beneficial effects compared to quetiapine and haloperidol on single-substance level, but not compared to olanzapine [100]. When comparators are pooled as a group (FGA + SGA) clozapine was shown to have superior effects for treatment-resistant overall and positive symptoms [85, 106]. Nevertheless, for overall and positive symptoms in TRS, inconsistent evidence is reported in meta-analyses due to differences in study selections, study populations, in the handling of study characteristics, and in methodological approaches [100, 106].

For treatment-resistant negative symptoms, clozapine was shown to be slightly superior to FGAs [48] despite inconsistent results [73], but—according to a large body of evidence—not significantly superior in comparison to SGAs [29, 85, 121], and if, then only on short-term [106]. Nevertheless, negative symptom data did not include a separation of primary from secondary negative symptoms, which hampers interpretability of the results.

For cognition and psychosocial functioning, clozapine is not presumed to be significantly superior compared to other SGAs [89, 92]. While evidence for the efficacy of clozapine for first-episode psychosis is scarce [128], limited evidence suggests superior effects for clozapine as a second-line agent compared to other antipsychotics, such as, e.g. risperidone [90].

Clozapine shows beneficial effects on psychosocial function but without superiority to other antipsychotics [92]. Inconclusive results are available for pro-cognitive effects of clozapine vs. FGAs and SGAs [89, 119, 126]. For children with schizophrenia and childhood-onset schizophrenia, clozapine seems to have superior efficacy compared with FGAs [60, 74]. Limited evidence is available for schizophrenia and comorbid depression or comorbid substance abuse, but when clozapine was compared with any other antipsychotic drug plus an antidepressant or placebo, patients treated with clozapine constantly scored better on Hamilton scores [52], and clozapine was superior to other antipsychotics in substance use [71] and to risperidone in reducing craving for cannabis [118]. Furthermore, clozapine is likely to have some beneficial effects on hostility [50], suicidal behavior [56]—and maybe aggression versus others in schizophrenia, at least when compared with FGAs [62]. Nevertheless, negative evidence for suicidal behavior and self-injurious behavior for clozapine vs. SGA in observational studies was also reported [82]. Of note, meta-analytic evidence for the efficacy of clozapine in suicidal symptoms is mainly from registry data and non-randomized trials, whereas to our knowledge, only one high-quality RCT [132] fosters the evidence and contributes to long-term RCT data [29]. With regard to dosing, there is only little meta-analytic evidence that in studies with mean clozapine dosages above 400 mg/day, clozapine was superior to risperidone, but not olanzapine [79] and evidence of effects between clozapine standard, low and very low dose regimes on overall outcome in schizophrenia is sparse [114]. For bipolar disorder, the efficacy of clozapine seems to be similar to other antipsychotics in manic episodes [45]. For neurological disorders, the largest body of evidence is available for PDP, where low-dose clozapine (range from 12.5 to 50 mg) showed beneficial effects on psychotic symptoms) [51, 58] even though negative results are reported [127].

### Non-symptomatic efficacy/effectiveness endpoints

Limited evidence hints at superior effects vs. SGAs in reducing drug abuse in schizophrenia short and medium-term [71, 118]. With regard to relapse prevention, clozapine is superior to FGAs [48, 77] and SGAs [125], even though results in the latter are inconsistent [64]. Mortality rate ratios seem to be lower in patients continuously treated with clozapine compared to patients on non-clozapine antipsychotics [82, 124]. Clozapine significantly reduces hospitalization rates compared to non-clozapine SGAs [75, 82] and all-cause discontinuation rates [65, 82].

### Clozapine-related adverse-events and complications

There is a strong body of meta-analytic evidence for especially unfavorable metabolic outcomes (e.g. weight gain) [78, 93], also for first-episode schizophrenia patients [128]. In line with meta-analytic evidence for weight gain and the increased risk for the onset of metabolic syndrome, treatment guidelines for adult patients with schizophrenia have previously suggested not to use clozapine as a first-line agent [3]. The application among elderly patients with schizophrenia remains to be further investigated [17]. Meta-analytic evidence unequivocally suggests that clozapine is associated with a lower risk for EPS and/or tardive dyskinesia compared to other FGAs and SGAs [38, 81]. Of note, meta-analytic evidence suggests clozapine as favorable therapeutic antipsychotic agent for the event of TD [83]. Clozapine use significantly increases the risk for gastrointestinal hypomotility/constipation compared to other APs [104], but no meta-analytic data is available for the prevalence of clozapine-related (sub-) ileus.

Clozapine appears to be the most unfavorable antipsychotic for sedation compared to FGAs and other SGAs [29, 78]. With regard to pneumonia, the only available meta-analytic evidence suggests that clozapine significantly increases pneumonia risk compared to no antipsychotic use [47], but in general, evidence suggests that clozapine-related pneumonia [47, 133] might be overseen.

The incidence for clozapine-associated neutropenia is presumed to be 3.8% and severe neutropenia (agranulocytosis) between 0.4% [18] and 0.9% [88], respectively according to two meta-analyses of observational studies and—according to another meta-analysis—the relative risk for neutropenia is not significantly associated with any individual clozapine add-on antipsychotic medication [87]. Death caused by clozapine-related agranulocytosis appears to be at 0.05% [18]. Meta-analytic evidence suggests a low event rate of both clozapine-related myocarditis (0.7%) and cardiomyopathia (0.6%) [16]. Nevertheless, clozapine’s potential effect to cause arrhythmia [28] might be overseen, as reflected in a low amount of evidence. For PDP, low-dose clozapine appears to be relatively safe compared to placebo with mixed results for the effects on motor symptoms [51, 58].

### Treatment of clozapine-related adverse events and complications

Metformin [108], GLP-1RAs [105] and to a lesser extent aripiprazole [112] seem to be beneficial add-on-agents for the management of clozapine-related weight gain. Metformin was superior to placebo in terms of weight loss and BMI [108]. GLP-1RAs led to a significantly higher weight loss compared to control (placebo or usual care) [105] and aripiprazole was superior with regard to weight change and LDL-cholesterol compared to placebo [112]. In all scenarios, a close risk-benefit evaluation has to be performed, since e.g. the add-on use of aripiprazole was significantly associated with agitation/akathisia and anxiety [112].

For the treatment of clozapine-related constipation, there is not enough evidence from clinical trials to inform clinical practice [49], as it is the case for clozapine-related sinustachycardia, where no data for specific clinical interventions, e.g. the use of beta-blockers is available from clinical trials [26].

The results of this meta-review should be interpreted with caution due to the inherent limitations of the meta-analyses and their included studies. The quality of meta-analyses was evaluated using the AMSTAR-2 tool, which includes items for heterogeneity and publication bias, yet further exploration of their impact on meta-analytic estimates is out of the scope of this manuscript. In addition, overlapping meta-analyses on the same topic may have different results due to different eligibility criteria and statistical methods [134], such as differences about the efficacy of clozapine for treatment-resistance schizophrenia [100, 106]. Limitations of the included studies could also impact meta-analytic estimates, i.e. a meta-analysis of observational studies investigating mortality during treatment with clozapine [135]. The potential impact of study-level (e.g. rating scale used to measure symptom improvement), and participant-level factors (such as race/ethnicity) or other confounding factors specifically in observational studies (such as concomitant medications) could not be easily addressed at the level of an umbrella review. Our meta-review represents the first comprehensive quantitative analysis of clozapine with regard to its efficacy and safety in schizophrenia, schizoaffective and bipolar disorder and PDP. Our meta-review outlines the superior efficacy of clozapine compared to FGAs and most other SGAs in schizophrenia and suggests beneficial outcomes in bipolar disorder and PDP. Nevertheless, evidence to manage clozapine-related adverse-events is sparse. In addition, more studies are needed regarding the safety of clozapine beyond the scope of schizophrenia-spectrum disorders. Our quantitative meta-review suggests that if routine hematological monitoring and screening for the early detection of myocarditis are performed, a close and continuous risk-benefit evaluation with regard to cardiovascular risk factors is key to improve clozapine-related outcomes.

## Supplementary information

Supplementary File S1

Supplementary Table 1

Supplementary Table 2

Supplementary Figures S1-S19

PRISMA Checklist
