# Supplementary material for: Efficacy and safety of clozapine in psychotic disorders—a systematic quantitative meta-review
Source: Transl Psychiatry. 2021 Sep 22;11:487. doi: 10.1038/s41398-021-01613-2 (PMC8458455; doi:10.1038/s41398-021-01613-2)
Supplement: Supplementary file 1 — Supplementary File S1 [file 41398_2021_1613_MOESM1_ESM.doc]

***Supplementary Results S1:***

***3.2.1 Impact on autonomic nervous system dysfunction***

The only available meta-analysis was from Alvares et al. (Number of studies (N)=173, number of participants (n)=13527) and investigated heart rate variability (HRV) in psychiatric disorders vs. healthy controls in observational studies. Clozapine use (N=3, n=63) had a significantly detrimental effect on HRV (Hedges g=-0.643, 95% Confidence Interval [CI]=[-0.973, -0.313], p<0.001).

***3.2.2 Efficacy in Bipolar disorder (BD)***

The only available meta-analysis was from Delgado et al. (N=3, RCTs, n=202) and investigated clozapine’s efficacy in the treatment of manic episodes in two open-label and one double-blind trial. Here, clozapine was not superior to other APs (Mean difference (MD)=0.03, 95%CI [-0.86, -0.92]).

***3.2.3 Impact on cardiological outcomes***

In a sample of N=28 studies (both observational and RCTs) of 258961 people exposed to clozapine, Siskind et al. reported a low event rate both of myocarditis (0.007, 95%CI [0.003, 0.016]) and cardiomyopathia (0.006, 95%CI [0.002, 0.023]). In a large meta-analysis of observational studies from Salvo et al., clozapine increased the risk of sudden cardiac death (SCD) and sudden unexpected death (SUD) compared to non-users (Odds Ratio (OR)=3.67, 95%CI [1.94, 6.94]) . Even SUD could also be related to non-cardiac conditions, the potential of clozapine for triggering arrhythmias seems clinically relevant according to this meta-analysis. Lally et al. investigated pharmacological interventions for clozapine-induced sinustachycardia but due to insufficient data, interventions such as beta-blockers could not be evaluated .

***3.2.4 Efficacy and tolerability on children and adolescents with schizophrenia***

***a) Efficacy***

In a recent network meta-analysis, Krause et al. (N=28 RCTs, n=3003) found that clozapine (N=2, n=22) was significantly more effective than PLC with regard to overall symptoms with a large effect size (Standard Mean Difference (SMD)=-0.88, 95%CI [-1.03, -0.73]) and outperformed all other analysed antipsychotics (APs) with SMDs between -0.71 compared to olanzapine and -1.15 compared to ziprasidone . With regard to negative symptoms, clozapine was significantly better compared to olanzapine (SMD=-0.71, 95%CI [-1.38, -0.05] and ziprasidone (SMD=-1.15, 95%CI [-1.92, -0.39] . Clozapine was significantly superior to molindone (SMD=1.13, 95%C [0.33, 1.93] and PLC (SMD=1.72, 95%CI [0.97, 2.48] with regard to positive symptoms. In total, only data from N=2 clozapine RCTs (n=22) contributed to the clozapine-specific outcomes and evidence was mostly indirect since clozapine was compared to a total of only two other drugs and data was from two short-term trials . Kumar et al. (N=13, RCTs, n=1112) compared SGAs for the treatment of psychosis among adolescents and found that no convincing evidence suggests that SGAs are superior to FGAs in this respect and clozapine-data derived mainly from a small 12-week RCT from Kumra et al. on refractory early-onset schizophrenia (n=21).

***b) Tolerability***

Krause et al. found the highest antipsychotic-related weight gain for clozapine, quetiapine and olanzapine compared to PLC (SMD=-0.92, -0.85 and -1.24 respectively). Furthermore, clozapine was significantly more sedating in a head-to-head comparison with haloperidol (N=1, n=21, OR=-3.18, 95%CI [-5.63, -0.72]) and led to significantly more sedation compared to PLC (OR=-2.97, 95%CI [-4.43, -0.54]) .

Cohen et al. (N=41, n=4015) investigated adverse effects of SGA compared to PLC among children with psychiatric disorders and found significant weight-gain for clozapine (2.38 +/- 1.13kg, 95%CI [0.19, 4.62], N=5, n=75) . Kumar et al. found that SGAs might be more acceptable to young people because fewer symptomatic adverse events are seen in the short term, but clozapine-specific data were again mostly from Kumra et al. (n=21) .

***3.2.5 Childhood-onset schizophrenia***

***a) Efficacy***

In a Cochrane review from Kennedy et al. , a few results from one study favoured clozapine over haloperidol in treating treatment-resistant childhood-onset schizophrenia (n=21, WMD CGAS=17.00, 95%CI [7.74, 26.26], n=21, WMD Bunney-Hamburg Psychosis Rating Scale=-3.60, 95%CI [-6.64, -0.56]) .

***b) Tolerability***

Participants on clozapine were three times more likely to have drowsiness (1 RCT, n=21, RR=3.30, 95%CI [1.23, 8.85] and half of the children receiving clozapine developed neutropenia (1 RCT, n=21, RR=12, 95%CI [0.75, 192.86]) .

***3.2.6 Impact on cognition in schizophrenia-spectrum***

The meta-analysis from Nielsen et al. included all cognitive tests and categorized them under specific predefined domains in contrast to another meta-analysis . The analyses from Nielsen et al. showed decreased verbal working memory in clozapine patients compared to ziprasidone. On executive function, sertindole performed better than clozapine (ES=0.82, 95%CI [0.06, 1.58]). On long-term verbal working memory, clozapine performed poorer than olanzapine (ES=0.41, 95%CI [0.06, 0.76]). On verbal fluency, FGA performed poorer than clozapine (ES=0.44, 95%CI [0.06, 0.81]). With regard to visuospatial skill, clozapine performed poorer compared to olanzapine (ES=0.44, 95%CI [0.05, 0.83]) . Tuunainen et al. reported non-significant differences between SGAs and clozapine with regard to short-term memory (Peto OR=0.59, 95%CI [0.25, 1.37], N=1, n=135, p=0.22) and Asenjo-Lobos et al. meta-analyzed a medium-term study with n=79 participants and found a significant advantage for olanzapine vs clozapine with regard to cognitive functioning (RR=1.64, 95%CI [1.15, 2.35], p<0.01) . The same analysis was repeated in the meta-analysis from Komossa et al. . In the meta-analysis from Essali et al., cognitive impairment from one small study (n=82) favoured the clozapine group who experienced less impairment compared to FGAs (RR=0.56 95%CI [0.3, 0.9], NNT=4 95%CI [3, 21]) when assessed with the SKT scale .

***3.2.7 Impact on clozapine-induced constipation***

***a) Prevalence and predictors***

Shirazi et al. meta-analyzed N=32 studies and found a pooled prevalence of clozapine-associated constipation of 31.2% (95%CI [25.6, 37.4]) (n=2013) and a significantly increased for constipation when taking clozapine compared to other APs (OR=3.02, 95%CI [1.91, 4.77], p<0.001, N=11). Meta-regression showed significantly higher rates in inpatient settings vs. outpatient or mixed settings (p=0.02) and in those studies were constipation was primary or secondary outcome (p=0.048) .

***b) Pharmacological treatment of clozapine-induced constipation***

The meta-analysis from Every-Palmer et al. included a total of n=480 participants with n=306 on clozapine from two Chinese studies with an overall high risk of bias, where data suggested that mannitol was more effective than rhubarb soda and phenolphthalein and a two-week course of glycerol suppositories was less effective than traditional Chinese medicine approaches . There were no data comparing common pharmacological interventions for constipation, such as lactulose, polyethylene glycol, stool softeners, lubricant laxatives or of novel treatments, such as linaclotide , and thus the grade of evidence must be considered very low.

***3.2.8.1 All-cause discontinuation (ACD) in observational studies***

In a large meta-analysis of 63 nonrandomized cohort studies (n=109341) with an average study duration of 19.1 months, clozapine was significantly associated with a lower rate of ACD compared to non-clozapine SGAs (16 studies, n=56368, RR=0.732, 95%CI [0.639, 0.838], p<0.001) despite significantly greater illness severity (p=0.04) . In the meta-analysis from Soares-Weiser, N=60 RCTs (n=33360) and 27 observational studies (n=202591) were pooled . No significant differences between olanzapine and clozapine with regard to discontinuation rate could be shown (N=8 RCTs, n=1754, p=0.64). Nevertheless, olanzapine was less effective than clozapine in observational studies with a higher rate of discontinuation (RR=1.30, 95%CI [1.03, 1.64], N=9, n=13911) . Overall, the findings from Soares et al. suggest a relative superiority of clozapine compared to other APs .

In the meta-analysis from Kishimoto et al., a meta-analysis of N=59 RCTs (n=45787) lasting ≥6 months (mean=47.4 weeks) comparing SGAs head-to-head, clozapine and olanzapine and risperidone were significantly superior to several other SGAs (p<0.05) with regard to ACD as primary outcome .

***3.2.8.2 ACD in RCTs***

In the meta-analysis from Asenjo-Lobos et al., no significant difference for the outcome ACD was found for clozapine vs. olanzapine (N=11 RCTs, n=1702, RR=1.04, 95%CI [0.93, 1.17]) .

The finding was similar in the meta-analysis from Leucht et al. , where clozapine and olanzapine were among the three best drugs (with amisulpride) when compared with PLC in terms of ACD in RCTs in acute schizophrenia . In the meta-analysis from Essali et al., short-term data from N=32 studies (n=2316) indicated that signiﬁcantly less participants given clozapine left the study early (RR=0.81, 95%CI [0.7, 1.0], NNT=35, 95%CI [20, 217]) compared to FGAs. Also longer-term data from six studies showed a signiﬁcant beneﬁt in the clozapine group (n= 982, RR=0.60 95%CI [0.5, 0.7], NNT=15, 95%CI [12, 20]) . This is line with the findings from Samara et al., where signiﬁcantly fewer participants on clozapine discontinued treatment compared to chlorpromazine (clozapine: N=10, n=1046, RR=1.34, 95%CI [1.08, 1.67] . Even though in the meta-analysis from Wahlbeck et al., the acceptability of the treatment as measured by number of people dropping out of the heterogeneous studies with 1084 treatment-resistant people (n=7) did not significantly diﬀer between clozapine and FGAs (OR=0.7, 95%CI [0.3, 1.4]), the two long-term studies with 648 people significantly favoured clozapine (OR=0.3 95%CI [0.2, 0.4], NNT=3.3 95%CI [2.7, 4.4]) .

In a subanalysis within the network-metanalysis on MES from Huhn et al. (N=226, n=42672), clozapine was superior to PLC in terms with regard to ACD (n=440) (RR=0.76, 95%CI [0.59, 0.92]) but was also outperformed by amisulpride, paliperidone, olanzapine and clopentixol (with widely differing sample sizes) (see paragraph MES for more details).

***3.2.9 Dosing and minimum effective dose***

Leucht et al. found in their review of 73 studies the minimum effective dose for clozapine to be 300mg per day but the result is questionable since it is based on a single small study comparing clozapine 300 mg per day with 100mg per day but not with placebo (PLC) . Another meta-analysis from Subramanian et al. included 5 studies comparing the effects of clozapine at very low dose (149 mg/day), low dose (150 mg/day to 300 mg/day) and standard dose (301 mg/day to 600 mg/day) with four studies based on small number of participants . The reported evidence in these trials was estimated of low of very low quality . Thus, minimum effective dose, especially with regard to different populations in schizophrenia-spectrum, remains unclear in the absence of high-quality studies. With regard to the disposition of clozapine, Tsuda et al. included four association studies of clozapine comprising 196 patients (120 smokers, 76 non-smokers) with schizophrenia or other psychiatric disorders in a small meta-analysis without controlling for CYP-confounding comedication. The concentration/dose ratio was significantly lower in smokers compared to non-smokers (p<0.00001, mean difference (MD)=-1.11 (ng/ml)/(mg/day), 95%CI [-1.53, -0.70]). It was estimated that if 200 and 400mg/day of clozapine would be administered to smokers, about 100 and 200mg/day respectively, should be administered to non-smokers . In a meta-analysis from Leucht et al. , in studies with mean clozapine dosages above 400mg/day, clozapine was superior to risperidone (N=2, n=335, WMD=-6.6, 95%CI [-11.5, -1.7], but not olanzapine (N=2, n=154, WMD=2.4, 95%CI [-2.4, 7.3] and data for other antipsychotics was not available .

***3.2.10 Schizophrenia (Non-FEP and non-TRS)***

***a) Predictors of response***

In a meta-analysis from Okhuijsen-Pfeifer et al. (N=34, n=9386), lower age, lower PANSS negative scores and paranoid schizophrenia subtype at baseline were identified as significant predictors of clozapine response in observational studies.

***b) Efficacy in acute schizophrenia vs PLC***

In the meta-analysis from Leucht et al. (excluding TRS trials, trials done in stable patients or patients with predominant negative symptoms or concomitant medical illness) (N=212 RCTs, n=43049), clozapine was significantly superior to PLC with regard to overall symptoms (SMD=0.88, 95%CI [0.73, 1.03]) (see paragraph on acute MES).

**c) *Tolerability in acute schizophrenia vs PLC***

In the meta-analysis from Leucht et al. (N=212 RCTs, n=43049) clozapine was the best drug compared with placebo for EPS (OR=0.30, 95%CI [0.12, 0.62]) and the most unfavorable drug for sedation (OR=8.82, 95%CI [4.72, 15.06]) . Furthermore, it was the third most unfavorable drug for weight gain (OR=0.65, 95%CI [0.31, 0.99]) (see paragraph on acute MES).

***d) Efficacy in schizophrenia vs PLC***

In the meta-analysis from Leucht et al. , one small study (n=22) was meta-analyzed with an ES of -1.64 (95%CI [-2.61, -0.68]) for clozapine vs. PLC with regard to overall symptoms (see paragraph on acute MES).

***e) Tolerability in schizophrenia vs PLC***

For clozapine, insufficient data for conducting a meta-analysis was available (see paragraph on acute MES).

***f) Efficacy FGA vs. clozapine in schizophrenia***

In the meta-analysis from Samara et al., clozapine outperformed chlorpromazine (CPZ) (N=7, n=824, RR=0.60 95%CI [0.40, 0.91], p=0.02) with regard to treatment response . In the meta-analysis from Leucht et al. (N=150 RCTs, n=21533) that included mostly short-term studies, clozapine was significantly superior to FGAs for overall efficacy with an ES of -0.52 (95%CI [-0.75, -0.29], p<0.0001) . Furthermore, clozapine significantly outperformed FGAs in terms of positive, negative and depressive symptoms, relapse, and quality of life . In the meta-analysis from Essali et al., clinical improvements were seen more frequently in those taking clozapine (n=1119, 14 RCTs, RR=0.72, 95%CI [0.7, 0.8], NNT=6, 95%CI [5, 8]). BPRS scores showed a greater reduction of symptoms in clozapine-treated participants, (n=1205, 17 RCTs, WMD=-3.79, 95%CI [-4.9, -2.7]) and short-term data from the SANS negative symptom scores favored clozapine (n=196, 6 RCTs, WMD=-7.21, 95%CI [-8.9, -5.6]) .

**g) *Tolerability FGA vs. clozapine in schizophrenia***

In the meta-analysis from Leucht et al. , clozapine was significantly superior to haloperidol and low-potency FGA with lower rates of EPS (N=3, n=162, RR=0.17, CI [0.03-0.88], p=0.035 and N=11, n=775, RR=0.66, 95%CI [0.48, 0.91], p=0.010) . In terms of weight gain, clozapine was significantly more associated with weight-gain compared to vs. haloperidol (N=3, n=170, MD=3.4, 95%CI [2.0, 4.9], p<0.0001) but not significantly more when compared with low-potency FGA (N=3, n=232, MD=0.3, 95%CI [-1.6, 2.2], p=0.753). Clozapine was furthermore associated with significantly higher rates for sedation compared to haloperidol (N=6, n=655, RR=1.50, 95%CI [1.01, 2.23], p=0.043) and vs. low-potency FGA (N=9, n=928, RR=1.32, 95%CI [1.10, 1.59], p=0.003) .

***h) Efficacy SGA vs. clozapine in schizophrenia***

In the SGA head-to-head comparison meta-analysis from Leucht et al., clozapine (n=28) was not significantly different from olanzapine (n=619), quetiapine (n=232), risperidone (n=466) and ziprasidone (n=146), but significantly more efficacious than zotepine (n=59, WMD=-6.9, p=0.002) with regard to overall symptoms . Clozapine-specific results for positive symptoms paralleled those found for overall symptoms with the exception that clozapine and zotepine could not be compared head-to-head due to lack of available data .

***i) Tolerability SGA vs. clozapine in schizophrenia***

Asenjo Lobos et al. conducted a clozapine-specific meta-analysis of 27 blinded RCTs (n=3099) and found that clozapine produced somewhat fewer EPS than risperidone (N=6, n=304, RR=0.39, 95%CI [0.22, 0.68]) and zotepine (n=59, RR=0.05, 95%CI [0.00, 0.86]), but no significant difference was found for the clozapine vs. olanzapine comparison (6 RCTs, n=561, RR=0.87, 95%CI [0.46, 1.67]) . For the outcome leaving the study early due to adverse events, clozapine was associated with a higher rate in a head-to-head comparison with olanzapine (N=9 RCTs, n=1674, RR=1.60, 95%CI [1.07, 2.40]) .

In general, clozapine-treated patients were more likely to develop significantly decreased white blood cell counts (WBC) compared to olanzapine (N=4 RCTs, n=1264, RR=5.68, 95%CI [2.48, 13.00]), more hypersalivation and sedation compared to olanzapine (e.g. hypersalivation long-term N=1 RCT, n=980, RR=8.18, 95%CI [5.64, 11.86]), risperidone (e.g. hypersalivation N=3 RCTs, n=373, RR=4.38, 95%CI [18.86, 10.30]) and quetiapine (e.g. hypersalivation: N=2 RCTs, n=135, RR=33.91, 95%CI [6.96, 165.24]) and more seizures than patients on olanzapine (4 RCTs, n=1097, RR=6.50, 95%CI [1.73, 24.47]) and risperidone (N=2 RCTs, n=354, RR=4.47, 95%CI [1.43, 14.01]) . Clozapine produced more weight gain compared to risperidone (N=1 RCT, n=40, RR=23, 95%CI [1.45, 365.61]), but not compared to olanzapine in short- medium and long-term (e.g. long-term 1 RCT, n=980, RR=0.57, 95%CI [0.48, 0.66]) . No significant difference was found for the outcome significant increase of glucose levels between clozapine and olanzapine (N=1 RCT, n=980, RR=0.76, 95%CI [0.40, 1.44]) (for more details, see Metabolic outcomes).

***j) Efficacy clozapine vs. non-clozapine APs***

In a large meta-analysis of N=63 nonrandomized cohort studies (n=109341) with an average study duration of 19.1 months clozapine was significantly associated with better outcomes regarding overall symptoms (SMD=-0.302; 95%CI [-0.572, -0.032]; p=0.03) and Clinical Global Impressions Scale Severity (CGI-S) (SMD=-1.182, 95%CI [-2.243, -0.122], p=0.03) compared to non-clozapine APs . In a meta-analysis of Mizuno et al., Clozapine was superior to other antipsychotics in improving total symptoms in non-TRS studies (g=0.20; 95%CI [0.08, 0.32]) . Clozapine showed greater improvement in positive symptoms in non-TRS studies (g=0.15; 95%CI [0.04, 0.25]; p=0.006). For negative symptoms, the effects were not significantly superior in non-TRS studies (g=0.07; 95%CI [−0.05, 0.19] p=0.262) .

***3.2.11 Acute multi-episode schizophrenia (MES)***

***a) Efficacy***

In the network meta-analysis from Huhn et al. , 32 APs were compared in N=402 RCTs. With regard to overall symptoms, clozapine significantly outperformed PLC treatment (n=40815) (SMD=-0.89, 95%CI [-1.08, -0.71]) and was significantly more efficacious (with amisulpride, olanzapine, zotepine and risperidone) than other antipsychotics. Clozapine outperformed many other drugs and significantly reduced negative symptoms compared with PLC (n=159) (SMD=-0.62, 95%CI [-0.84, -0.39]) – an association that – according to the authors- could be biased since only n=159 could be included in the analyses since TRS trials were excluded from the authors where clozapine is mainly applied and investigated . Clozapine significantly outperformed PLC in ACD (n=440) (SMD=0.74, 95%CI [0.59, 0.92]) . Clozapine was associated with significantly more reduction of depressive symptoms compared with many other drugs .

***b) Tolerability***

Clozapine produced significantly more sedation compared to PLC (n=347), RR=3.02, CI [2.52, 3.37], significantly more at least one anticholinergic side-effect vs. PLC (n=302), RR=2.21, 95%CI [1.26, 3.47], more weight gain in kg (n=113), MD=1.89, 95%CI [0.36, 3.43], and use of antiparkinson medication (n=144) (RR=0.46, 95%CI [0.19, 0.88]) and with low rates of akathisia (n=11) (RR 0.17, 95%CI [0.00, 1.34) and even prolactin decrease compared with PLC (with wide CIs) (n=24) (MD=-77.05, 95%CI [-120.23, -33.54] .

***3.2.12 Elderly patients with schizophrenia***

Krause et al. found in pairwise meta-analysis of N=18 RCTs in n=1225 elderly patients (minimum age 46-65, mean age 57-73 years) that clozapine was not superior to CPZ with regard to overall symptoms (N=1, SMD=0.15, 95%CI [-0.58, -0.88]) .

- - 1. ***EPS***

***a) Clozapine vs. low-potency conventional AP (FGA)***

In the meta-analysis from Leucht et al. (N=31, n=2320) clozapine was associated with significantly fewer EPS compared to FGA (RD=-0.15, 95%CI [-0.26, -0.4], p=0.008) .

***b) Clozapine vs. SGA***

In head-to-head meta-analysis from Rummel-Kluge et al., clozapine had significantly less use of antiparkinson medication than risperidone (N=6, n=304, RR=0.39, 95%CI [0.22, 0.68], p=0.0009) and zotepine (N=1, n=59, RR=0.05, 95%CI [0, 0.85] . No difference was found compared with olanzapine (N=6, n=561, RR=0.87, 95%CI [0.46, 1.67], p=0.69) and ziprasidone (N=1, n=146, RR=0.9, 95%CI [0.7, 1.15], p=0.39). No significant difference was found with regard to akathisia between clozapine and olanzapine (N=1, n=175, MD=-0.1, 95%CI [-0.38, 0.18], p=0.49), and between clozapine and risperidone (N=1, n=107, MD=-0.2, 95%CI [-0.5, 9.1], p=0.19), but between clozapine and ziprasidone (N=1, n=139, MD=0.15, 95%CI [0.11, 0.19], p<0.00001) .

***c) Tardive dyskinesia (TD) risk in FGA and SGA***

In the high-quality meta-analysis from Bergman et al., no clozapine-specific data could be extracted regarding clozapine-associated risk for TD . In the meta-analysis from Carbon et al., no difference was found between probable treatment-emergent TD between clozapine and non-clozapine SGAs in three pooled studies (two studies vs. olanzapine and one vs. mixed SGAs) (RR=1.07, 95%CI [0.49, 2.34], p=0.86). SGA comparisons confirmed that agents with the lowest EPS risks, i.e. clozapine and olanzapine, also have the lowest TD risk .

***d)Treatment of TD***

According to the Cochrane review from Bergman et al., no data derived from RCTs can currently support the notion that any particular antipsychotic is an effective treatment for TD. For clozapine, it remains to been seen if it is associated with lower incidence rates of TD .

***3.2.14 First-episode schizophrenia spectrum***

***a) Clozapine vs. FGA - Efficacy***

In the meta-analysis from Zhang et al., clozapine was not associated with significantly different ACD rates, increase in total symptom reduction or positive symptom reduction compared to FGAs. Clozapine outperformed CPZ in terms of negative symptoms (ES=0.41, p<0.01) .

***b) Clozapine vs. FGA - Tolerability***

Clozapine produced less frequent akathisia and/or co-treatment with anticholinergics and benzodiazepines than FGAs, but weight gain was greater compared with FGA comparators and clozapine produced greater increases in glucose and lipid parameters . In the meta-analysis from Tek et al. on long-term trials (≥12 weeks), clozapine (and olanzapine) were the APs associated with the highest increase in weight-gain compared to PLC (N=2, MD=7.19kg, 95%CI [0.28, 14.09]), p=0.041) .

***3.2.15 Hospitalisation***

In the large meta-analysis from Land et al. with a great majority of observational studies, clozapine significantly reduced the proportion of people hospitalized compared to control medicines (RR=0.74, 95%CI [0.69, 0.80], p<0.001, N=22, n=44718). Clozapine and control medicines (FGAs and SGAs) had a similar time to rehospitalization (-19.90 days, 95%CI [-62.42, 22.63 days], p=0.36) . With regard to FGAs in long-term RCTs, no signiﬁcant advantage for clozapine (n=648, N=2, RR=0.94 95%CI [0.9, 1.0]) was found in the meta-analysis from Essali et al. . Meta-analyzing one long-term RCT, Asenjo-Lobos et al. found that significantly fewer people taking clozapine (20%) were hospitalised compared to those taking olanzapine (26%) (N=1, n=980, RR=0.78 CI [0.62, 0.98], NNT=18 CI [9, 230]) .

In a large meta-analysis of 63 nonrandomized cohort studies (n=109341) with an average study duration of 19.1 months, clozapine was significantly associated with lower hospitalisation risk (N=19, n=49453, RR=0.817, 95%CI [0.725, 0.920], p=0.001) .

***3.2.16 Clozapine-induced hypersalivation – Efficacy of treatment strategies***

In the meta-analysis from Chen et al. (N=19 RCTs), improvement was seen for the agents propantheline (N=6, RR=2.38, 95%CI [1.52, 3.73]), diphenhydramine (N=5, RR=3.09, 95%CI [2.36, 4.03]), chlorpheniramine (N=2, RR=2.37, 95%CI [1.59–3.55]), and benzamide derivatives (OR=6.93, 95%CI [3.03, 15.86]). When meta-analyses were limited to high-quality studies, all these results remained significant .

In a previous Cochrane review from Syed et al. (N=15), antimuscarinics were the most commonly evaluated drugs and overall study quality was reported to be poor. The authors concluded that there are currently insufficient data to confidently inform clinical practice and well-designed RCTs are needed .

***3.2.17 Clozapine for psychotic disorders in adults with intellectual disabilities***

No studies met the inclusion criteria in the Cochrane review from Ayub et al., so currently no evidence is available .

***3.2.18 Metabolic outcomes***

In a large meta-analysis of 63 nonrandomized cohort studies (n=109341) with an average study duration of 19.1 months , clozapine was significantly associated with increases in body weight (MD=1.70, 95%CI [0.31, 3.08 kg], p=0.02), body-mass index (MD 0.96, 95%CI [0.24, 1.68; p=0.009) and type 2 diabetes (RR=1.777; 95%CI [1.229, 2.570]; p=0.002) compared to non-clozapine APs .

In another large meta-analysis from Pillinger et al. including N=100 RCTs with a median duration of 6 weeks MDs for clozapine vs. PLC were 3.01kg (95%CI [1.78, 4.24]) for weight-gain, 0.56mmol/l (CI [0.26, 0.86]) for total cholesterine, 0.98 mmol/l ([0.48, 1.49]) for triglycerides, and 1.05mmol/L (95%CI [0.41, 1.70]) for glucose. In general, clozapine and olanzapine showed the worst metabolic profiles of all 18 APs . In the meta-analysis from Vancampfort et al. the risk for Metabolic Syndrome (MetS) in people with psychiatric disorders was significantly higher with clozapine and olanzapine (except vs. clozapine) than with other APs. Odds ratios for MetS were significantly elevated for people treated with clozapine compared with antipsychotic-naïve patients (OR=7.81, 95%CI [6.02, 10.22], N=22, n=2398, p<0.001), patients on amisulpride (OR=2.02, 95%CI [1.45, 2.83], N=17, n=1177) or on aripiprazole (OR=2.40, 95%CI [1.91, 3.03], N=18, n=2091). This is in line with meta-analytic evidence from Rummel-Kluge et al. , where clozapine produced significantly more weight-gain in head-to-head comparison with risperidone (N=4, n=459, MD=2.86 kg). Here, no significant differences were found between clozapine and olanzapine (N=8, n=611) for the outcome weight-gain .

- - 1. ***Metabolic outcomes – Pharmacological interventions for patients on clozapine***

***a) Metformin***

Siskind et al. conducted a meta-analysis including N=8 studies (n=478) and found that Metformin was superior to PLC in terms of weight loss (-3.12kg, 95%CI [-4.88, -1.37]) and BMI (-1.18kg/m^2^). Furthermore, clozapine significantly improved three of the five components of MetS (waist circumference, fasting glucose and triglycerides) . There were insufficient data to comment on rates of adverse events .

***b) Glucagon-like peptide-1 receptor agonists (GLP-1RA)***

Siskind et al. conducted a meta-analysis including three studies (exenatide once-weekly=2, liraglutide once-daily=1) (N=3, n=164) where after an average of 16.2 weeks (mean) of treatment, weight loss was significantly higher for GLP-1RA vs. control (PLC or usual care) (3.71kg, 95%CI [2.44, 4.99 kg], p<0.001). Furthermore, waist circumference, BMI, HbA1c, fasting-glucose and visceral-adiposity were each significantly lower with GLP-1RA. In terms of adverse events, the authors reported that patients on GLP-1RAs reported significantly more nausea compared to controls (53.6% vs. 27.5%, p=0.002), with a number needed to harm of 3.8 (95%CI [2.4, 9.7]) .

***c)Aripiprazole add-on***

Srisurapanont et al. included N=4 short-term RCTs (8-24 weeks) (n=347) and despite of no benefit on fasting plasma glucose, triglyceride and high-density lipoprotein, aripiprazole add-on treatment to clozapine was superior for weight change compared to PLC with a MD of -1.36 kg (95%CI [-2.35, -0.36]) and LDL-cholesterol with a MD of -11.06 mg/dL (95%CI [-18.25, -3.87]). Add-on treatment was significantly associated with agitation/akathisia (RR=7.59, 95%CI [1.43, 40.18]) and anxiety (RR=2.70, 95%CI [1.02, 7.15]) .

***d)Topiramate add-on***

Zheng et al. found that Topiramate co-treatment for weight loss was not efficacious in clozapine-treated patients (N=4, n=159, WMD=-1.58 95%CI [-3.93, 0.67], p=0.19) .

***3.2.20 Mortality***

In the meta-analysis from Vermeulen et al., N=24 studies reported on 1327 deaths from any causes during 217691 patient years in people treated with clozapine. The authors reported on unadjusted mortality rates in N=22 samples during a follow-up period of 1.1-12.5 years (median=5.4years) of 6.7 (95%CI [5.4, 7.9]) per 1000 patient years. Mortality rate ratios were significantly lower in patients continuously treated with clozapine compared to patients with other APs (mortality rate ratio=0.56, 95%CI [0.36, 0.85], p=0.007). Thus, clozapine treatment was associated with a significantly lower long-term all-cause mortality rate compared to other AP use .

***3.2.21 Predominant negative symptoms in schizophrenia***

Krause et al. meta-analyzed outcomes for predominant negative symptoms and clozapine was not superior to haloperidol (N=1, n=21) in terms of negative symptoms (SMD=-0.83 (95%CI [-1.73, 0.07]) .

***3.2.22 Neutropenia***

***a) Epidemiology of clozapine-associated neutropenia***

In the meta-analysis of observational studies from Myles et al. (N=108, n=119592), the incidence of clozapine-associated neutropenia was 3.8% (95%CI [2.7, 5.2%]) and severe neutropenia 0.9% (95%CI [0.7, 1.1%]). The incidence of death due to neutropenia after clozapine-treatment was 0.013% (95%CI [0.01, 0.017%]). The case fatality rate of severe neutropenia was 2.1% (95%CI [1.6, 2.8%]). The authors conclude that the peak incidence of severe neutropenia occurred at one month of clozapine-exposure and declined to negligible levels after one year of clozapine treatment . In a recent meta-analysis from Li et al., N=6 studies with 260948 clozapine-treated patients were included and an overall prevalence of agranulocytosis and death caused by agranulocytosis was estimated to be 0.4% (95% CI 0.3–0.6%) and 0.05% (95% CI 0.030.09%), respectively .

***b) Association between clozapine and other APs on development of neutropenia***

In a meta-analysis of 20 controlled studies, the risk ratio was not significantly increased in clozapine-exposed groups compared to exposure to other AP medications (Mantel-Haenszel- RR=1.45, 95%CI [0.87, 2.42]) . This finding also applied to severe neutropenia (ANC count <500 per μl) when compared to non-clozapine APs (M-H-RR=1.65, 95%CI [0.58, 4.71]). The relative risk of neutropenia associated with clozapine exposure was not significantly associated with any individual AP medication .

***3.2.23 Parkinson’s disease (PD) and Parkinson’s disease psychosis (PDP)***

In a network meta-analysis from Iketani et al. on treatment options for (drug-induced) PDP, n=64 clozapine-treated patients from four trials could be included . Clozapine treatment was effective in reduction of Brief Psychiatric Rating Scale (BPRS) scores (MD=-2.0, 95%CI [-6.7, 2.7], p=0.70) and the estimate indicated that clozapine could improve PDP when compared to PLC. In terms of safety, point estimates for the Unified PD rating scales parts III (UPDRS-III) suggested that clozapine leads to the smallest deterioration of motor function, although it is inferior to PLC (MD=0.7; 95%CI [-3.8, 4.3], p=0.29) . In the head-to-head comparison in the meta-analysis from Frieling et al. N=2 studies comparing low-dose clozapine (mean=35.8 mg, range 12.5 to 50mg; mean=24.7mg, range 6.25 to 50mg respectively) vs. PLC and one study comparing clozapine vs. quetiapine were meta-analyzed . In the clozapine vs. PLC comparison of the first study, there was no significant difference in the relative risk of leaving the study early between both arms (n=120, RR=0.61, 95%CI [0.27, 1.36]). Patients on clozapine improved significantly in the CGI-scores compared to PLC (WMD=-1.1, 95%CI [-1.24, -0.97]). In the first study, a significant improvement was reported for psychotic symptoms assessed with the BPRS (WMD=-6.70, CI [-7.45, -5.95]) favouring the clozapine group and in the second, similar results were reported for the outcome PANSS positive score (WMD:-4.80, 95%CI [-6.50, -3.10]) . Pooled UPDRS total and motor scores both significantly improved in the clozapine group (WMD=-2.39, 95%CI [-3.58, -1.20] and WMD=-1.74, 95%CI [-2.57, -0.92] respectively) . Nevertheless, in another trial (n=40) no significant difference was found between clozapine in a mean dosage of 26 mg and quetiapine in a mean dosage of 91mg with regard to CGI, BPRS, UPDRS motor scores and frequency of adverse events .

***3.2.24 Pneumonia***

Among other APs, clozapine was associated in a meta-analysis of N=14 observational studies from Dzahini et al. with a significantly increased pneumonia risk compared to no antipsychotic use .

***3.2.25 Psychosocial outcomes in schizophrenia***

In the only available meta-analysis from Olagunju et al. (N=9, n=1279), clozapine showed beneficial effects on psychosocial outcomes, but both short-term trials (less than 3 months, N=3) and long-term trials (N=5) showed no superiority of clozapine vs. other APs. The authors conclude that clozapine does not appear superior to other APs for improvement of psychosocial function .

***3.2.26 Relapse rates in schizophrenia***

In the meta-analysis from Leucht et al. , no significant difference was found for clozapine (N=3) in terms of 1-year relapse risk compared with FGAs (pooled risk of 18 vs. 25%, RD=-0.08, 95%CI [-0.19, 0.04], p=0.18) . In a meta-analysis from Kishimoto et al., where specific timepoints were investigated in a subanalysis of clozapine vs. FGAs, clozapine was associated with significantly lower relapse rates at 3 months (p=0.02) and 6 months (p=0.006) compared with FGAs . In the meta-analysis from Essali et al., patients on clozapine had fewer relapses than those on FGAs (n=1303, RR=0.62, 95%CI [0.5, 0.8], NNT 21, 95%CI [15, 49]) .

***3.2.27 First-line treatment in schizophrenia***

In the meta-analysis from Okhuijsen et al., a sensitivity meta-analysis was performed on clozapine vs. risperidone as this antipsychotic was most often compared with clozapine as a first-line treatment . This meta-analysis revealed a significant benefit of clozapine over risperidone (Hedges’ g = 0.274, 95%CI [0.027, 0.521], p = 0.030), with no evidence of heterogeneity .

***3.2.28 Second-line treatment in schizophrenia***

In the meta-analysis from Okhuijsen-Pfeifer et al. (N=15, n=314 clozapine vs n=800 non-clozapine), that investigated an early application of clozapine as first- or second-line treatment in schizophrenia compared with a miscellaneous group of APs revealed a significant benefit of clozapine (Hedges’s g=0.220, 95%CI [0.026, 0.414], p=0.026,), but when only RCTs where included, beneficial effects became non-significant .

***3.2.29 Comorbid substance abuse in schizophrenia***

In the meta-analysis from Krause et al., clozapine was superior to “any other AP” for reduction of substance use in one small study (N=1, n=31, SMD-1.08, 95%CI [-1.84 to -0.32). Of note, clozapine, olanzapine and risperidone showed superiority for symptom reduction compared to some other drugs. With regard to overall symptoms, there was no significant difference between clozapine and ziprasidone (n=25, SMD=-0.01, 95%CI [-0.81, 0.79], and with regard to overall symptoms, there were no significant differences between clozapine and risperidone as well as ziprasidone based on small single studies . With regard to negative symptoms, clozapine was significantly better than risperidone (n=36, SMD=-0.77, 95%CI [-1.46, -0.09], but not compared to ziprasidone. One study showed a significant superiority for ziprasidone compared to clozapine with regard to dropouts due to adverse events (n=30, OR=12.60, 95%CI [2.00, 79.44]). In general, adverse events were mainly based on max. two studies, so that data is scarce . In a meta-analysis from Temmingh et al. , no clear differences were found for clozapine vs. risperidone in the reduction of positive symptoms (n=36, MD=0.90, 95%CI [-2.21, 4.01]), but quality of evidence was graded as very low. clozapine was associated with lower levels of craving for cannabis vs. risperidone (N=1 RCT, n=28, MD=7.00, 95%CI [2.37, 11.63]) with also here quality of evidence was graded as very low .

***3.2.30 Suicidality and self-injurious behaviour in schizophrenia***

In a meta-analysis of observational studies (N=6, n=19700) from Masuda et al., clozapine was not significantly superior to non-clozapine SGAs regarding suicide attempts or self-injurious behaviour (RR=0.672, 95%CI [0.432, 1.046, p=0.08) . In the meta-analysis of RCTs from Asenjo-Lobos et al., no significant difference between olanzapine and clozapine was found regarding suicide attempts (N=2, n=993, RR=1.67 95%CI [0.40, 6.94]) .

In a meta-analysis including N=6 studies, clozapine was associated with a substantially lower overall risk of suicidal behaviours vs. other treatments (RR=3.3, 95%CI [1.7, 6.3], p<0.0001). The crude pooled RR for suicide rates with/without clozapine was 4.29 and the random-effects RR was 2.90 (95%CI [1.47, 5.72], p=0.002) strongly favouring clozapine. In the meta-analysis from Komossa et al. that meta-analyzed data from the InterSePT trial , a significant difference favouring clozapine vs. olanzapine was reported (N=1, n=980, RR=1.78 95%CI [1.22, 2.62], p<0.01).

***3.2.31 Aggression/hostility in schizophrenia***

In a meta-analysis from Faay et al., clozapine was significantly superior in reducing hostility compared to FGA with a moderate effect size (N=5, n (clozapine)=290, n (haloperidol or CPZ)=247; Hedges’ g=0.415, p<0.0001) . In a Cochrane review on haloperidol for the treatment of long-term aggression, no trials reporting an important decrease in aggression could be identified. For the comparison of haloperidol vs. olanzapine or clozapine, one small trial provided continuous but skewed data that was at high risk of bias and thus, evidence must be considered scarce .

***3.2.32 Comorbid depression in schizophrenia***

In a Cochrane review from Furtado et al., the authors conclude that sulpiride and clozapine might potentially be more beneficial than FGAs for people with both depression and schizophrenia. However, the evidence is both poor and open to bias .

***3.2.33 TRS***

In the meta-analysis from Siskind et al., clozapine response rates in people with TRS were up to 40% . In the meta-analysis from Samara et al. (N=40 blinded RCTs, n=5172), clozapine was more effective than haloperidol (SMD=-0.22, 95%CI [-0.38, -0.07]) and sertindole (SMD=-0.32, 95%CI [-0.63, -0.01]) with regard to overall symptoms. The authors reported that a pattern of superiority could be observed for clozapine (and olanzapine and risperidone) in other efficacy outcomes, but results were not consistent and effect sizes usually small . The authors conclude that little evidence can be provided from blinded – in contrast to unblinded RCTs - for the superiority of clozapine compared with other SGAs. In the meta-analysis from Leucht et al. , clozapine was not more efficacious than olanzapine (N=7, n=570, WMD=-0.2, 95%CI [-3.6, 3.2], risperidone (N=5, n=471, WMD=-1.3, 95%CI [-5.8, 3.2] or ziprasidone (N=1, n=146, WMD=0.5, 95%CI [-6.7, 7.7] in the subanalyses including only TRS trials .

In the meta-analysis from Siskind et al. (N=21, n=1131 on clozapine and n=1233 on control medication including SGAs and FGAs) with mostly double-blind RCTs (N=18) including trials from China, clozapine was superior for positive symptoms in both short-term (SMD=-0.27, 95%CI [-0.47, -0.08]), p=0.006) and long-term studies (SMD=-0.25, 95%CI [-0.43, 0-.07]), p=0.006). With regard to overall and negative symptoms, clozapine was superior in the short-term-studies (SMD=-0.39, 95%CI [-0.61, -0.17]), p=0.0005) . The results were similar to the results from Chakos et al. that indicated superiority of clozapine over FGAs, also in terms of safety with reduced EPS . In another meta-analysis, Mizuno et al. meta-analyzed ten TRS (n=822) and 29 non-TRS (n=2566) studies and showed that clozapine was superior to other APs in improving total symptoms in TRS (g=0.34; 95%CI [0.13, 0.56], p=0.002) . Furthermore, clozapine was superior in improving positive symptoms (g=0.32; 95%CI [0.11, 0.54]; p=0.003), but not for negative symptoms (g=0.22; 95%CI [−0.07, 0.52]; p=0.135) .

**Supplementary Figures:**

**S Fig. 1: Substance abuse as continuous outcome**

Abbrev: k=number of studies, l=long, m=medium, RCT= randomized-controlled trial, S=short.

For continuous outcomes, SMD > 0 means a beneficial outcome for clozapine (e.g. more response or less adverse events.  For dichotomous outcomes, RR > 1 means a beneficial outcome for clozapine (e.g. more response or less adverse events/dropouts).

**S. Fig. 2: Substance abuse as dichotomous outcome**

Abbrev: k=number of studies, l=long, m=medium, RCT= randomized-controlled trial, S=short.

For continuous outcomes, SMD > 0 means a beneficial outcome for clozapine (e.g. more response or less adverse events.  For dichotomous outcomes, RR > 1 means a beneficial outcome for clozapine (e.g. more response or less adverse events/dropouts).

**S. Fig. 3: Other symptoms (cognition, self-injury) as dichotomous outcome**

FGA=first-generation antipsychotic, k=number of studies, L=long-term, M=medium-term, n=number of participants, PANSS=Positive and Negative Syndrome Scale, RCT=randomized-controlled trial, S=short-term, SGA=second-generation antipsychotic, SKT=short cognitive performance test.

For dichotomous outcomes, RR > 1 means a beneficial outcome for clozapine (e.g. more response or less adverse events/dropouts).

**S. Fig. 4: Needing additional medication as dichotomous outcome**

Abbrev: k=number of studies, l=long, m=medium, RCT=randomized-controlled trial, S=short.

For continuous outcomes, SMD > 0 means a beneficial outcome for clozapine (e.g. more response or less adverse events.  For dichotomous outcomes, RR > 1 means a beneficial outcome for clozapine (e.g. more response or less adverse events/dropouts).

**S. Fig. 5: Blood pressure as continuous outcome**

Abbrev: k=number of studies, l=long, m=medium, RCT=randomized-controlled trial, S=short.

For continuous outcomes, SMD > 0 means a beneficial outcome for clozapine (e.g. more response or less adverse events.  For dichotomous outcomes, RR > 1 means a beneficial outcome for clozapine (e.g. more response or less adverse events/dropouts).

**S. Fig. 6: Cognitive side-effects as continuous outcome**

Abbrev: k=number of studies, l=long, m=medium, RCT=randomized-controlled trial, S=short.

For continuous outcomes, SMD > 0 means a beneficial outcome for clozapine (e.g. more response or less adverse events.  For dichotomous outcomes, RR > 1 means a beneficial outcome for clozapine (e.g. more response or less adverse events/dropouts).

**S. Fig. 7: Prolactin levels as continuous outcome**

Abbrev: k=number of studies, l=long, m=medium, RCT=randomized-controlled trial, S=short.

For continuous outcomes, SMD > 0 means a beneficial outcome for clozapine (e.g. more response or less adverse events.  For dichotomous outcomes, RR > 1 means a beneficial outcome for clozapine (e.g. more response or less adverse events/dropouts).

**S. Fig. 8: Motor function as continuous outcome**

Abbrev: k=number of studies, l=long, m=medium, RCT=randomized-controlled trial, S=short, UPDRSM=Unified Parkinson Disease Rating Scale-Motor Section.

For continuous outcomes, SMD > 0 means a beneficial outcome for clozapine (e.g. more response or less adverse events.  For dichotomous outcomes, RR > 1 means a beneficial outcome for clozapine (e.g. more response or less adverse events/dropouts).

**S. Fig. 9: Diabetes as dichotomous outcome**

Abbrev: FGA=first-generation antipsychotics, k=number of studies, l=long, NA=not applicable/not reported in the original publication, RCT=randomized-controlled trial.

For dichotomous outcomes, RR > 1 means a beneficial outcome for clozapine (e.g. more response or less adverse events/dropouts).

**S. Fig. 10: High temperature as dichotomous outcome**

Abbrev: FGA=first-generation antipsychotics, k=number of studies, l=long, m=medium, RCT=randomized-controlled trial, S=short.

For continuous outcomes, SMD > 0 means a beneficial outcome for clozapine (e.g. more response or less adverse events.  For dichotomous outcomes, RR > 1 means a beneficial outcome for clozapine (e.g. more response or less adverse events/dropouts).

**S. Fig. 11: Occupation as dichotomous outcome**

Abbrev: FGA=first-generation antipsychotics, k=number of studies, l=long, m=medium, RCT=randomized-controlled trial, S=short.

For continuous outcomes, SMD > 0 means a beneficial outcome for clozapine (e.g. more response or less adverse events.  For dichotomous outcomes, RR > 1 means a beneficial outcome for clozapine (e.g. more response or less adverse events/dropouts).

**S. Fig. 12: Gastrointestinal symptoms and sign (liver functions, nausea/vomiting) as dichotomous outcome**

Abbrev: k=number of studies, l=long, m=medium, RCT=randomized-controlled trial, S=short.

For continuous outcomes, SMD > 0 means a beneficial outcome for clozapine (e.g. more response or less adverse events.  For dichotomous outcomes, RR > 1 means a beneficial outcome for clozapine (e.g. more response or less adverse events/dropouts).

**S. Fig. 13: Mortality as dichotomous outcome**

Abbrev: FGA=first-generation antipsychotics, k=number of studies, l=long, m=medium, RCT=randomized-controlled trial, S=short,

For continuous outcomes, SMD > 0 means a beneficial outcome for clozapine (e.g. more response or less adverse events.  For dichotomous outcomes, RR > 1 means a beneficial outcome for clozapine (e.g. more response or less adverse events/dropouts).

**S. Fig. 14: Sexual and reproductive side effects as dichotomous outcome**

Abbrev: k=number of studies, l=long, m=medium, RCT=randomized-controlled trial, S=short, SGA=second-generation antipsychotics.

For continuous outcomes, SMD > 0 means a beneficial outcome for clozapine (e.g. more response or less adverse events.  For dichotomous outcomes, RR > 1 means a beneficial outcome for clozapine (e.g. more response or less adverse events/dropouts).

**S. Fig. 15: Glucose abnormalities as dichotomous outcome**

Abbrev: FGA=first-generation antipsychotics, k=number of studies, l=long, m=medium, RCT=randomized-controlled trial, S=short, SGA=second-generation antipsychotics.

For continuous outcomes, SMD > 0 means a beneficial outcome for clozapine (e.g. more response or less adverse events.  For dichotomous outcomes, RR > 1 means a beneficial outcome for clozapine (e.g. more response or less adverse events/dropouts).

**S. Fig. 16: Hospitalization as dichotomous outcome**

Abbrev: FGA=first-generation antipsychotics k=number of studies, l=long, m=medium, RCT=randomized-controlled trial, S=short, SGA=second-generation antipsychotics.

For continuous outcomes, SMD > 0 means a beneficial outcome for clozapine (e.g. more response or less adverse events.  For dichotomous outcomes, RR > 1 means a beneficial outcome for clozapine (e.g. more response or less adverse events/dropouts).

**S. Fig. 17: Suicidality as dichotomous outcome**

Abbrev: k=number of studies, l=long, RCT=randomized-controlled trial.

For continuous outcomes, SMD > 0 means a beneficial outcome for clozapine (e.g. more response or less adverse events.  For dichotomous outcomes, RR > 1 means a beneficial outcome for clozapine (e.g. more response or less adverse events/dropouts).

**S. Fig. 18: Efficacy of treatment for hypersalivation as continuous outcome**

Abbrev: k=number of studies, l=long, m=medium, RCT=randomized-controlled trial, S=short.

For continuous outcomes, SMD > 0 means a beneficial outcome for clozapine (e.g. more response or less adverse events.  For dichotomous outcomes, RR > 1 means a beneficial outcome for clozapine (e.g. more response or less adverse events/dropouts).

**S. Fig. 19: Efficacy of treatment for constipation as dichotomous outcome**

Abbrev: k=number of studies, l=long, m=medium, RCT=randomized-controlled trial, S=short, TCM=traditional chinese medicine.

For continuous outcomes, SMD > 0 means a beneficial outcome for clozapine (e.g. more response or less adverse events.  For dichotomous outcomes, RR > 1 means a beneficial outcome for clozapine (e.g. more response or less adverse events/dropouts).

References

1 Alvares GA, Quintana DS, Hickie IB, Guastella AJ. Autonomic nervous system dysfunction in psychiatric disorders and the impact of psychotropic medications: A systematic review and meta-analysis. Journal of Psychiatry and Neuroscience. 2016;41(2):89-104.

2 Delgado A, Velosa J, Zhang J, Dursun SM, Kapczinski F, de Azevedo Cardoso T. Clozapine in bipolar disorder: A systematic review and meta-analysis. Journal of Psychiatric Research. 2020;125:21-27.

3 Siskind D, Sidhu A, Cross J, Chua YT, Myles N, Cohen D, et al. Systematic review and meta-analysis of rates of clozapine-associated myocarditis and cardiomyopathy. The Australian and New Zealand journal of psychiatry. 2020:4867419898760.

4 Salvo F, Pariente A, Shakir S, Robinson P, Arnaud M, Thomas S, et al. Sudden cardiac and sudden unexpected death related to antipsychotics: A meta-analysis of observational studies. Clinical pharmacology and therapeutics. 2016;99(3):306-14.

5 Lally J, Docherty MJ, MacCabe JH. Pharmacological interventions for clozapine-induced sinus tachycardia. The Cochrane database of systematic reviews. 2016(6):Cd011566.

6 Krause M, Zhu Y, Huhn M, Schneider-Thoma J, Bighelli I, Chaimani A, et al. Efficacy, acceptability, and tolerability of antipsychotics in children and adolescents with schizophrenia: A network meta-analysis. European Neuropsychopharmacology. 2018;28(6):659-74.

7 Kumar A, Datta SS, Wright SD, Furtado VA, Russell PS. Atypical antipsychotics for psychosis in adolescents. The Cochrane database of systematic reviews. 2013(10):Cd009582.

8 Kumra S, Kranzler H, Gerbino-Rosen G, Kester HM, De Thomas C, Kafantaris V, et al. Clozapine and "high-dose" olanzapine in refractory early-onset schizophrenia: a 12-week randomized and double-blind comparison. Biological psychiatry. 2008;63(5):524-9.

9 Cohen D, Bonnot O, Bodeau N, Consoli A, Laurent C. Adverse effects of second-generation antipsychotics in children and adolescents: A bayesian meta-analysis. Journal of Clinical Psychopharmacology. 2012;32(3):309-16.

10 Kennedy E, Kumar A, Datta SS. Antipsychotic medication for childhood-onset schizophrenia. Cochrane Database of Systematic Reviews. 2007;(3) (no pagination)(CD004027).

11 Nielsen RE, Levander S, Kjaersdam Telleus G, Jensen SO, Ostergaard Christensen T, Leucht S. Second-generation antipsychotic effect on cognition in patients with schizophrenia--a meta-analysis of randomized clinical trials. Acta psychiatrica Scandinavica. 2015;131(3):185-96.

12 Woodward ND, Purdon SE, Meltzer HY, Zald DH. A meta-analysis of neuropsychological change to clozapine, olanzapine, quetiapine, and risperidone in schizophrenia. International Journal of Neuropsychopharmacology. 2005;8(3):457-72.

13 Tuunainen A, Wahlbeck K, Gilbody SM. Newer atypical antipsychotic medication versus clozapine for schizophrenia. The Cochrane database of systematic reviews. 2000(2):Cd000966.

14 Asenjo Lobos C, Komossa K, Rummel-Kluge C, Hunger H, Schmid F, Schwarz S, et al. Clozapine versus other atypical antipsychotics for schizophrenia. Cochrane database of systematic reviews (Online). 2010;11:CD006633.

15 Komossa K, Rummel-Kluge C, Hunger H, Schmid F, Schwarz S, Duggan L, et al. Olanzapine versus other atypical antipsychotics for schizophrenia. The Cochrane database of systematic reviews. 2010(3):Cd006654.

16 Essali A, Al-Haj Haasan N, Li C, Rathbone J. Clozapine versus typical neuroleptic medication for schizophrenia. Cochrane Database of Systematic Reviews. 2009;(1) (no pagination)(CD000059).

17 Shirazi A, Stubbs B, Gomez L, Moore S, Gaughran F, Flanagan RJ, et al. Prevalence and predictors of Clozapine-associated constipation: A systematic review and meta-analysis. International Journal of Molecular Sciences. 2016;17 (6) (no pagination)(863).

18 Every-Palmer S, Newton-Howes G, Clarke MJ. Pharmacological treatment for antipsychotic-related constipation. The Cochrane database of systematic reviews. 2017;1:Cd011128.

19 Masuda T, Misawa F, Takase M, Kane JM, Correll CU. Association with Hospitalization and All-Cause Discontinuation among Patients with Schizophrenia on Clozapine vs Other Oral Second-Generation Antipsychotics: A Systematic Review and Meta-analysis of Cohort Studies. JAMA Psychiatry. 2019;76(10):1052-62.

20 Soares-Weiser K, Bechard-Evans L, Lawson AH, Davis J, Ascher-Svanum H. Time to all-cause treatment discontinuation of olanzapine compared to other antipsychotics in the treatment of schizophrenia: a systematic review and meta-analysis. European neuropsychopharmacology : the journal of the European College of Neuropsychopharmacology. 2013;23(2):118-25.

21 Kishimoto T, Hagi K, Nitta M, Kane JM, Correll CU. Long-term effectiveness of oral second-generation antipsychotics in patients with schizophrenia and related disorders: a systematic review and meta-analysis of direct head-to-head comparisons. World Psychiatry. 2019;18(2):208-24.

22 Leucht S, Cipriani A, Spineli L, Mavridis D, Orey D, Richter F, et al. Comparative efficacy and tolerability of 15 antipsychotic drugs in schizophrenia: A multiple-treatments meta-analysis. The Lancet. 2013;382(9896):951-62.

23 Samara MT, Cao H, Helfer B, Davis JM, Leucht S. Chlorpromazine versus every other antipsychotic for schizophrenia: A systematic review and meta-analysis challenging the dogma of equal efficacy of antipsychotic drugs. European Neuropsychopharmacology. 2014;24(7):1046-55.

24 Wahlbeck K, Cheine M, Essali A, Adams C. Evidence of clozapine's effectiveness in schizophrenia: A systematic review and meta-analysis of randomized trials. American Journal of Psychiatry. 1999;156(7):990-99.

25 Huhn M, Nikolakopoulou A, Schneider-Thoma J, Krause M, Samara M, Peter N, et al. Comparative efficacy and tolerability of 32 oral antipsychotics for the acute treatment of adults with multi-episode schizophrenia: a systematic review and network meta-analysis. The Lancet. 2019;394(10202):939-51.

26 Leucht S, Samara M, Heres S, Patel MX, Woods SW, Davis JM. Dose equivalents for second-generation antipsychotics: the minimum effective dose method. Schizophrenia bulletin. 2014;40(2):314-26.

27 Subramanian S, Vollm BA, Huband N. Clozapine dose for schizophrenia. The Cochrane database of systematic reviews. 2017;6:Cd009555.

28 Tsuda Y, Saruwatari J, Yasui-Furukori N. Meta-analysis: The effects of smoking on the disposition of two commonly used antipsychotic agents, olanzapine and clozapine. BMJ Open. 2014;4 (3) (no pagination)(e004216).

29 Leucht S, Komossa K, Rummel-Kluge C, Corves C, Hunger H, Schmid F, et al. A meta-analysis of head-to-head comparisons of second-generation antipsychotics in the treatment of schizophrenia. The American journal of psychiatry. 2009;166(2):152-63.

30 Okhuijsen-Pfeifer C, Sterk AY, Horn IM, Terstappen J, Kahn RS, Luykx JJ. Demographic and clinical features as predictors of clozapine response in patients with schizophrenia spectrum disorders: A systematic review and meta-analysis. Neuroscience and Biobehavioral Reviews. 2020;111:246-52.

31 Leucht S, Arbter D, Engel RR, Kissling W, Davis JM. How effective are second-generation antipsychotic drugs? A meta-analysis of placebo-controlled trials. Molecular Psychiatry. 2009;14(4):429-47.

32 Mizuno Y, McCutcheon RA, Brugger SP, Howes OD. Heterogeneity and efficacy of antipsychotic treatment for schizophrenia with or without treatment resistance: a meta-analysis. Neuropsychopharmacology. 2020;45(4):622-31.

33 Krause M, Huhn M, Schneider-Thoma J, Rothe P, Smith RC, Leucht S. Antipsychotic drugs for elderly patients with schizophrenia: A systematic review and meta-analysis. European neuropsychopharmacology : the journal of the European College of Neuropsychopharmacology. 2018;28(12):1360-70.

34 Leucht S, Wahlbeck K, Hamann J, Kissling W. New generation antipsychotics versus low-potency conventional antipsychotics: A systematic review and meta-analysis. Lancet. 2003;361(9369):1581-89.

35 Rummel-Kluge C, Komossa K, Schwarz S, Hunger H, Schmid F, Kissling W, et al. Second-generation antipsychotic drugs and extrapyramidal side effects: A systematic review and meta-analysis of head-to-head comparisons. Schizophrenia bulletin. 2012;38(1):167-77.

36 Bergman H, Rathbone J, Agarwal V, Soares-Weiser K. Antipsychotic reduction and/or cessation and antipsychotics as specific treatments for tardive dyskinesia. Cochrane Database of Systematic Reviews. 2018;2018 (2) (no pagination)(CD000459).

37 Carbon M, Kane JM, Leucht S, Correll CU. Tardive dyskinesia risk with first- and second-generation antipsychotics in comparative randomized controlled trials: a meta-analysis. World Psychiatry. 2018;17(3):330-40.

38 Zhang JP, Gallego JA, Robinson DG, Malhotra AK, Kane JM, Correll CU. Efficacy and safety of individual second-generation vs. first-generation antipsychotics in first-episode psychosis: A systematic review and meta-analysis. International Journal of Neuropsychopharmacology. 2013;16(6):1205-18.

39 Tek C, Kucukgoncu S, Guloksuz S, Woods SW, Srihari VH, Annamalai A. Antipsychotic-induced weight gain in first-episode psychosis patients: A meta-analysis of differential effects of antipsychotic medications. Early Intervention in Psychiatry. 2016;10(3):193-202.

40 Land R, Siskind D, McArdle P, Kisely S, Winckel K, Hollingworth SA. The impact of clozapine on hospital use: a systematic review and meta-analysis. Acta psychiatrica Scandinavica. 2017;135(4):296-309.

41 Chen SY, Ravindran G, Zhang Q, Kisely S, Siskind D. Treatment Strategies for Clozapine-Induced Sialorrhea: A Systematic Review and Meta-analysis. CNS Drugs. 2019;33(3):225-38.

42 Syed R, Au K, Cahill C, Duggan L, He Y, Udu V, et al. Pharmacological interventions for clozapine-induced hypersalivation. The Cochrane database of systematic reviews. 2008(3):Cd005579.

43 Ayub M, Saeed K, Munshi TA, Naeem F. Clozapine for psychotic disorders in adults with intellectual disabilities. The Cochrane database of systematic reviews. 2015(9):Cd010625.

44 Pillinger T, McCutcheon RA, Vano L, Mizuno Y, Arumuham A, Hindley G, et al. Comparative effects of 18 antipsychotics on metabolic function in patients with schizophrenia, predictors of metabolic dysregulation, and association with psychopathology: a systematic review and network meta-analysis. The Lancet Psychiatry. 2020;7(1):64-77.

45 Vancampfort D, Stubbs B, Mitchell AJ, De Hert M, Wampers M, Ward PB, et al. Risk of metabolic syndrome and its components in people with schizophrenia and related psychotic disorders, bipolar disorder and major depressive disorder: a systematic review and meta-analysis. World Psychiatry. 2015;14(3):339-47.

46 Rummel-Kluge C, Komossa K, Schwarz S, Hunger H, Schmid F, Lobos CA, et al. Head-to-head comparisons of metabolic side effects of second generation antipsychotics in the treatment of schizophrenia: A systematic review and meta-analysis. Schizophrenia Research. 2010;123(2-3):225-33.

47 Siskind DJ, Leung J, Russell AW, Wysoczanski D, Kisely S. Metformin for clozapine associated obesity: A systematic review and meta-analysis. PLoS ONE. 2016;11 (6) (no pagination)(e0156208).

48 Siskind D, Hahn M, Correll CU, Fink-Jensen A, Russell AW, Bak N, et al. Glucagon-like peptide-1 receptor agonists for antipsychotic-associated cardio-metabolic risk factors: A systematic review and individual participant data meta-analysis. Diabetes, Obesity and Metabolism. 2019;21(2):293-302.

49 Srisurapanont M, Suttajit S, Maneeton N, Maneeton B. Efficacy and safety of aripiprazole augmentation of clozapine in schizophrenia: A systematic review and meta-analysis of randomized-controlled trials. Journal of Psychiatric Research. 2015;62:38-47.

50 Zheng W, Xiang YT, Xiang YQ, Li XB, Ungvari GS, Chiu HF, et al. Efficacy and safety of adjunctive topiramate for schizophrenia: a meta-analysis of randomized controlled trials. Acta psychiatrica Scandinavica. 2016;134(5):385-98.

51 Vermeulen JM, Van Rooijen G, Van De Kerkhof MPJ, Sutterland AL, Correll CU, De Haan L. Clozapine and Long-Term Mortality Risk in Patients with Schizophrenia: A Systematic Review and Meta-analysis of Studies Lasting 1.1-12.5 Years. Schizophrenia bulletin. 2019;45(2):315-29.

52 Krause M, Zhu Y, Huhn M, Schneider-Thoma J, Bighelli I, Nikolakopoulou A, et al. Antipsychotic drugs for patients with schizophrenia and predominant or prominent negative symptoms: a systematic review and meta-analysis. European Archives of Psychiatry and Clinical Neuroscience. 2018;268(7):625-39.

53 Myles N, Myles H, Xia S, Large M, Kisely S, Galletly C, et al. Meta-analysis examining the epidemiology of clozapine-associated neutropenia. Acta psychiatrica Scandinavica. 2018;138(2):101-09.

54 Li XH, Zhong XM, Lu L, Zheng W, Wang SB, Rao WW, et al. The prevalence of agranulocytosis and related death in clozapine-treated patients: a comprehensive meta-analysis of observational studies. Psychological medicine. 2020;50(4):583-94.

55 Myles N, Myles H, Xia S, Large M, Bird R, Galletly C, et al. A meta-analysis of controlled studies comparing the association between clozapine and other antipsychotic medications and the development of neutropenia. Australian and New Zealand Journal of Psychiatry. 2019;53(5):403-12.

56 Iketani R, Kawasaki Y, Yamada H. Comparative utility of atypical antipsychotics for the treatment of psychosis in Parkinson's disease: A systematic review and Bayesian network meta-analysis. Biological and Pharmaceutical Bulletin. 2017;40(11):1976-82.

57 Frieling H, Hillemacher T, Ziegenbein M, Neundorfer B, Bleich S. Treating dopamimetic psychosis in Parkinson's disease: Structured review and meta-analysis. European Neuropsychopharmacology. 2007;17(3):165-71.

58 Dzahini O, Singh N, Taylor D, Haddad PM. Antipsychotic drug use and pneumonia: Systematic review and meta-analysis. Journal of Psychopharmacology. 2018;32(11):1167-81.

59 Olagunju AT, Clark SR, Baune BT. Clozapine and Psychosocial Function in Schizophrenia: A Systematic Review and Meta-Analysis. CNS Drugs. 2018;32(11):1011-23.

60 Leucht S, Barnes TRE, Kissling W, Engel RR, Correll C, Kane JM. Relapse prevention in schizophrenia with new-generation antipsychotics: A systematic review and exploratory meta-analysis of randomized, controlled trials. American Journal of Psychiatry. 2003;160(7):1209-22.

61 Kishimoto T, Agarwal V, Kishi T, Leucht S, Kane JM, Correll CU. Relapse prevention in schizophrenia: A systematic review and meta-analysis of second-generation antipsychotics versus first-generation antipsychotics. Molecular Psychiatry. 2013;18(1):53-66.

62 Okhuijsen-Pfeifer C, Huijsman EAH, Hasan A, Sommer IEC, Leucht S, Kahn RS, et al. Clozapine as a first- or second-line treatment in schizophrenia: a systematic review and meta-analysis. Acta psychiatrica Scandinavica. 2018;138(4):281-88.

63 Krause M, Huhn M, Schneider-Thoma J, Bighelli I, Gutsmiedl K, Leucht S. Efficacy, acceptability and tolerability of antipsychotics in patients with schizophrenia and comorbid substance use. A systematic review and meta-analysis. European Neuropsychopharmacology. 2019;29(1):32-45.

64 Temmingh HS, Williams T, Siegfried N, Stein DJ. Risperidone versus other antipsychotics for people with severe mental illness and co-occurring substance misuse. The Cochrane database of systematic reviews. 2018;1:Cd011057.

65 Hennen J, Baldessarini RJ. Suicidal risk during treatment with clozapine: a meta-analysis. Schizophr Res. 2005;73(2-3):139-45.

66 Meltzer HY, Alphs L, Green AI, Altamura AC, Anand R, Bertoldi A, et al. Clozapine treatment for suicidality in schizophrenia: International Suicide Prevention Trial (InterSePT). Archives of general psychiatry. 2003;60(1):82-91.

67 Faay MDM, Czobor P, Sommer IEC. Efficacy of typical and atypical antipsychotic medication on hostility in patients with psychosis-spectrum disorders: a review and meta-analysis. Neuropsychopharmacology. 2018;43(12):2340-49.

68 Khushu A, Powney MJ. Haloperidol for long-term aggression in psychosis. The Cochrane database of systematic reviews. 2016;11:Cd009830.

69 Furtado VA, Srihari V. Atypical antipsychotics for people with both schizophrenia and depression. The Cochrane database of systematic reviews. 2008(1):Cd005377.

70 Siskind D, Siskind V, Kisely S. Clozapine Response Rates among People with Treatment-Resistant Schizophrenia: Data from a Systematic Review and Meta-Analysis. Canadian Journal of Psychiatry. 2017;62(11):772-77.

71 Samara MT, Dold M, Gianatsi M, Nikolakopoulou A, Helfer B, Salanti G, et al. Efficacy, acceptability, and tolerability of antipsychotics in treatment-resistant schizophrenia: A network meta-analysis. JAMA Psychiatry. 2016;73(3):199-210.

72 Siskind D, McCartney L, Goldschlager R, Kisely S. Clozapine v. first- and second-generation antipsychotics in treatment-refractory schizophrenia: systematic review and meta-analysis. The British journal of psychiatry : the journal of mental science. 2016;209(5):385-92.

73 Chakos M, Lieberman J, Hoffman E, Bradford D, Sheitman B. Effectiveness of second-generation antipsychotics in patients with treatment-resistant schizophrenia: A review and meta-analysis of randomized trials. American Journal of Psychiatry. 2001;158(4):518-26.
