# Supplementary material for: Efficacy and safety of clozapine in psychotic disorders—a systematic quantitative meta-review
Source: Transl Psychiatry. 2021 Sep 22;11:487. doi: 10.1038/s41398-021-01613-2 (PMC8458455; doi:10.1038/s41398-021-01613-2)
Supplement: Supplementary file 2 — Supplementary Table 1 [file 41398_2021_1613_MOESM2_ESM.docx]

| **Author (year)** | **Inclusion criteria for study type** | **Specific domain(s) of interest** | | **CLZ-specific MA** | **Population** | **Q1 - Components of PICO** | **Q2 - Protocol** | **Q3 – Selection of study design explained** | **Q4 – Comprehensive literature search** | **Q5 – Study selection** | **Q6 – Data extraction** | **Q7 – List of excluded studies** | **Q8 – Description included studies** | **Q9 – Risk of Bias assessment** | **Q10 – Funding sources** | **Q11 – appropriate statistical methods** | **Q12 – Impact of Risk of Bias on results** | **Q13 – Account for Risk of Bias in Discussion** | **Q14 – Explanation/ Discussion of Heterogeneity** | **Q15 – Publication bias assessment** | **Q16 – Sources of Conflict of interest** | **Overall high quality (yes/no)** |
| --- | --- | --- | --- | --- | --- | --- | --- | --- | --- | --- | --- | --- | --- | --- | --- | --- | --- | --- | --- | --- | --- | --- |
| **Autonomic nervous system dysfunction** | | | | | | | | | | | | | | | | | | | | | | |
| Alvares (2014) | OBS | | Autonomic nervous system dysfunction and psychotropic medication | no | Psychiatric disorders | 1 | 0 | 0 | 0 | 1 | 0 | 0 | 1 | 0,5 | 0 | 1 | 0 | 1 | 0 | 1 | 1 | No |
| **Bipolar disorder (BD)** | | | | | | | | | | | | | | | | | | | | | | |
| Delgado (2020) | RCTs/ OBS | | CLZ for treatment of mania in BD | **yes** | CLZ users with BD | 1 | 0 | 0 | 0,5 | 1 | 1 | 0 | 0,5 | 0 | 0 | 0 | 0 | 1 | 0 | 1 | 1 | No |
| **Cardiological complications** | | | | | | | | | | | | | | | | | | | | | | |
| Salvo (2016) | OBS | | Sudden cardiac/unexpected death | no | AP-users vs non-AP controls | 1 | 0,5 | 0 | 0,5 | 1 | 1 | 1 | 0,5 | 0 | 1 | 0 | 0 | 1 | 1 | 0 | 1 | No |
| Siskind (2020) | RCTs/ OBS | | Myocarditis/cardiomyopathy | **yes** | CLZ users | 1 | 0,5 | 1 | 0,5 | 1 | 1 | 1 | 0,5 | NA/1 | 0 | NA/1 | 1 | 1 | 1 | 1 | 1 | Yes |
| Lally (2016) | RCTs | | Pharmacological interventions for CLZ-induced sinustachycardia | **yes** | CLZ users | 1 | 1 | 1 | 1 | NA | NA | NA | NA | NA | NA | NA | NA | NA | NA | NA | NA | NA |
| **Children and adolescents** | | | | | | | | | | | | | | | | | | | | | | |
| Arango (2019) | RCTs | | Efficacy and tolerability of Lurasidone vs. other SGAs | no | Sz-spectrum | 1 | 0 | 1 | 0 | 0 | 0 | 0 | 0 | 1 | 0 | 1 | 1 | 1 | 1 | 0 | 1 | No |
| Cohen (2012) | RCTs/ OBS | | Adverse effects of SGAs | no | psychiatric disorders | 1 | 0,5 | 0 | 0,5 | 0 | 1 | 0,5 | 0 | 0 | 0 | 0 | 0 | 0 | 0 | 0 | 1 | No |
| Krause (2018) | RCTs | | Efficacy and tolerability of FGAs and SGAs | no | Sz-spectrum | 1 | 1 | 0 | 0,5 | 1 | 1 | 1 | 1 | 1 | 0 | 1 | 1 | 1 | 1 | 1 | 1 | Yes |
| Kumar (2013) | RCTs | | Efficacy of SGAs | no | Sz-spectrum | 1 | 1 | 1 | 1 | 1 | 1 | 1 | 1 | 1 | 1 | 1 | 1 | 1 | 1 | 1 | 1 | Yes |
| Sarkar (2013) | RCTs | | Efficacy and tolerability of FGAs and SGAs | no | Sz-spectrum | 1 | 0 | 0 | 0 | 0 | 1 | 1 | 0 | 0 | 0 | 1 | 0 | 1 | 1 | 0 | 1 | No |
| Pringsheim (2011) | RCTs | | Metabolic and neurological complications of SGAs | no | psychiatric disorders | 1 | 0 | 1 | 0 | 1 | 1 | 0 | 0 | 0 | 0 | 1 | 0 | 0 | 1 | 0 | 1 | No |
| **Childhood-onset Sz** | | | | | | | | | | | | | | | | | | | | | | |
| Kennedy (2007) | RCTs | | Efficacy and tolerability of FGAs and SGAs | no | Sz-spectrum | 1 | 1 | 0 | 0,5 | 1 | 1 | 1 | 1 | 1 | 0 | 1 | 1 | 1 | 1 | 1 | 1 | Yes |
| **Cognition** | | | | | | | | | | | | | | | | | | | | | | |
| Nielsen (2015) | RCTs | | Efficacy of SGAs and FGAs on cognitive domains | no | Sz-spectrum | 1 | 0 | 1 | 0,5 | 0 | 0 | 0 | 0 | 0 | 0 | 1 | 0 | 0 | 1 | 1 | 1 | No |
| Thornton (2006) | RCTs/ OBS | | Efficacy of SGAs and FGAs on long-term memory | no | Sz-spectrum | 1 | 0 | 1 | 0 | 0 | 0 | 0 | 0,5 | 0 | 0 | 1 | 1 | 0 | 1 | 0 | 0 | No |
| Woodward (2005) | RCTs | | Efficacy of SGAs and FGAs on cognitive domains | no | Sz-spectrum | 1 | 0 | 1 | 0 | 0 | 0 | 0 | 0 | 0 | 1 | 1 | 0 | 0 | 1 | 0 | 1 | No |
| **Comorbid depression** | | | | | | | | | | | | | | | | | | | | | | |
| Furtado (2014) | RCTs | | Efficacy of SGAs vs. FGAs/SGAs for Sz + depression | no | Sz-spectrum | 1 | 1 | 1 | 1 | 1 | 1 | 1 | 1 | 1 | 1 | 1 | 1 | 1 | 1 | 1 | 1 | Yes |
| **Comorbid substance abuse** | | | | | | | | | | | | | | | | | | | | | | |
| Krause (2018) | RCTs | | Efficacy and tolerability of APs in Sz + substance abuse | no | Sz-spectrum | 1 | 1 | 1 | 1 | 1 | 1 | 1 | 1 | 1 | 0 | 1 | 1 | 1 | 1 | 1 | 1 | Yes |
| Temmingh (2018) | RCTs | | RIS vs other APs in severe mental illness + substance abuse | no | Psychiatric disorders | 1 | 1 | 1 | 1 | 1 | 1 | 1 | 1 | 1 | 1 | 1 | 1 | 1 | 1 | 1 | 1 | Yes |
| **Constipation and Gastrointestinal Hypomotility** | | | | | | | | | | | | | | | | | | | | | | |
| Every-Palmer (2017) | RCTs | | Pharmacological treatment for AP-related constipation | no | Psychiatric disorders | 1 | 1 | 1 | 1 | 1 | 1 | 1 | 1 | 1 | 1 | 1 | 1 | 1 | 1 | 1 | 1 | Yes |
| Shirazi (2016) | OBS | | Prevalence and predictors of CLZ-associated constipation | **yes** | Sz-spectrum | 1 | 0 | 1 | 0 | 1 | 0 | 0 | 0 | 0 | 0 | 0 | 0 | 0 | 0 | 1 | 1 | No |
| **Discontinuation** | | | | | | | | | | | | | | | | | | | | | | |
| Beasley (2007) | RCTs | | All-cause treatment discontinuation | no | Sz-spectrum | 1 | 0 | 1 | 1 | 0 | 0 | 0 | 0 | 0 | 1 | 1 | 0 | 0 | 1 | 0 | 0 | No |
| Masuda (2019) | OBS | | Hospitalization and all-cause treatment discontinuation, CLZ vs. other oral SGAs | **yes** | Sz-spectrum | 1 | 0 | 1 | 0,5 | 1 | 1 | 0 | 1 | 1 | 0 | 1 | 1 | 1 | 1 | 1 | 1 | Yes |
| Soares-Weiser (2012) | RCTs/ OBS | | Time to all-cause treatment discontinuation | no | Sz-spectrum | 1 | 0 | 1 | 1 | 1 | 0 | 1 | 1 | 1 | 1 | 1 | 1 | 1 | 1 | 1 | 1 | Yes |
| **Dose, dose-response and disposition of CLZ** | | | | | | | | | | | | | | | | | | | | | | |
| Leucht (2014) | RCTs | | Minimum effective dose of SGAs | no | Sz-spectrum | 1 | 0 | 1 | 1 | 0 | 1 | 0 | 0,5 | 0 | 0 | 0 | 0 | 1 | 1 | 0 | 1 | No |
| Subramanian (2017) | RCTs | | Clozapine dose for Sz | **yes** | Sz-spectrum | 1 | 1 | 1 | 1 | 0 | 0 | 1 | 1 | 1 | 1 | 1 | 1 | 1 | 1 | 1 | 1 | Yes |
| Tsuda (2014) | OBS | | Effects of smoking on disposition of OLA and CLZ | **yes** | Psychiatric disorders | 0 | 0 | 0 | 0 | 1 | 0 | 0 | 0,5 | 0 | 0 | 0 | 0 | 0 | 1 | 1 | 1 | No |
| **Efficacy and tolerability for non-first-episode and non-treatment-resistant schizophrenia-spectrum disorders** | | | | | | | | | | | | | | | | | | | | | | |
| Asenjo Lobos (2014) | RCTs | | Efficacy of CLZ vs. other oral SGAs | **yes** | Sz-spectrum | 1 | 1 | 1 | 0,5 | 1 | 1 | 1 | 1 | 1 | 0 | 1 | 1 | 1 | 1 | 1 | 1 | Yes |
| Asmal (2013) | RCTs | | Efficacy and tolerability of QUE vs other oral SGAs | no | Sz-spectrum | 1 | 1 | 1 | 1 | 1 | 1 | 1 | 1 | 1 | 1 | 1 | 1 | 1 | 1 | 1 | 1 | Yes |
| Bai (2016) | RCTs | | Comparative efficacy and tolerability of 8 SGAs | no | acute Sz (Chinese) | 1 | 1 | 1 | 0,5 | 1 | 1 | 0 | 1 | 0 | 0 | 1 | 0 | 0 | 1 | 1 | 1 | No |
| Davis (2003) | RCTs | | Efficacy of SGAs vs FGAs, SGAs vs. SGAs | no | Sz-spectrum | 1 | 0 | 1 | 1 | 0 | 1 | 0 | 0 | 0 | 0 | 1 | 0 | 0 | 1 | 1 | 1 | No |
| Duggan (2005)* | RCTs | | Efficacy and tolerability OLA vs. PLC, FGAs, SGAs | no | Sz-spectrum | 1 | 1 | 1 | 1 | 1 | 1 | 1 | 1 | 1 | 1 | 1 | 1 | 1 | 1 | 1 | 1 | Yes |
| Essali (2009) | RCTs | | Efficacy and tolerability CLZ vs. FGAs (different Sz populations) | **yes** | Sz-spectrum | 1 | O,5 | 1 | 1 | 1 | 1 | 1 | 1 | 1 | 1 | 1 | 1 | 1 | 1 | 1 | 1 | Yes |
| Geddes (2000) | RCTs | | Efficacy and tolerability of SGAs vs. FGAs | no | Sz-spectrum | 1 | 0 | 1 | 0 | 0 | 0 | 0 | 1 | 0,5 | 0 | 1 | 1 | 0 | 1 | 0 | 1 | No |
| Glick (2011) | RCTs/ OBS | | Comparative Mid- and Long-Term Efficacy and Tolerability of SGAs | no | Sz-spectrum | 1 | 0 | 1 | 0 | 0 | 0 | 0 | 0 | 0 | 1 | 0 | 0 | 0 | 0 | 0 | 1 | No |
| Hartling (2012) | RCTs/ OBS | | Comparative efficacy and tolerability of FGAs vs SGAs | no | Sz-spectrum | 1 | 1 | 1 | 0 | 1 | 1 | 0 | 0 | 0,5/0,5 | 1 | 1/0 | 1 | 1 | 1 | 1 | 1 | Yes |
| Khanna (2014) | RCTs | | Comparative efficacy and tolerability of ARI vs SGAs | no | Sz-spectrum | 1 | 1 | 1 | 1 | 1 | 1 | 1 | 1 | 1 | 1 | 1 | 1 | 1 | 1 | 1 | 1 | Yes |
| Kishi  (2017) | RCTs | | Efficacy and tolerability of SGAs, HAL and PLC | no | Sz-spectrum, Japanese | 1 | 0,5 | 1 | 0,5 | 1 | 1 | 0 | 0,5 | 0 | 1 | 0 | 0 | 0 | 1 | 0 | 1 | No |
| Kishimoto (2019) | RCTs | | Long-term effectiveness and tolerability of SGAs vs SGAs | no | Sz-spectrum | 1 | 0 | 1 | 1 | 1 | 1 | 0 | 1 | 0 | 1 | 1 | 1 | 1 | 1 | 1 | 1 | Yes |
| Klemp  (2011) | RCTs | | Efficacy and tolerability of 4 SGAs | no | Sz-spectrum | 1 | 0 | 1 | 0 | 0 | 0 | 0 | 0 | 0 | 0 | 0 | 0 | 0 | 0 | 0 | 1 | No |
| Komossa (2013) | RCTs | | Efficacy and tolerability of OLA vs. other SGAs | no | Sz-spectrum | 1 | 1 | 1 | 1 | 1 | 1 | 1 | 1 | 1 | 1 | 1 | 1 | 1 | 1 | 1 | 1 | Yes |
| Komossa (2014) | RCTs | | Efficacy and tolerability of QUE vs. other SGAs | no | Sz-spectrum | 1 | 1 | 1 | 1 | 1 | 1 | 1 | 1 | 1 | 1 | 1 | 1 | 1 | 1 | 1 | 1 | Yes |
| Komossa (2010) | RCTs | | Efficacy and tolerability of ZOT vs. other SGAs | no | Sz-spectrum | 1 | 1 | 1 | 1 | 1 | 1 | 1 | 1 | 1 | 1 | 1 | 1 | 1 | 1 | 1 | 1 | Yes |
| Komossa (2009) | RCTs | | Efficacy and tolerability of ZIP vs. other SGAs | no | Sz-spectrum | 1 | 1 | 1 | 1 | 1 | 1 | 1 | 1 | 1 | 1 | 1 | 1 | 1 | 1 | 1 | 1 | Yes |
| Komossa (2011) | RCTs | | Efficacy and tolerability of RIS vs. other SGAs | no | Sz-spectrum | 1 | 1 | 1 | 1 | 1 | 1 | 1 | 1 | 1 | 1 | 1 | 1 | 1 | 1 | 1 | 1 | Yes |
| Leucht (2009a) | RCTs | | Efficacy of SGA vs. SGAs | no | Sz-spectrum | 1 | 0 | 1 | 0 | 1 | 1 | 0 | 0 | 1 | 1 | 1 | 1 | 1 | 1 | 1 | 1 | Yes |
| Leucht (2009b) | RCTs | | Efficacy of SGAs vs. PLC | no | Sz-spectrum | 1 | 0 | 1 | 0,5 | 0 | 1 | 0 | 0,5 | 0 | 0 | 1 | 1 | 1 | 1 | 1 | 1 | Yes |
| Leucht (2009c) | RCTs | | Efficacy and tolerability of SGA vs. FGAs | no | Sz-spectrum | 1 | 0 | 1 | 0 | 0 | 1 | 0 | 0 | 1 | 1 | 1 | 1 | 1 | 1 | 1 | 1 | Yes |
| Leucht (2013) | RCTs | | Comparative efficacy and tolerability of 15 AP drugs | no | Sz-spectrum | 1 | 1 | 1 | 1 | 1 | 1 | 0 | 0,5 | 1 | 1 | 1 | 1 | 1 | 1 | 1 | 1 | Yes |
| Okhuijsen-Pfeifer (2020) | OBS | | Demographic and clinical CLZ-response predictors | **yes** | Sz-spectrum | 1 | 0 | 0 | 0,5 | 1 | 0 | 0 | 0 | 0 | 0 | 0 | 0 | 0 | 0 | 1 | 1 | No |
| Samara (2014) | RCTs | | Efficacy of CPZ vs. FGAs/SGAs | no | Sz-spectrum | 1 | 1 | 1 | 0,5 | 0 | 1 | 0 | 1 | 1 | 0 | 1 | 1 | 1 | 1 | 1 | 1 | Yes |
| Sherwood (2012) | RCTs | | Response profile to CLZ | **yes** | Sz | 1 | 0 | 0 | 0 | 1 | 0 | 0 | 0 | 0 | 1 | 0 | 0 | 0 | 0 | 0 | 1 | No |
| Subramanian (2012) | RCTs | | Efficacy of ZOT vs. SGAs | no | Sz-spectrum | 1 | 1 | 1 | 1 | 1 | 1 | 1 | 1 | 1 | 1 | 1 | 1 | 1 | 1 | 1 | 1 | Yes |
| Szegedi (2012) | RCTs | | Efficacy of Asenapine vs. PLC, SGAs | no | acute Sz | 1 | 0 | 1 | 0 | 0 | 0 | 0 | 0 | 0 | 0 | 0 | 0 | 0 | 0 | 0 | 1 | No |
| Tuunainen (2002) | RCTs | | Efficacy of SGAs vs. CLZ | **yes** | Sz-spectrum | 1 | 0 | 1 | 1 | 1 | 1 | 0 | 0,5 | 1 | 0 | 1 | 1 | 1 | 1 | 0 | 0 | Yes |
| Tuunainen (2000) | RCTs | | Efficacy and tolerability of CLZ vs. SGAs | **yes** | Sz-spectrum | 1 | 1 | 1 | 1 | 1 | 1 | 1 | 1 | 1 | 1 | 1 | 1 | 1 | 1 | 1 | 1 | Yes |
| Wahlbeck (1999)** | RCTs | | Efficacy and tolerability of CLZ vs. FGAs | **yes** | Sz-spectrum | 1 | 0 | 1 | 1 | 1 | 1 | 0,5 | 1 | 1 | 1 | 1 | 1 | 1 | 1 | 1 | 1 | Yes |
| **Elderly patients** | | | | | | | | | | | | | | | | | | | | | | |
| Krause (2018) | RCTs | | Efficacy and tolerability of SGAs and FGAs | no | Sz-spectrum | 1 | 1 | 1 | 1 | 1 | 1 | 1 | 1 | 1 | 0 | 1 | 1 | 1 | 1 | 1 | 1 | Yes |
| **Extrapyramidal symptoms (EPS), Tardive dyskinesia (TD)** | | | | | | | | | | | | | | | | | | | | | | |
| Bergman (2018) | RCTs | | Antipsychotic reduction and/or cessation in TD | no | Sz-spectrum | 1 | 1 | 1 | 1 | 1 | 1 | 1 | 1 | 1 | 1 | 1 | 1 | 1 | 1 | 1 | 1 | Yes |
| Carbon (2018) | RCTs | | TD risk with FGAs and SGAs | no | Sz-spectrum | 1 | 0 | 1 | 0,5 | 1 | 1 | 0 | 0 | 0 | 0 | 0 | 0 | 0 | 0 | 0 | 1 | No |
| Leucht (2003) | RCTs | | SGAs vs. FGAs in terms of risk of EPS | no | Sz-spectrum | 1 | 0 | 0 | 1 | 0 | 1 | 0 | 0,5 | 1 | 0 | 1 | 1 | 1 | 1 | 1 | 1 | Yes |
| Mentzel (2018) | RCTs/ OBS | | CLZ-Monotherapy as treatment for TD | **yes** | Sz-spectrum | 1 | 0 | 0 | 0 | 1 | 0 | 0 | 0,5 | 0 | 0 | 1/0 | 0 | 0 | 1 | 1 | 1 | No |
| Rummel-Kluge (2010) | RCTs | | SGAs vs. SGAs in terms of risk for EPS | no | Sz-spectrum | 1 | 0 | 1 | 0,5 | 0 | 1 | 0 | 0 | 1 | 1 | 1 | 1 | 1 | 1 | 1 | 1 | Yes |
| **First-episode schizophrenia-spectrum (FES)** | | | | | | | | | | | | | | | | | | | | | | |
| Tek (2015) | RCTs | | weight gain in FGAs and SGAs vs. PLC | no | Sz-spectrum | 1 | 0 | 1 | 0,5 | 1 | 1 | 0 | 0 | NA | 1 | 0 | 0 | 0 | 0 | 1 | 1 | No |
| Zhang (2013) | RCTs | | Efficacy and tolerability of SGAs vs. FGAs | no | Sz-spectrum | 1 | 0 | 1 | 1 | 0 | 1 | 0 | 1 | 0 | 1 | 1 | 0 | 0 | 1 | 1 | 1 | Yes |
| **Hospitalisation** | | | | | | | | | | | | | | | | | | | | | | |
| Land (2017) | RCTs/ OBS | | Impact of CLZ on hospital use | **yes** | Sz-spectrum | 1 | 0,5 | 1 | 0,5 | 1 | 1 | 0 | 0,5 | 0,5/0,5 | 0 | 1 | 1 | 1 | 1 | 1 | 1 | Yes |
| **Hypersalivation/Sialorrhea** | | | | | | | | | | | | | | | | | | | | | | |
| Chen (2019) | RCTs | | Treatment strategies for CLZ-induced hypersalivation | **yes** | Sz-spectrum | 1 | 0,5 | 1 | 0,5 | 1 | 1 | 1 | 1 | 1 | 0 | 1 | 1 | 1 | 1 | 1 | 1 | Yes |
| Syed (2012) | RCTs | | Treatment of CLZ-induced hypersalivation | **yes** | Sz-spectrum | 1 | 1 | 1 | 1 | 1 | 1 | 1 | 1 | 1 | 0 | 1 | 1 | 1 | 1 | 1 | 1 | Yes |
| **Intellectual disabilities** | | | | | | | | | | | | | | | | | | | | | | |
| Ayub (2015) | RCTs | | CLZ for psychotic disorders + intellectual disabilities | **yes** | Sz-spectrum | 1 | 1 | 1 | 1 | NA | NA | NA | NA | NA | NA | NA | NA | NA | NA | NA | NA | NA |
| **Metabolic complications** | | | | | | | | | | | | | | | | | | | | | | |
| Bak (2014) | RCTs | | Weight gain of FGAs and SGAs | no | Sz-spectrum | 1 | 0 | 1 | 0,5 | 1 | 0 | 0 | 0 | 0 | 0 | 0 | 0 | 0 | 0 | 0 | 1 | No |
| Bartoli (2015a) | OBS | | SGA and adiponectin levels | no | Sz-spectrum | 1 | 0 | 0 | 0,5 | 0 | 1 | 0 | 0,5 | 0 | 0 | 0 | 0 | 1 | 1 | 1 | 1 | No |
| Bartoli (2015b) | OBS | | SGA and plasma adiponectin levels | no | Sz-spectrum | 1 | 0,5 | 0 | 0,5 | 0 | 1 | 0 | 0,5 | 0 | 0 | 0 | 0 | 1 | 1 | 1 | 1 | No |
| Buhagiar (2019) | OBS | | FGAs vs SGAs and lipid abormalities | no | psychiatric disoders | 0 | 0 | 0 | 0 | 1 | 0 | 0 | 0,5 | 0 | 1 | 0 | 0 | 1 | 1 | 0 | 1 | No |
| Correll (2016) | RCTs | | Efficacy and Safety of TOP- Cotreatment for Body Weight | no | Sz-spectrum | 1 | 0 | 1 | 0,5 | 0 | 1 | 0 | 1 | 1 | 0 | 1 | 1 | 1 | 1 | 1 | 1 | Yes |
| Mitchell (2011) | OBS | | Metabolic syndrome and abnormalities | no | Sz-spectrum | 1 | 0 | 0 | 0,5 | 0 | 0 | 0,5 | 0 | 0 | 0 | 0 | 1 | 1 | 1 | 1 | 1 | No |
| Pillinger (2019) | RCTs | | Effects of 18 APs on metabolic outcomes + psychopathology | no | Sz-spectrum | 1 | 1 | 1 | 0,5 | 1 | 1 | 0 | 0,5 | 1 | 0 | 1 | 1 | 1 | 1 | 1 | 1 | Yes |
| Potvin (2015) | RCTs/ OBS | | AP-induced changes in blood levels of leptin | no | Sz-spectrum | 1 | 0 | 0 | 0 | 0 | 1 | 1 | 0 | NA/0 | 0 | NA/0 | 0 | 1 | 0 | 1 | 1 | No |
| Rummel-Kluge (2010) | RCTs | | Metabolic side-effects of SGAs | no | Sz-spectrum | 1 | 0 | 1 | 1 | 0 | 1 | 0 | 1 | 1 | 1 | 1 | 0 | 0 | 1 | 0 | 1 | Yes |
| Siskind (2016) | RCTs | | Metformin vs. PLC for CLZ-associated obesity | **yes** | Sz-spectrum | 1 | 0,5 | 1 | 0,5 | 1 | 1 | 0 | 1 | 1 | 1 | 1 | 1 | 1 | 1 | 1 | 1 | Yes |
| Siskind (2018) | RCTs | | GLP-1 Receptor agonists for AP-associated cardiometabolic risk factors | no | Sz-spectrum | 1 | 0,5 | 0 | 1 | 1 | 0 | 1 | 1 | 1 | 0 | 1 | 1 | 1 | 1 | 1 | 1 | Yes |
| Smith (2008) | RCTs/ OBS | | FGAs vs. SGAs and risk for diabetes | no | Sz-spectrum | 1 | 0 | 0 | 1 | 0 | 0 | 0 | 0,5 | NA/0,5 | 1 | NA/1 | 1 | 1 | 1 | 0 | 1 | No |
| Srisurapanont (2015) | RCTs | | Efficacy and safety of CLZ + ARI for cardiometabolic risk reduction | **yes** | Sz-spectrum | 1 | 0 | 0 | 1 | 0 | 1 | 0 | 0,5 | 1 | 1 | 1 | 1 | 1 | 1 | 0 | 1 | Yes |
| Vancampfort (2015) | RCT/OBS | | Risk of MetS | no | Psychiatric disorders | 1 | 0 | 1 | 0,5 | 1 | 0 | 1 | 0 | 0/1 | 0 | NA/0 | 1 | 1 | 1 | 1 | 1 | Yes |
| Zhang (2017) | RCTs | | Metabolic side-effects on glucose of 12 APs | no | Sz-spectrum | 1 | 0 | 1 | 0 | 1 | 1 | 0 | 0,5 | 1 | 0 | 0 | 1 | 0 | 0 | 1 | 1 | No |
| Zheng (2016) | RCTs | | Efficacy and safety of adjunctive TOP for weight reduction | no | Sz-spectrum | 1 | 0,5 | 1 | 0,5 | 1 | 1 | 0 | 1 | 1 | 0 | 1 | 1 | 1 | 1 | 1 | 1 | Yes |
| Zimbron (2016) | RCTs | | Treatment strategies for CLZ-induced obesity and MetS | **yes** | Sz-spectrum | 1 | 0 | 1 | 0,5 | 1 | 1 | 1 | 1 | NA | 0 | 1 | 0 | 1 | 1 | 0 | 1 | No |
| **Mortality** | | | | | | | | | | | | | | | | | | | | | | |
| Vermeulen (2019) | 1 RCT/OBS | | CLZ and Long-Term Mortality Risk | **yes** | Sz-spectrum | 1 | 0,5 | 1 | 0,5 | 1 | 1 | 0 | 1 | 1/1 | 0 | NA/1 | 1 | 1 | 1 | 1 | 1 | No |
| **Multi-episode Sz (MES)** | | | | | | | | | | | | | | | | | | | | | | |
| Huhn (2019) | RCTs | | Comparative efficacy and tolerability of 32 oral APs for acute treatment | no | Acute MES | 1 | 1 | 1 | 1 | 1 | 1 | 0 | 1 | 1 | 1 | 1 | 1 | 1 | 1 | 1 | 1 | Yes |
| **Negative symptoms** | | | | | | | | | | | | | | | | | | | | | | |
| Krause (2018) | RCTs | | AP for predominant negative symptoms | no | Sz-spectrum | 1 | 1 | 1 | 1 | 1 | 1 | 1 | 0,5 | 1 | 1 | 1 | 1 | 1 | 1 | 1 | 1 | Yes |
| **Neutropenia** | | | | | | | | | | | | | | | | | | | | | | |
| Li (2019) | OBS | | Prevalence of agranulocytosis and related death in CLZ-treated patients | **yes** | Sz-spectrum | 1 | 1 | 1 | 1 | 1 | 1 | 0.5 | 1 | 1 | 0 | 1 | 1 | 1 | 1 | 1 | 1 | Yes |
| Myles (2018) | RCTs/ OBS | | Epidemiology of CLZ-associated neutropenia | **yes** | Sz-spectrum | 1 | 0,5 | 1 | 0,5 | 1 | 1 | 0 | 0 | 0/0 | 0 | 0/1 | 1 | 1 | 1 | 1 | 1 | Yes |
| Myles (2019) | RCTs/ OBS | | Association between CLZ and other APs and neutropenia risk | **yes** | Sz-spectrum | 1 | 0,5 | 1 | 1 | 1 | 1 | 0 | 0,5 | 0/0,5 | 0 | 1/1 | 0 | 1 | 1 | 1 | 1 | Yes |
| **Parkinson’s disease psychosis (PDP) and drug-induced psychosis (DIP) in PD** | | | | | | | | | | | | | | | | | | | | | | |
| Frieling (2007) | RCTs | | Efficacy of treatment strategies for DIP PD | no | PD with DIP | 1 | 0 | 1 | 0,5 | 1 | 1 | 1 | 0,5 | 0,5 | 0 | 1 | 0 | 1 | 1 | 0 | 0 | No |
| Iketani (2017) | RCTs | | Comparative utility of SGAs for treatment of PDP | no | PDP | 1 | 0 | 0 | 0,5 | 1 | 1 | 0 | 0,5 | 1 | 0 | 0 | 1 | 0 | 0 | 0 | 1 | No |
| Jethwa (2015) | RCTs | | Efficacy of APs in treatment of PDP | no | PDP | 1 | 0 | 0 | 0 | 0 | 0 | 0 | 0,5 | 1 | 0 | 0 | 0 | 1 | 0 | 0 | 1 | No |
| Zhang (2019) | RCTs | | Efficacy of SGAs for PDP | no | PDP | 0 | 0 | 0 | 0,5 | 0 | 1 | 0 | 0 | 1 | 0 | 1 | 0 | 0 | 0 | 0 | 1 | No |
| **Pneumonia** | | | | | | | | | | | | | | | | | | | | | | |
| Dzahini (2018) | OBS | | FGAs and SGAs and risk for pneumonia | no | Psychiatric disorders | 1 | 0 | 1 | 0 | 1 | 0 | 0 | 0,5 | 0,5 | 0 | 0 | 0 | 1 | 0 | 1 | 1 | No |
| **Psychosocial function** | | | | | | | | | | | | | | | | | | | | | | |
| Olagunju (2018) | RCTs | | CLZ and psychosocial function | **yes** | Sz-spectrum | 1 | 0 | 1 | 1 | 0 | 1 | 0 | 1 | 1 | 1 | 1 | 1 | 1 | 1 | 1 | 1 | Yes |
| **Relapse prevention** | | | | | | | | | | | | | | | | | | | | | | |
| Kishimoto (2013) | RCTs | | Relapse prevention of SGAs vs. FGAs | no | Sz-spectrum | 1 | 0 | 1 | 1 | 0 | 1 | 0 | 0 | 1 | 1 | 1 | 1 | 1 | 1 | 1 | 1 | Yes |
| Leucht (2003) | RCTs | | Relapse prevention of SGAs | no | Sz-spectrum | 1 | 0,5 | 1 | 1 | 0 | 0 | 1 | 1 | 0 | 1 | 1 | 1 | 1 | 1 | 1 | 1 | Yes |
| **Second-line treatment** | | | | | | | | | | | | | | | | | | | | | | |
| Cheine (1998) | RCTs | | Pharmacological treatment of Sz resistant to first-line treatment | no | Sz-spectrum | 1 | 0 | 0 | 0,5 | 0 | 0 | 0 | 0 | 0 | 0 | 0 | 0 | 0 | 0 | 0 | 0 | No |
| Okhuijsen-Pfeifer (2018) | RCTs/ OBS | | CLZ as first- or second-line treatment in Sz | **yes** | Sz-spectrum | 1 | 0 | 1 | 0 | 1 | 1 | 0 | 0 | 0,5/0 | 1 | 1/1 | 1 | 1 | 1 | 1 | 1 | Yes |
| **Sexual dysfunction (SD)** | | | | | | | | | | | | | | | | | | | | | | |
| Serretti (2011) | RCTs/OBS | | Association of sexual dysfunction and intake of APs | no | Psychiatric disorders | 1 | 0 | 1 | 0 | 0 | 0 | 0 | 0,5 | 0/0 | 0 | 0/0 | 0 | 0 | 0 | 0 | 0 | No |
| **Suicidality and hostility/aggression vs. others** | | | | | | | | | | | | | | | | | | | | | | |
| Faay (2018) | RCTs/ OBS | | Efficacy of FGAs and SGAs on hostility | no | Sz-spectrum | 1 | 0 | 0 | 0,5 | 0 | 1 | 0 | 0,5 | 0/0 | 1 | 1/0 | 0 | 0 | 1 | 0 | 1 | No |
| Hennen (2004) | RCTs/ OBS | | Efficacy of CLZ on suicidal risk | **yes** | Sz-spectrum | 0 | 0 | 0 | 0 | 0 | 0 | 0 | 0 | 0/0 | 0 | 0/0 | 0 | 0 | 0 | 0 | 0 | No |
| Khushu (2016) | RCTs | | Efficacy of HAL vs. other APs for long-term aggression | no | Sz-spectrum | 1 | 1 | 1 | 1 | 1 | 0 | 1 | 1 | 1 | 1 | 1 | 1 | 1 | 1 | 1 | 1 | Yes |
| **Treatment-resistant schizophrenia-spectrum (TRS)** | | | | | | | | | | | | | | | | | | | | | | |
| Chakos (2001) | RCTs | | Efficacy of SGAs | no | TRS | 1 | 0 | 1 | 0 | 0 | 0 | 0 | 0,5 | 0 | 0 | 0 | 0 | 0 | 0 | 0 | 0 | No |
| Mizuno (2019) | RCTs | | Efficacy of APs for Sz with or without TRS | no | Sz-spectrum | 1 | 0,5 | 1 | 1 | 1 | 1 | 0 | 0,5 | 1 | 1 | 1 | 1 | 1 | 1 | 1 | 1 | Yes |
| Moncrieff (2003) | RCTs | | Efficacy of CLZ vs. FGAs | **yes** | TRS | 0 | 0 | 0 | 0,5 | 0 | 0 | 0 | 0 | 0 | 0 | 0 | 0 | 0 | 0 | 0 | 1 | No |
| Samara (2016) | RCTs | | Efficacy and tolerability of APs | no | TRS | 1 | 1 | 1 | 1 | 1 | 1 | 0 | 1 | 1 | 1 | 1 | 1 | 1 | 1 | 1 | 1 | Yes |
| Siskind (2016) | RCTs | | Efficacy of CLZ vs. FGAs and SGAs | **yes** | TRS | 1 | 0,5 | 1 | 1 | 1 | 1 | 1 | 1 | 1 | 1 | 1 | 1 | 1 | 1 | 1 | 1 | Yes |
| Siskind (2017) | RCTs | | Response rates | **yes** | TRS | 1 | 0,5 | 1 | 1 | 1 | 1 | 0 | 1 | 1 | 1 | 0 | 1 | 1 | 1 | 1 | 1 | Yes |
| Souza (2013) | RCTs | | Efficacy of OLA vs CLZ | **yes** | TRS | 1 | 0 | 0 | 0,5 | 1 | 0 | 0 | 0 | 0,5 | 1 | 1 | 0 | 1 | 1 | 1 | 1 | No |

***Supplementary Table 1: AMSTAR-2 Ratings of included meta-analyses.***

***Abbreviations:*** 1=yes, 0.5=partial yes, 0=no;

AP=antipsychotic, ARI=Aripiprazole, CLZ=clozapine, CPZ=chlorpromazine, DIP=drug-induced psychosis, FGA=first-generation antipsychotic, HAL=Haloperidol, OBS=observational study, OLA=olanzapine, PD=Parkinson’s disease, PDP=Parkinson’s disease psychosis, PLC=placebo, RCT=randomized controlled trial, SGA=second-generation antipsychotic, SZ=schizophrenia, TD=tardive dyskinesia, TOP=topiramate, TRS=treatment-resistant schizophrenia, NA=not applicable due to incomplete nature of meta-analysis.

*meta-analysis Duggan et al., 2005 was published as third update (after 2000 and 2003) in *Cochrane Database of systematic reviews* and thus only newest update (2005) was reviewed.

**meta-analysis published as Wahlbeck et al., “Evidence of Clozapine’s effectiveness in Schizophrenia: A Systematic Review and Meta-Analysis of Randomized Trials” Am J Psychiatry, 1999 and as Cochrane review published in The Cochrane Library 1999, Issue 4. Only first article was reviewed.
