# Supplementary material for: Efficacy and safety of clozapine in psychotic disorders—a systematic quantitative meta-review
Source: Transl Psychiatry. 2021 Sep 22;11:487. doi: 10.1038/s41398-021-01613-2 (PMC8458455; doi:10.1038/s41398-021-01613-2)
Supplement: Supplementary file 3 — Supplementary Table 2 [file 41398_2021_1613_MOESM3_ESM.docx]

| ***Domain*** | ***Meta-analysis*** | ***High-quality in AMSTAR-2*** | ***EP for clozapine analyses*** | ***Description of results*** |
| --- | --- | --- | --- | --- |
| Autonomic nervous system dysfunction | Alvares (2014) | No | Heart rate variability  (HRV) | “Clozapine use had a significant detrimental effect on HRV (Hedges g = –0.643, 95%CI -0.973 to -0.313, p<0.001).” |
| Bipolar disorder | Delgado (2020) | No | BRMS | “Clozapine’s efficacy was similar to other antipsychotics: (MD=0.03, 95%CI -0.86 to 0.92, p=0.59) in manic episodes.” |
| Cardiological outcomes | Salvo (2016) | No | SCD/SUD | “Compared with nonusers, the risk was increased for clozapine (OR=3.67, 1.94–6.94)”. |
| Cardiological outcomes | Siskind (2020) | Yes | Event rates of myocarditis | “The event rate of myocarditis was 0.007 (95%CI 0.003 to 0.016), the cardiomyopathy event rate was 0.006 (95%CI 0.002 to 0.023).” |
| Cardiological outcomes | Lally (2016) | No | Change in heart rate | No meta-analysis conducted due to lack of data. |
| Children/adolescents | Arango (2019) | No | Weight gain in relation to lurasidone in children/adolescents | Lurasidone vs. clozapine: no data for CGI, PANSS, for change of weight gain: MD of−3.81 kg (95%CI −8.03 to 0.42) in advantage of lurasidone. |
| Children/adolescents | Cohen (2012) | No | Weight gain children/adolescents | “Compared with placebo, significant treatment-related increases were observed for weight gain with clozapine (2.38 +/- 1.13 kg; 95%CI 0.19 to 4.62 kg)” |
| Children/adolescents | Krause (2018) | Yes | PANSS, BPRS in children/adolescents | “Clozapine was significantly more efficacious than all the other antipsychotics with SMDs between -0.71 (95%CI -1.38, -0.05) compared to olanzapine and -1.15 (95%CI -1.92 to -0.39) in comparison with ziprasidone for negative symptoms, and SMDs between 1.13 (95%CI 0.33 to 1.93) compared to molindone and 1.72 (95%CI 0.97 to 2.48) compared to placebo for positive symptoms.” |
| Children/adolescents | Kumar (2013) | Yes | CGAS scores in children/adolescents | “In the study from Kumra et al. 1996, the mean end point CGAS score clearly favoured young people treated with clozapine (1 RCT, n = 21, RR 17.00, 95% CI 7.74 to 26.26) compared with haloperidol.” |
| Children/adolescents | Sarkar (2013) | No | PANSS, BPRS, CGI in children/adolescents | “Clozapine was superior to all other antipsychotics (ES=0.848, 95%CI 0.748 to 0.948, n=85) in the treatment of schizophrenia in children and adolescents.” |
| Children/adolescents | Pringsheim (2011) | No | Variety of metabolic parameters in children/adolescents | “Mean weight gain with clozapine was 3.8 – 6.0 kg, while the mean weight gain with olanzapine was 3.6 – 4.0 kg (p=0.96). Change in BMI was 1.6 – 2.5 kg/m^2^ with clozapine and 1.4 – 1.6 kg/m^2^ with olanzapine (p=0.76).” |
| Childhood-onset Sz | Kennedy (2007) | Yes | Overall change in symptoms in childhood-onset Sz | “A few results from one study favoured the atypical antipsychotic clozapine over haloperidol in treating treatment resistant childhood-onset schizophrenia (n=21, WMD CGAS 17.00 CI 7.74 to 26.26; n=21, WMD Bunney-Hamburg Psychosis Rating Scale -3.60 CI -6.64 to -0.56). Participants on clozapine, however, were three times more likely to have drowsiness (1 RCT, n=21, RR=3.30 CI 1.23 to 8.85, 95%CI 2 to 17) and half of the children receiving clozapine had neutropenia (1 RCT, n=21, RR=12, 95%CI 0.75 to 192.86).” |
| Cognition in Sz | Nielsen (2015) | No | Cognitive composite scores and each cognitive domain | “On cognitive composite score, sertindole was superior to clozapine, ES 0.87; 95% CI 0.12 to 1.63, quetiapine, ES 0.75; 95%CI 0.00 to 1.49, and first-generation antipsychotics (FGAs), ES 0.89; 95%CI 0.14 to 1.64.” |
| Cognition in Sz | Thornton (2006) | No | Change in long-term memory (LTM) | “Clozapine trials did not produce a LTM advantage over typical trials (ES=-0.06; 95%CI -0.35 to 0.23).” |
| Cognition in Sz | Woodward (2005) | No | Change in cognitive domains (e.g. Verbal Fluency) | After excluding the uncontrolled studies, the Verbal Fluency effect size for clozapine (ES=0.41, Z=2.87, p<0.005) remained significant in comparison vs. other atypical antipsychotics. |
| Sz and comorbid depression | Furtado (2014) | Yes | Mental state | “When clozapine was compared with any other antipsychotic drug plus an antidepressant or placebo, clozapine constantly scored better on Hamilton scores (1 RCT, n=29, WMD vs antipsychotic + mianserin −5.53 CI −8.23 to −2.8; 1 RCT, n=32, WMD vs antipsychotic + moclobemide =−4.35, 95%CI −6.7 to −2.03; 1 RCT, n=33, WMD vs antipsychotic + placebo −6.35 95%CI −8.6 to −4.1).” |
| Sz and comorbid substance abuse | Krause (2018) | Yes | Reduction of substance use | “Clozapine was superior to “any other antipsychotic” for reduction of substance use (n=31, SMD -1.08, 95%CI -1.84 to -0.32).” |
| Sz and comorbid substance abuse | Temmingh (2018) | Yes | Effects of risperidone compared to other APs (FGAs and SGAs) in people with serious mental illness and substance misuse | “For risperidone versus clozapine, no clear differences between these two antipsychotics in the reduction of positive psychotic symptoms (1 RCT, n = 36, MD 0.90, 95%CI −2.21 to 4.01, very low quality evidence), or reduction in cannabis use (1 RCT, n = 14, RR 1.00, 95% CI 0.30 to 3.35, very low quality evidence), improvement in subjective well-being (1 RCT, n = 36, MD −6.00, 95% CI −14.82 to 2.82, very low quality evidence), numbers discontinuing medication (1 RCT, n = 36, RR 4.05, 95% CI 0.21 to 78.76, very low quality evidence) extrapyramidal side-effects (2 RCTs, n = 50, RR 2.71, 95% CI 0.30 to 24.08; IÇ = 0%, very low quality evidence), or leaving the study early (2 RCTs, n = 45, RR 0.49, 95% CI 0.10 to 2.51; I^2^ = 34%, very low quality evidence) were found. Clozapine was associated with lower levels of craving for cannabis (1 RCT, n = 28, MD 7.00, 95% CI 2.37 to 11.63, very low quality evidence).” |
| Constipation | Every-Palmer (2017) | Yes | Change in constipation among patients treated with CLZ and other APs | Very low-quality evidence from a total of two identified studies. |
| Constipation | Shirazi (2016) | No | effectiveness and safety of pharmacologic treatment (versus placebo or compared against another treatment) for antipsychotic-related constipation | “From 32 studies, a pooled prevalence of clozapine-associated constipation of 31.2% (95%CI 25.6 to 37.4) (n = 2013) was estimated. People taking clozapine were significantly more likely to be constipated versus other antipsychotics (OR=3.02 (CI 1.91 to 4.77), p<0.001, n=11 studies).” |
| Treatment discontinuation | Beasley (2007) | No | Rate of treatment discontinuation relative to olanzapine | “No significantly (p<0.05) greater likelihood of discontinuation relative to olanzapine treatment (hazard ratio was observed for clozapine (1.2 95%CI 0.9 to 1.6).” |
| Treatment discontinuation | Masuda (2019) | Yes | Hospitalisation and ACD rate between clozapine and non-clozapine studies | “Compared with non-clozapine-SGAs, despite greater illness severity (17 studies [n=38766]; Hedges g, 0.222; 95%CI 0.013 to 0.430; p=0.04), clozapine was significantly associated with lower hospitalization risk (19 studies [n=49 453]; RR=0.817; 95%CI 0.725 to 0.920; p=0.001; 95%CI 12 to 40) and ACD (16 studies (n=56 368); RR=0.732; 95%CI 0.639 to 0.838; p<0.001; 95%CI 6 to 12). Clozapine was also significantly associated with better outcomes regarding overall symptoms (SMD=−0.302; 95%CI −0.572 to −0.032; p=0.03). Clozapine was significantly associated with increases in body weight (MD=1.70; 95%CI 0.31 to 3.08 kg; p=0.02), BMI (MD=0.96; 95%CI 0.24 to 1.68; p=0.009), and type 2 diabetes (RR=1.777; 95%CI 1.229 to 2.570; p=0.002; 95%CI 13 to 90).” |
| Treatment discontinuation | Soares-Weiser (2012) | Yes | Time-to-discontinuation in relation to olanzapine | “No significant difference was observed between olanzapine and clozapine (HR=1.37, 95%CI 0.99 to 1.89, 5 studies, 14222 participants).” |
| Dose-response | Leucht (2014) | No | minimum effective dose= a dose had to  be significantly more efficacious than placebo in the primary  outcome of at least one RCTS (fixed-dose) in outcomes PANSS/BPRS | The minimum effective daily doses/olanzapine equivalents based were: clozapine 300 mg/40. |
| Dose-response | Subramanian (2017) | Yes | Mental state | “No evidence of effect on mental state between clozapine standard, low and very low dose regimes, but no identification of any trials on high or very high doses of clozapine.” |
| Disposition in smokers and non-smokers | Tsuda (2014) | No | C/D ratio | “Four association studies of clozapine were included in the meta-analysis of clozapine, comprising 196 patients (120 smokers and 76 nonsmokers) with schizophrenia or other psychiatric disorders. The C/D ratio was significantly lower in smokers than in non-smokers (p<0.00001), and the MD was −1.11 (ng/mL)/(mg/day) (95%CI −1.53 to −0.70).” |
| SZ | Asenjo Lobos (2014) | Yes | Effects of clozapine with other SGAs | “Clozapine had a higher attrition rate due to adverse effects than olanzapine (9 RCTs, n=1674, RR 1.60 CI 1.07 to 2.40, CI 15 to 73) and risperidone (6 RCTs, n=627, RR=1.88, CI 1.11 to 3.21). Fewer participants in the clozapine groups left the trials early due to inefficacy than risperidone (6 RCTs, n=627, RR 0.40 CI 0.23 to 0.70, NNT 11 CI 7 to 21), suggesting a certain higher efficacy of clozapine. Clozapine was more efficacious than zotepine in improving the participants general mental state (BPRS total score: 1 RCT, n=59, MD −6.00 CI −9.83 to −2.17), but not consistently more than olanzapine, quetiapine, risperidone and ziprasidone. No significant difference between clozapine and olanzapine or risperidone in terms of positive or negative symptoms.” |
| SZ | Asmal (2013) | Yes | mental state, quetiapine vs. other APs, | “No clear mental state differences were noted when quetiapine was compared with clozapine.” |
| SZ | Bai (2016) |  | PANSS (Chinese population) | No significant difference was found between risperidone, ziprasidone, clozapine, quetiapine and aripiprazole with regard to overall symptoms. |
| SZ | Davis (2003) |  | PANSS, BRPS | “For overall symptoms, the effect size of clozapine was 0.49 greater than those of FGAs, p=2x10^-8^.” |
| SZ | Duggan (2005)* | Yes | Mental state in comparison to olanzapine | “No important clinical response as measured by CGI (n=180, RR 0.82 CI 0.6-1.1) or by Kane 1988 criteria (n=180, RR 0.93 CI 0.8-1.2) when olanzapine was compared to clozapine.” |
| SZ | Essali (2009) | Yes | Mental state | “No significant difference in the effects of clozapine and typical neuroleptic drugs for broad outcomes such as mortality, ability to work or suitability for discharge at the end of the study. Clinical improvements were seen more frequently in those taking clozapine (n=1119, 14 RCTs, RR=0.72 CI 0.7 to 0.8, NNT 6, CI 5 to 8). Also, participants given clozapine had fewer relapses than those on typical antipsychotic drugs (n=1303, RR=0.62 CI 0.5 to 0.8, NNT 21 CI 15 to 49). BPRS scores showed a greater reduction of symptoms in clozapine-treated participants, (n=1205, 17 RCTs, WMD=-3.79 CI -4.9 to -2.7). Short-term data from the SANS negative symptom scores favored clozapine (n=196, 6 RCTs, WMD=-7.21 CI -8.9 to -5.6). Clozapine was found to be more acceptable in long-term treatment than conventional antipsychotic drugs (n=982, 6 RCTs, RR=0.60 CI 0.5 to 0.7, NNT 15 CI 12 to 20). Blood problems occurred more frequently in participants receiving clozapine (3.2%) compared with those given typical antipsychotic drugs (0%) (n=1031, 13 RCTs, RR=7.09 CI 2.0 to 25.6). Clozapine participants experienced more drowsiness, hypersalivation or temperature increase, than those given conventional neuroleptics. However, those receiving clozapine experienced fewer motor adverse effects (n=1495, 19 RCTs, RR 0.57 CI 0.5 to 0.7, NNT 5, CI 4 to 6). The clinical effects of clozapine were more pronounced in participants resistant to typical neuroleptics in terms of clinical improvement (n=370, 4 RCTs, RR=0.71 CI 0.6 to 0.8, NNT 4, CI 3 to 6) and symptom reduction. Thirty-four per cent of treatment-resistant participants had a clinical improvement with clozapine treatment.” |
| SZ | Geddes (2000) | No | Overall outcome | “The SMD for the overall symptom score in short term trials was -0.68 (-0.82 to -0.55) in favor of clozapine, signifying that about 75% of patients given a conventional antipsychotic had higher symptom scores after treatment than the average patient treated with clozapine. Patients allocated to clozapine were less likely to drop out (OR=0.52 (CI 0.40 to 0.67) with fixed effects model), although the OR became non-significant with the random effects model (0.69, CI 0.45 to 1.19).” |
| SZ | Glick  (2011) | No | For clozapine only meta-analytic data on weight gain available | Weight gain was greater with clozapine and olanzapine, RR for weight gain clozapine vs. olanzapine not statistically significant.” |
| SZ | Hartling (2012) | Yes | Overall outcome | “Low-strength evidence showed no difference in mortality for chlorpromazine versus clozapine and higher incidence of tardive dyskinesia for chlorpromazine versus clozapine (risk differences, 5% and 9%).” |
| SZ | Khanna (2014) | Yes | Mental state in relation to aripiprazole | “Global state - No clinically significant response (as defined by original studies): Data demonstrate there was no difference between groups (29 RCTs, n=2132, RR=1.05, CI 0.87 to 1.27).” |
| SZ | Kishi  (2017) | No | Response rate, ACD | In this Bayesian NMA (patients on clozapine=47), clozapine was the most effective antipsychotic, although not significantly superior to placebo. |
| SZ | Kishimoto (2019) | Yes | ACD | “Clozapine had a significantly lower ACD as compared with quetiapine (one study, n=64, RR=0.59, 95% CI: 0.42 to 0.83, p=0.002) and risperidone (four studies, N=216, RR=0.74, 95%CI 0.57 to 0.95, p=0.020).” |
| SZ | Klemp (2011) | No | Response ratio | “All SGAs analyzed are fairly effective with response ratios against placebo ranging between 1.55 (CI 1.36 to 1.76) and 1.99 (CI 1.76 to 2.26), with clozapine being the most effective and aripiprazole the least effective among them.” |
| SZ | Komossa (2013) | Yes | Mental state in relation to olanzapine | “Olanzapine improved the general mental state (PANSS total score) not more than clozapine.” |
| SZ | Komossa (2014) | Yes | Mental state in relation to quetiapine | “There were no clear mental state differences when quetiapine was compared with clozapine.” |
| SZ | Komossa (2010) | Yes | Mental state in relation to zotepine | “Zotepine was less effective than clozapine (no clinically significant response: n=59, 1 RCT, RR=8.23 CI 1.14 to 59.17; average score (BPRS total) at endpoint (n=59, 1 RCT, MD 6.00 CI 2.17 to 9.83).” |
| SZ | Komossa (2009) | Yes | Mental state in relation to ziprasidone | “Based on limited data there were no significant differences in tolerability between ziprasidone and clozapine.” |
| SZ | Komossa (2011) | Yes | Mental state in relation to risperidone | “Risperidone improved the general mental state (PANSS total score) slightly less than olanzapine (15 RCTs, n=2390, MD 1.94 CI 0.58 to 3.31), but slightly more than quetiapine (9 RCTs, n=1953, MD=-3.09 CI -5.16 to -1.01) and ziprasidone (3 RCTs, n=1016, MD=-3.91, CI -7.55 to -0.27). The comparisons with the other SGA drugs were equivocal.” |
| SZ | Leucht (2009a) | Yes | Overall efficacy | “Clozapine was not significantly different from olanzapine (n=619), quetiapine (n=232), risperidone(n=466), and ziprasidone (n=146). Clozapine was significantly more efficacious than zotepine (n=59, WMD=–6.0, p=0.002).” |
| SZ | Leucht (2009b) | Yes | Overall efficacy | “Nevertheless, with the exception of clozapine (SMD=–1.65, based on only one study), the ESs were moderate (pooled ES of all SGA drugs: N=35, n=5568, SMD=-0.51, CI -0.58 to -0.43, p<0.0001).” |
| SZ | Leucht (2009c) | Yes | Overall efficacy | “Four drugs were better than FGAs for overall efficacy, with small to medium ESs (amisulpride −0.31, 95% CI −0.44 to −0.19, p<0.0001], clozapine −0.52, 95%CI −0.75 to −0.29, p<0.0001, olanzapine −0.28, 95%CI −0.38 to −0.18, p<0.0001, and risperidone −0.13 95%CI −0.22 to −0.05, p=0.002]).” |
| SZ | Leucht (2013) | Yes | Overall efficacy | “All drugs were significantly more effective than placebo. The SMDs with 95% CIs for clozapine were: 0.88, CI 0.73 to 1.03; ORs compared with placebo for ACD ranged from 0.43 for the best drug (amisulpride) to 0.80 for the worst drug (haloperidol); for extrapyramidal side-effects 0.30 (clozapine) to 4.76 (haloperidol); and for sedation 1.42 (amisulpride) to 8.82 (clozapine).” |
| SZ | Okhuijsen-Pfeifer (2020) | No | Response predictors | “The results of our meta-analyses suggest that three baseline demographic and clinical features are associated with better clozapine response, i.e. relatively young age, few negative symptoms and paranoid schizophrenia subtype.” |
| SZ | Samara (2014) | Yes | Response to treatment | “Chlorpromazine less efficacious than other four antipsychotics (clomacran, clozapine, olanzapine and zotepine) in the primary outcome response to treatment.” |
| SZ | Sherwood (2012) | No | Response profile | “Compared with other antipsychotic arms, for clozapine the treatment response was greater (d=-0.578, p=0.021).” |
| SZ | Subramanian (2012) | Yes | PANSS-EC | “When zotepine was compared with clozapine, it was clozapine that was found to be more effective in terms of global state (n=59, 1 RCT, RR - No clinically significant response 8.23, CI 1.14 to 59.17). Mental state scores also favored clozapine (n=59, 1 RCT, MD average score (BPRS total, high = poor) 6.00 CI 2.17 to 9.83) and there was less use of antiparkinson medication in the clozapine group (n=116, 2 RCTs, RR=20.96, CI 2.89 to 151.90)” |
| SZ | Szegedi (2012) | No | PANSS | “Relative efficacy was more favorable for asenapine compared with clozapine.” |
| SZ | Tuunainen (2002) | Yes | Overall outcome | “Newer atypical drugs were broadly similar to clozapine when improvement was measured using a psychosis symptom rating scale or a global index. There was a trend for clozapine to be more effective than the comparators for positive symptoms, and less effective for the negative symptoms.” |
| SZ | Tuunainen (2000) | Yes | Overall outcome | “Newer atypical drugs seemed to be broadly similar to clozapine using a clinical global index or trialists' definitions of improvement, but this result was obtained from a relatively small number of studies. Due to the small number of studies and patients, wide confidence intervals were seen when their effectiveness as measured by symptom rating scales was compared. Social functioning was better in patients on newer atypical medication (risperidone) than in those on clozapine, but this finding is based on a single underpowered trial and has to be interpreted with caution.” |
| SZ | Wahlbeck (1999)** | Yes | Overall outcome | “Clozapine-treated patients showed more clinical improvement and experienced significantly fewer relapses during treatment, although the risk of blood dyscrasias in long-term treatment may be as high as 7%. Scores on symptom rating scales showed greater improvement among clozapine-treated patients, who were also more satisfied with their treatment. However, there was no evidence that the superior clinical effect of clozapine is reflected in levels of functioning; on the other hand, global functional and pragmatic outcomes were frequently not reported. Clinical improvement was most pronounced in patients with treatment-resistant illness.” |
| Elderly people with SZ | Krause (2018) | Yes | PANSS | “There were no significant differences between chlorpromazine and clozapine (N=1, SMD=0.15, CI -0.58 to -0.88).” |
| Reduction in TD | Bergman (2018) | Yes | Reduction in TD | “In the long term, there was no difference between clozapine and haloperidol (1 RCT, 39 people, RR=3.36, 95%CI 0.45 to 25.16) on the number leaving the study early.” |
| TD risk | Carbon (2018) | No | TD risk | “Mean raw TD incidence (N=6, n=348, 8.2%, 95%CI 3.9 to 16.6), mean annualized TD incidence 4.2%, 95%CI 1.7 to 6.7).” |
| EPS | Leucht (2003) | Yes | Number of patients with at least one EPS | “Of the new generation drugs, only clozapine was associated with significantly fewer EPS (RD=–0·15, 95% CI –0·26 to –0·4, p=0·008) and higher efficacy than low-potency conventional drugs.” |
| TD risk | Mentzel (2018) | No | Change in TD rating scale score | “The overall effect of switching to clozapine was a significant reduction in TD (n=1060, d=-0.40, p<0.01) especially in the 4 studies that investigated the severity of TD as a primary outcome (n=48, d=-2.56, p=0.02).” |
| Use of antiparkinson medication | Rummel-Kluge (2010) | Yes | Use of antiparkinson medication at least once | “Risperidone was associated with more use of antiparkinson medication than clozapine. Zotepine showed more use of than clozapine. There was no significant difference in use of antiparkinson medication between olanzapine and clozapine (N=6, n=561, RR=1.14, CI 0.6 to 2.19, p=0.69).” |
| First-episode SZ | Tek (2015) | No | Change in weight | “Olanzapine and clozapine caused the highest weight gain compared to placebo.” |
| First-episode SZ | Zhang (2013) | Yes | Overall outcome | “Compared to FGAs, EPS-related outcomes were less frequent with olanzapine, risperidone and clozapine, but weight gain was greater with clozapine, olanzapine and risperidone.” |
| Hospitalisation | Land (2017) | Yes | Hospital use for any reason | “Clozapine significantly reduced the proportion of people hospitalised compared to control medicines (RR=0.74; 95%CI 0.69–0.80, p<0.001, 22 studies, n=44718). There were significantly fewer bed days after clozapine treatment compared to before clozapine treatment in both controlled (MD=-34.41 days; 95% CI -68.22 to -0.60 days, p=0.046, n=162) and uncontrolled studies (MD=-52.86 days; 95%CI: -79.86 days to -25.86 days, p<0.001, n=2917). Clozapine and control medicines had a similar time to rehospitalisation (-19.90 days; 95%CI -62.42 to 22.63 days, p=0.36).” |
| Hypersalivation | Chen (2019) | Yes | Change in sialorrhea | “Improvement in clozapine-induced sialorrhea was seen in meta-analyses of propantheline (studies = 6, risk ratio [RR] 2.38, 95%CI 1.52 to 3.73; NNT 3, 95%CI 1.9 to 2.7), diphenhydramine (studies = 5, RR=3.09, 95%CI 2.36 to 4.03; NNT 2, 95%CI 1.5 to 2.0), chlorpheniramine (studies=2, RR=2.37, 95%CI 1.59 to 3.55; NNT 3, 95%CI 1.6 to 3.5), and benzamide derivatives (OR=6.93, 95%CI 3.03 to 15.86).” |
| Hypersalivation | Syed (2012) | Yes | Change in sialorrhea | “For the outcome of ‘no clinically important improvement’ astemizole and diphenhydramine were more effective than placebo (astemizole: n=97, 2 RCTs, RR 0.61 CI 0.47 to 0.81 NNT 3 CI 2 to 5; diphenhydramine: n=131, 2 RCTs, RR 0.43 CI 0.31 to 0.58, NNT 2, CI 1.5 to 2.5), but the doses of astemizole used were those that can cause toxicity. Data involving propantheline were heterogeneous (I^2^= 86.6%), but both studies showed benefit over placebo.” |
| SZ and intellectual disabilities | Ayub (2015) | No | Overall outcome | No meta-analysis conducted. |
| Metabolic complications | Bak (2014) | No | Change in metabolic parameters | “Unfavorable outcomes for clozapine for the outcome weight gain in a meta-regression.” |
| Metabolic complications | Bartoli (2015a) | No | Adiponectin levels | “There were no differences on adiponectin levels between people taking clozapine and those taking olanzapine (p=0.86), but high heterogeneity was detected (I^2^=82%). Both individuals taking clozapine (p=0.001; I^2^=0%) and those taking olanzapine (p=0.02; I^2^=9%), but not subjects treated with quetiapine (p=0.47; I^2^=0%), had adiponectin levels significantly lower than people taking risperidone.” |
| Metabolic complications | Bartoli (2015b) | No | Adiponectin levels | “Clozapine (p<0.001) and olanzapine (p=0.04) were associated with adiponectin levels lower than controls.” |
| Metabolic complications | Buhagiar (2019) | No | Lipid metabolism | “Clozapine, olanzapine and risperidone showed mildly elevated associations with dyslipidaemia “caseness” (clozapine, OR=1.26, 95%CI 1.16 to 1.38; olanzapine, OR=1.29, 95%CI 0.89 to 1.87; risperidone, OR=1.05, 95%CI 0.80 to 1.37) compared with first-generation antipsychotics, but heterogeneity was high (all I^2^>50%, p<0.05). Clozapine was also associated with increased triglycerides (SMD=0.51, 95%CI 0.21 to 0.81, I^2^ = 5.74%), but not with cholesterol.” |
| Metabolic complications | Correll (2016) | Yes | Overall outcome | “Clozapine-topiramate cotreatment moderated greater efficacy, but less weight loss, compared to topiramate-nonclozapine antipsychotic combinations.” |
| Metabolic complications | Mitchell (2011) | No | MetS rates in Sz | “Regarding prescribed antipsychotic medication, highest rates for MetS were seen in those prescribed clozapine (51.9%) and lowest rates of MetS in those who were unmedicated (20.2%).” |
| Metabolic complications | Pillinger (2019) | Yes | Change in metabolic outcomes | “MDs for weight gain compared with placebo ranged from −0.23 kg (95% CI −0.83 to 0.36) for haloperidol to 3.01 kg (1.78 to 4.24) for clozapine; for triglycerides from −0.01 mmol/L (−0.10 to 0.08) for brexpiprazole to 0.98 mmol/L (0.48 to 1.49) for clozapine; for glucose from −0.29 mmol/L (−0.55 to −0.03) for lurasidone to 1.05 mmol/L (0.41 to 1.70) for clozapine and for total-cholesterol from −0.09 mmol/L (–0.24 to 0.07) for cariprazine to 0.56 mmol/L (0.26 to 0.86) for clozapine.” |
| Metabolic complications | Potvin (2015) | No | Leptin blood level change | “Olanzapine, clozapine, and quetiapine produced moderate leptin elevations, whereas haloperidol and risperidone were associated with small (nonsignificant) leptin changes. |
| Metabolic complications | Rummel-Kluge (2010) | Yes | Weight change | “Olanzapine produced more weight gain than all other second-generation antipsychotics except for clozapine where no difference was found. Clozapine produced more weight gain than risperidone. No significant difference in cholesterol increase between olanzapine and clozapine. No difference in glucose increase between olanzapine and clozapine.” |
| Metabolic complications | Siskind (2016) | Yes | Weight loss, BMI | “Metformin was superior to placebo in terms of weight loss (-3.12kg, 95%CI -4.88kg to -1.37kg) and BMI (-1.18kg/m^2^, 95%CI -1.76kg/m^2^ to -0.61kg/m^2^). Metformin significantly improved three of the five components of metabolic syndrome; waist circumference, fasting glucose and triglycerides. Sensitivity analysis on study quality and duration did not greatly impact results.” |
| Metabolic complications | Siskind (2018) | Yes | Change in body weight | “Weight loss with GLP-1RAs was greater for clozapine-/olanzapine-treated patients (n=141) than other antipsychotics (n=27) (4.70kg, 95%CI 3.13 to 6.27 vs 1.5kg 95%CI -1.47 to 4.47) (p<0.001). |
| Metabolic complications | Smith (2008) | No | Risk for diabetes with FGA vs. SGA | “Relative risks for separate second-generation drugs (vs. FGA) were as follows: risperidone 1.16 (95%CI 0.99 to 1.35; n=6 studies), quetiapine 1.28 (95%CI 1.14 to 1.45; n=3 studies), olanzapine 1.28 (95%CI 1.12 to 1.45; n=8 studies) and clozapine 1.39 (95%CI 1.24–1.55; n=7 studies).” |
| Metabolic complications | Srisurapanont (2015) | Yes | Overall outcomes | “The pooled SMDs suggested trends of aripiprazole augmentation benefits on overall psychotic [-0.40, CI -0.87 to 0.07) (n=3; Z=1.68, p=0.09; I^2^=68%)], positive [-1.05 (CI -2.39 to 0.29) (n=3; Z=1.54, p=0.12; I^2^=94%)], and negative [-0.36 (CI -0.77 to 0.05) (n=3; Z=1.74, p=0.08; I^2^=54%)] symptoms. Despite of no benefit on three cardiometabolic indices (i.e., fasting plasma glucose, triglyceride, and high-density lipoprotein), aripiprazole augmentation was superior for weight change with a MD (95% CI) of -1.36 kg (-2.35 to -0.36) (n=3; Z=2.67, p=0.008; I^2^=39%) and LDL-cholesterol with a MD of -11.06 mg/dL (-18.25 to -3.87) (n=3; Z=3.02, p=0.003; I^2^=31%). Aripiprazole augmentation was not correlated with headache and insomnia but significantly associated with agitation/akathisia (RR=7.59, 95%CI 1.43 to 40.18) (n=3; Z= 2.38, p=0.02; I^2^=0%) and anxiety (RR=2.70, 95%CI 1.02 to 7.15) (n=1; Z=2.00, p=0.05).” |
| Metabolic complications | Vancampfort (2015) | Yes | Prevalence of MetS | “MetS risk was significantly higher with clozapine and olanzapine (except vs. clozapine) than other antipsychotics, and significantly lower with aripiprazole than other antipsychotics (except vs. amisulpride).” |
| Metabolic complications | Zhang (2017) | No | Changes in blood glucose levels | “SUCRA values were used to determine the hierarchy of the antipsychotic treatments. A larger SUCRA value indicates a higher ranking for the drug. In our study, the SUCRA values indicated the following hierarchy among the 13 treatments: 69.4, 52.9, 64.4, 39.7, 28.9, 23.8, 32.0, 84.1, 82.8, 19.2, 43.2, 49.8 and 59.9% for placebo, amisulpride, aripiprazole, asenapine, sertindole, clozapine, haloperidol, ziprasidone, lurasidone,olanzapine, paliperidone, quetiapine and risperidone, respectively. These probabilities may inform the ranking of these treatments in terms of their glucose metabolism-related adverse effects.” |
| Metabolic complications | Zheng (2016) | Yes | Overall outcome | “Compared to placebo/antipsychotic monotherapy, topiramate led to significantly reduced/lower endpoint body weight (trials=15, n=713, WMD=-2.75 kg, 95%CI -4.03 to -1.47, p<0.0001; Significance was lost for studies with cotreatment of clozapine (studies=4, n=159, WMD=-1.58, 95%CI -3.93 to 0.67, p=0.19).” |
| Metabolic complications | Zimbron (2016) | No | Change in metabolic outcomes | “Effective pharmacological treatments for clozapine-induced obesity and metabolic syndrome include metformin, aripiprazole, and Orlistat (in men only). Meta-analysis of three studies showed a robust effect of metformin in reducing body mass index and waist circumference but no effects on blood glucose, triglyceride levels, or HDL levels. In addition, there is limited evidence for combined calorie restriction and exercise as a non- pharmacological alternative for the treatment of clozapine-induced obesity, but only in an inpatient setting.” |
| Mortality | Vermeulen (2019) | No | Mortality rate | “Altogether, 24 studies reported on 1327 deaths from any causes during 217 691 patient years in patients treated with clozapine. The unadjusted mortality rate in 22 unique samples during a follow-up of 1.1–12.5 (median=5.4) years was 6.7 (95%CI 5.4 to 7.9) per 1000 patient years. Long-term, crude mortality rate ratios were not significantly lower in patients ever treated with clozapine during follow-up, but significantly lower in patients continuously treated with clozapine compared to patients with other antipsychotics (mortality rate ratio=0.56, 95%CI 0.36 to 0.85, p-value=0.007).” |
| Multi-episode SZ | Huhn (2019) | Yes | PANSS, BRPS | “Effect size estimates suggested all antipsychotics reduced overall symptoms more than placebo (although not statistically significant for six drugs), with SMDs ranging from –0·89 (95%CI –1·08 to –0·71) for clozapine to –0·03 (–0·59 to 0·52) for levomepromazine (40 815 participants).” |
| Negative symptoms in SZ | Krause (2018) | Yes | Negative symptoms | “One study comparing clozapine with haloperidol did not show a difference in terms of negative and positive symptoms.” |
| Neutropenia | Myles (2018) | Yes | Rates of neutropenia | “The incidence of clozapine-associated neutropenia was 3.8% (95%CI 2.7 to 5.2%) and severe neutropenia 0.9% (95% CI: 0.7–1.1%). The incidence of death related to neutropenia following prescription of clozapine was 0.013% (95%C: 0.01 to 0.017%). The case fatality rate of severe neutropenia was 2.1% (95%CI 1.6 to 2.8%). The peak incidence of severe neutropenia occurred at one month of exposure and declined to negligible levels after one year of treatment.” |
| Neutropenia | Myles (2019) | Yes | Rates of neutropenia | “The risk ratio was not significantly increased in clozapine-exposed groups compared to exposure to other antipsychotic medications (Mantel–Haenszel risk ratio = 1.45, 95%CI 0.87 to 2.42). This also applied to severe neutropenia (absolute neutrophil count <500 per μL) when compared to other antipsychotics (Mantel–Haenszel risk ratio=1.65, 95%CI 0.58 to 4.71). The relative risk of neutropenia associated with clozapine exposure was not significantly associated with any individual antipsychotic medication.” |
| Parkinson’s disease psychosis (PDP) and drug-induced psychosis in PD | Frieling (2007) | No | Change in psychotic symptoms | “Two trials compared low-dose clozapine versus placebo with a significantly better outcome for clozapine regarding efficacy and motor functioning. In one trial clozapine was compared against quetiapine showing equivalent efficacy and tolerability. However, in two placebo-controlled trials quetiapine failed to show efficacy. In two further placebo-controlled trials olanzapine did not improve psychotic symptoms and significantly caused more extrapyramidal side effects. Based on randomized trial-derived evidence which is currently available, only clozapine can be fully recommended for the treatment of DIP in PD.” |
| Parkinson’s disease psychosis (PDP) and drug-induced psychosis in PD | Iketani (2017) | No | BRPS, UPDRSM | “Pooled estimates of each posterior distribution based on the BPRS were as follows: clozapine, -2.0 (CI -6.7 to 2.7); olanzapine, 0.5 (CI -2.3 to 3.4); quetiapine, 0.3 (CI -3.9 to 4.5); and risperidone, -4.7 (CI -57.4 to 53.3). Based on the UPDRS-III, the pooled estimates were clozapine, 0.7 (CI -3.8 to 4.3); olanzapine, 2.8 (CI 0.8 to 5.1); quetiapine, 3.3 (-0.7 to 5.8); and risperidone, 4.5 (CI -57.7 to 63.4). Although clozapine had an effective and relatively safe profile, all atypical antipsychotics included in the present study may be unsafe, as they may worsen motor function when compared to placebo.” |
| Parkinson’s disease psychosis (PDP) and drug-induced psychosis in PD | Jethwa (2015) | No | BPRS, CGI, UPDRSM | “Data from the two clozapine trials can be combined in a meta-analysis by the CGI and UPDRSM as measures of efficacy and safety, respectively. Clozapine appears to result in an improvement in CGI scores (95% CI −1.23 to −0.96). Though clozapine demonstrated some efficacy over placebo in alleviating psychotic symptoms, a direct comparison cannot be made with other studies employing the BPRS.” |
| Parkinson’s disease psychosis (PDP) and drug-induced psychosis in PD | Zhang (2019) | No | Various psychosis outcome scales | “Clozapine was associated with significant differences in the rates of CGI (WMD =−1.10, 95%CI −1.23 to −0.98, p<0.001, I^2^=0%, N=2) and UPDRS Motor (WMD=−1.75, 95% CI: −2.54 to −0.96, p<0.001, I^2^=0%, N=2).On the other hand, the results of the UPDRS (WMD=−1.79, 95%CI −4.57 to 1.00, p=0.21, I^2^=43%, N=2) and MMSE (WMD =0.11, 95%CI −0.12 to 0.33, p=0.36, I^2^=0%, N=2) showed no significant differences between the clozapine group and placebo. Based on the three trials included in this study, the adverse effects of clozapine include sedation, hypotension, agranulocytosis and metabolic syndrome.” |
| Pneumonia | Dzahini (2018) | No | Risk for pneumonia | “All antipsychotics with data from ⩾2 studies allowing meta-analysis, were associated with a significantly increased pneumonia risk (i.e. haloperidol, olanzapine, clozapine, risperidone, quetiapine, zotepine). Patients re-exposed to clozapine had a higher risk for recurrent pneumonia (RR=1.99, 95%CI 1.10 to 3.59) than those who were not re-exposed.” |
| Psychosocial function in SZ | Olagunju (2018) | Yes | Change in psychosocial function | “Clozapine showed beneficial effects on psychosocial function, but both short-term trials [n=3; comparing 99 people taking clozapine with 97 controls, SMD=0.04; 95%CI −0.24 to 0.32; p=0.77; I^2^=0%)] and long-term trials [n=5; comparing 415 people taking clozapine with 427 controls (SMD=0.05; 95%CI −0.16 to 0.27; p=0.64; I^2^=50%)] showed no superiority of clozapine to other antipsychotics in this regard.” |
| Relapse in SZ | Kishimoto (2013) | Yes | Study-defined relapse | “When requiring ≥3 trials per individual antipsychotic, neither risperidone (n=1124, RR=0.75, CI 0.56 to 1.00, p=0.05, I^2^=55%), clozapine (n=355, RR=0.72, 95%CI 0.47 to 1.10, p=0.12, I^2^=0%) or olanzapine (n=1140, RR=0.88, CI 0.70 to 1.10, p=0.27, I^2^=22%) were statistically superior to FGAs in preventing relapse.” |
| Relapse in SZ | Leucht (2003) | Yes | Relapse rate | “No statistically significant differences were found for amisulpride and clozapine, although the findings were in favor of these new drugs vs. FGA.” |
| Second-line treatment in SZ | Cheine (1998) | No | Psychotic symptom outcome | “In the short term, the OR for clinical improvement on clozapine treatment when compared to conventional treatment is calculated to be 2.4 (95%CI 1.7 to 3.5) and the NNT is 7 (95%CI 5 to 13).” |
| Second-line treatment in SZ | Okhuijsen-Pfeifer (2018) | Yes | Treatment response | “This meta-analysis comparing clozapine to a miscellaneous group of antipsychotics revealed a significant benefit of clozapine (Hedges’ g=0.220, p=0.026, 95%CI 0.026 to 0.414), with no evidence of heterogeneity. In addition, a sensitivity analysis revealed a significant benefit of clozapine over risperidone (Hedges’ g=0.274, p=0.030, 95%CI 0.027 to 0.521).” |
| Sexual dysfunction (SD) | Serretti (2011) | No | Rate of total sudden death related to AP | “Quetiapine, ziprasidone, perphenazine, and aripiprazole were associated with relatively low sudden death rates (16–27%), whereas olanzapine, risperidone, haloperidol, clozapine, and thioridazine were associated with higher rates (40–60%).” |
| Hostility | Faay (2018) | No | Change in hostility scores | “An analysis comparing clozapine to typical antipsychotics showed a moderate effect size (0.415) for improvement in hostility, with low heterogeneity.” |
| Suicidality | Hennen (2004) | No | Risk of suicidal behavior, suicide attempts | “Substantially lower overall risk of suicidal behaviors with clozapine vs. other treatments (RR=3.3; 95%CI 1.7 to 6.3; p=0.0001). For completed suicides, the RR was 2.9 (95%CI 1.5 to 5.7); p=0.002).” |
| Aggression vs. others | Khushu (2016) | Yes | Change in aggression score | “When haloperidol was compared with olanzapine or clozapine, skewed data (n=83) at high risk of bias suggested some advantage in terms of scale scores of unclear clinical meaning for olanzapine/clozapine for ’total aggression’.” |
| TRS | Chakos (2001) | No | Overall outcome | “There was a main effect of treatment (F=8.51, df=1, 8, p<0.05), with greater reduction in psychopathology in the clozapine-treated groups compared to those treated with a typical antipsychotic.” |
| TRS | Mizuno (2019) | Yes | Total symptoms | “Relative variability in change of total symptoms did not differ significantly between clozapine and other antipsychotics in TRS studies (VR = 1.84; 95%CI 0.85–4.02). These findings were similar with CVR, and for positive and negative symptoms. Clozapine was superior to other antipsychotics in improving total  symptoms in both TRS (g=0.34; 95%CI 0.13–0.56) and non-TRS (g=0.20; 95%CI 0.08–0.32) studies. Furthermore, clozapine was superior in improving positive symptoms in both study groups, but not for negative symptoms.” |
| TRS | Moncrieff (2003) | No | Change in psychotic symptom scores | “Meta-regression showed that shorter study duration, financial support from a drug company and higher baseline symptom score consistently predicted greater advantage of clozapine. |
| TRS | Samara (2016) | Yes | Overall change in symptoms | “Clozapine was more effective than haloperidol (−0.22; CI −0.38 to −0.07) and sertindole (−0.40; CI −0.74 to −0.04); and risperidone was more effective than sertindole (−0.32; CI −0.63 to −0.01). A pattern of superiority for olanzapine, clozapine, and risperidone was seen in other efficacy outcomes, but results were not consistent and effect sizes were usually small.” |
| TRS | Siskind (2017)*** | Yes | Response rates | “Overall, 40.1% (95%CI 36.8% to 43.4%) responded, with a mean reduction in PANSS of 22.0 points (95%CI 20.9 to 23.1), a reduction of 25.8% (95%CI 24.7% to 26.9%) from baseline. These reductions are clinically meaningful. A 40% response rate to clozapine suggests that 12% to 20% of people with schizophrenia will be ultra-resistant.” |
| TRS | Siskind (2016) | Yes | Change in symptoms, response rates | “Clozapine was associated with a greater improvement in score when all time frames were combined, using the last reported time point in each study (SMD=70.29, 95%CI 70.49 to 70.09, p=0.005; 24 studies, n=1858). Clozapine had a greater effect on positive symptoms with statistically superior outcomes at all time points compared with negative symptoms where benefits were only seen in the short term. People taking clozapine were significantly more likely to respond in the short term (RR=1.17, 95%CI 1.07 to 2.73, p=0.03; 8 studies, n=1218). The absolute risk reduction was 12.48% (95%CI 7.52 to 17.43).” |
| TRS | Souza (2013) | No | Response rate | “Olanzapine and clozapine had similar effects on dropout rates (RR=0.93, 95%CI 0.77 to 1.12), PANSS total endpoints (SMD=0.21, 95%CI –0.04 to 0.46), and PANSS total mean changes (SMD=0.08, CI95% –0.01 to 0.027). Clozapine was superior to olanzapine for PANSS positive (SMD=0.51, 95%CI 0.17 to 0.86) and negative (SMD=0.50, 95%CI 0.16 to 0.85) subscales. There was a trend toward high doses of olanzapine producing higher effect sizes for this drug.” |

***Supplementary Table 2: Summary of outcomes of included meta-analyses***

*Legend:*

*meta-analysis Duggan et al., 2005 was published as third update (after 2000 and 2003) in *Cochrane Database of systematic reviews* and thus only this version (2005) was reviewed.

**meta-analysis *“Evidence of Clozapine’s effectiveness in Schizophrenia: A Systematic Review and Meta-Analysis of Randomized Trials” Am J Psychiatry, 1999; 156:990-999* was published as Cochrane review in *The Cochrane Library 1999, Issue 4*. Only the publication in Am J Psychiatry was reviewed since clozapine-related findings were identical.

***used data from Siskind et al (2016).

*Abbreviations:*

ACD=all-cause discontinuation, AP=antipsychotic, BRMS= Bech-Rafaelsen Mania Scale, BPRS=Brief Psychiatric Rating Scale, CGI=Clinical Global Impression Severity Scale, CI=confidence interval, CLZ=clozapine, C/D ratio=concentration to dose ratio, C-O-H=Cochrane-Mantel-Haenszel, CPZ=chlorpromazine, DIP=drug-induced psychosis, FGA=first-generation antipsychotic, HR=Hazard ratio, MD=mean difference, MES= multi-episode schizophrenia, MetS, NA=not applicable, NNT=number needed to treat, PANSS=Positive and Negative Syndrome Scale, PD= Parkinson disease, PDP=Parkinson disease psychosis, PLC=placebo, RCT=randomized controlled trial, RD=risk difference, RR=relative risk, SCD=sudden cardiac death, SD=standard deviation, SGA=second-generation antipsychotic, SMD=standardized mean difference, SUD=sudden unexpected death, SZ=schizophrenia, TD=tardive dyskinesia, TRS=treatment-resistant schizophrenia, UPDRS-III=Unified Parkinson’s Disease Rating Scale parts III, UPDRSM=Unified Parkinson’s Disease Rating Scale –Motor Subscale, WMD=weighted mean difference

“…”: Cited according to the wording of the original publication as an exact quote.
