# Supplementary material for: Efficacy and safety of clozapine in psychotic disorders—a systematic quantitative meta-review
Source: Transl Psychiatry. 2021 Sep 22;11:487. doi: 10.1038/s41398-021-01613-2 (PMC8458455; doi:10.1038/s41398-021-01613-2)

S 1 Substance abuse as continuous outcome

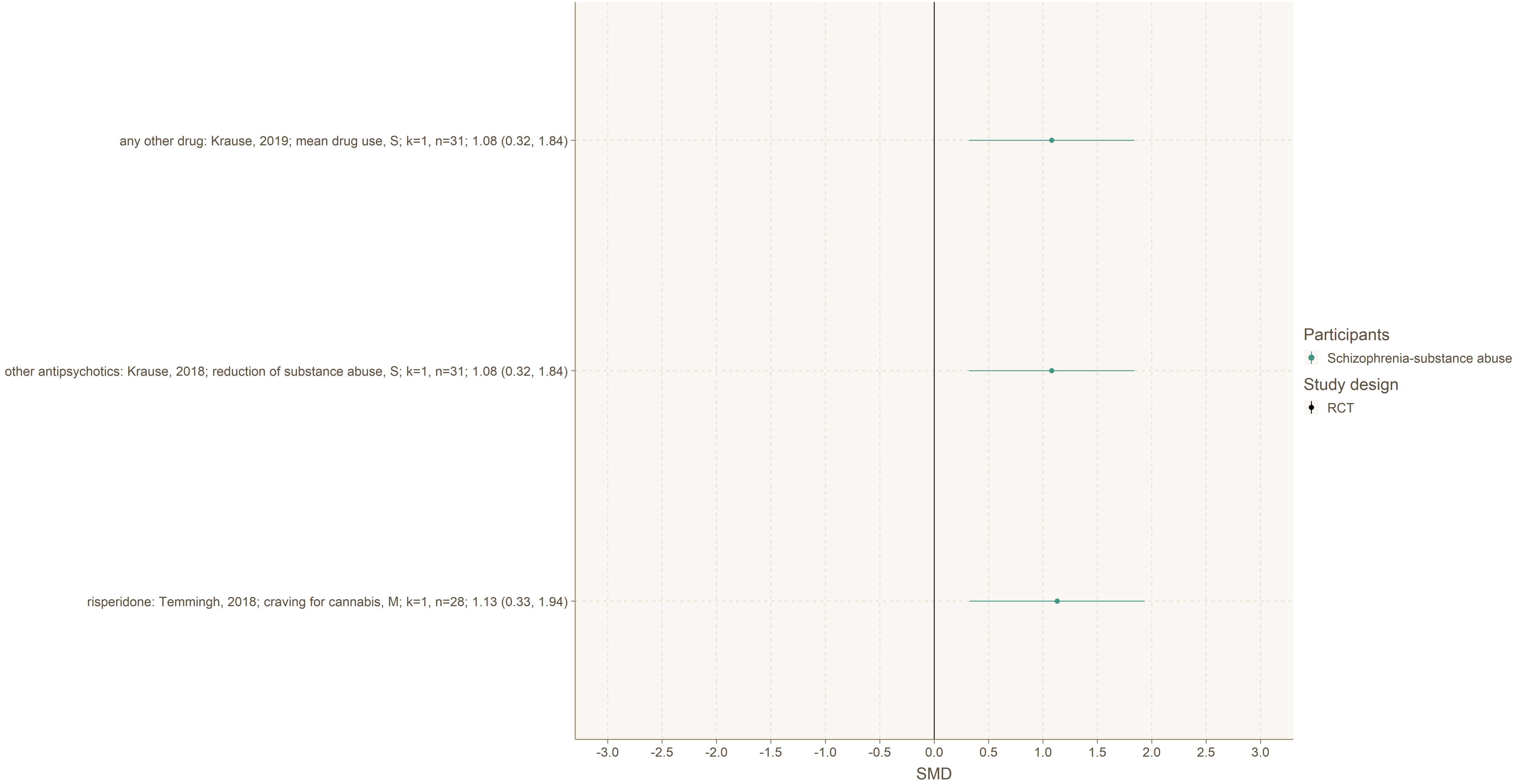

S 2 Substance abuse as dichotomous outcome

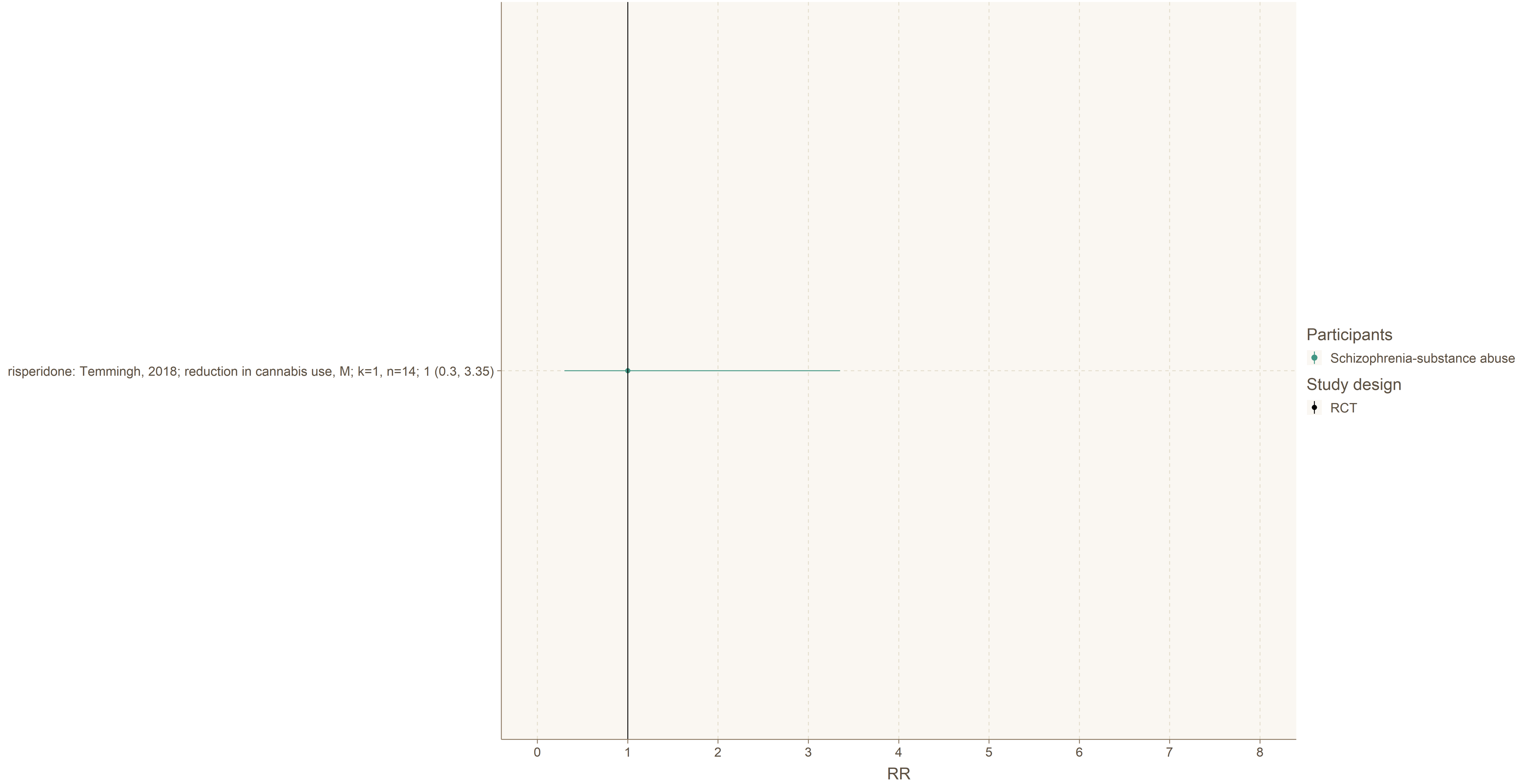

S 3 Other symptoms (cognition, self-injury) as dichotomous outcome

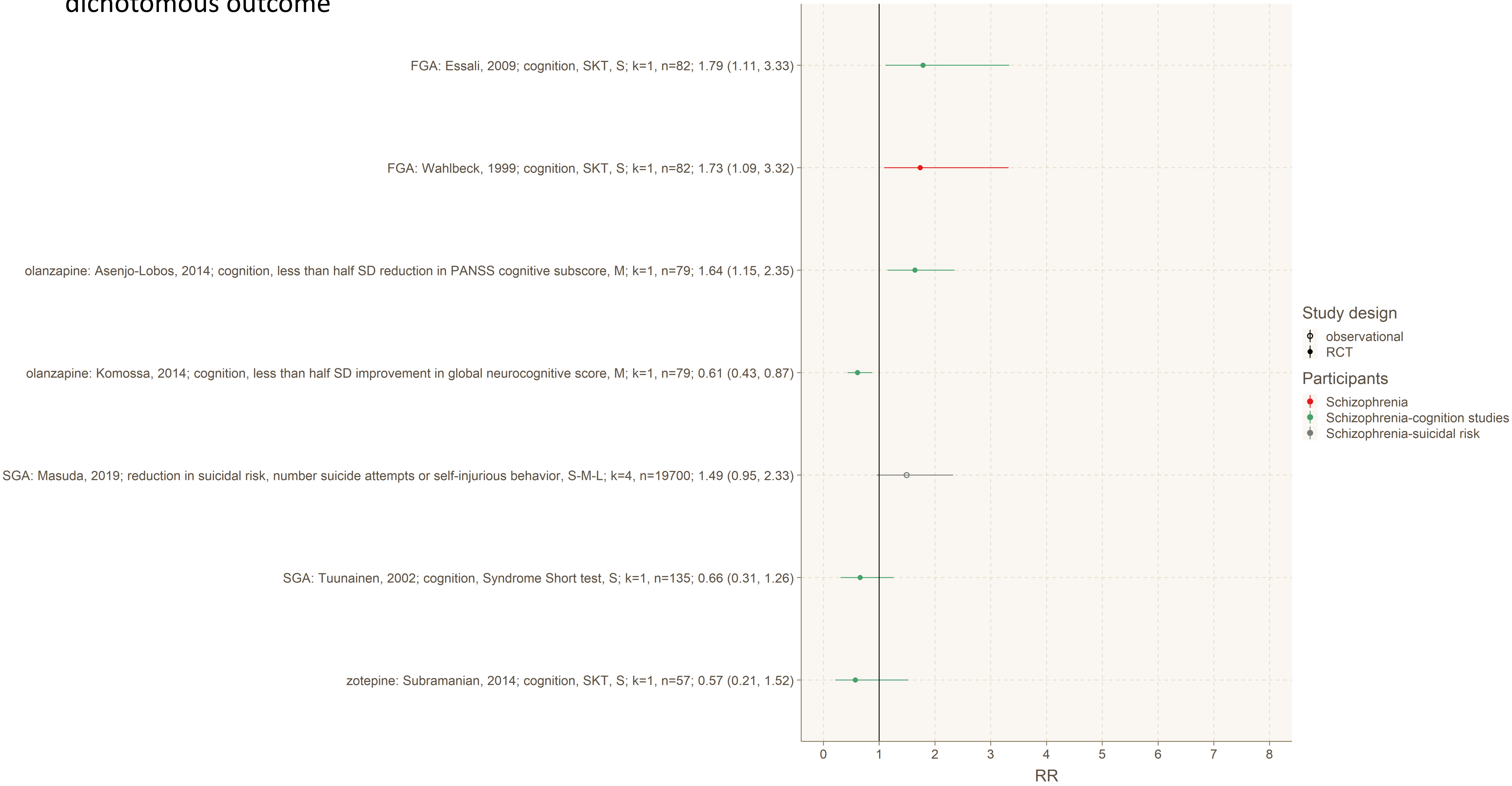

S 4 Needing additional medication as dichotomous outcome

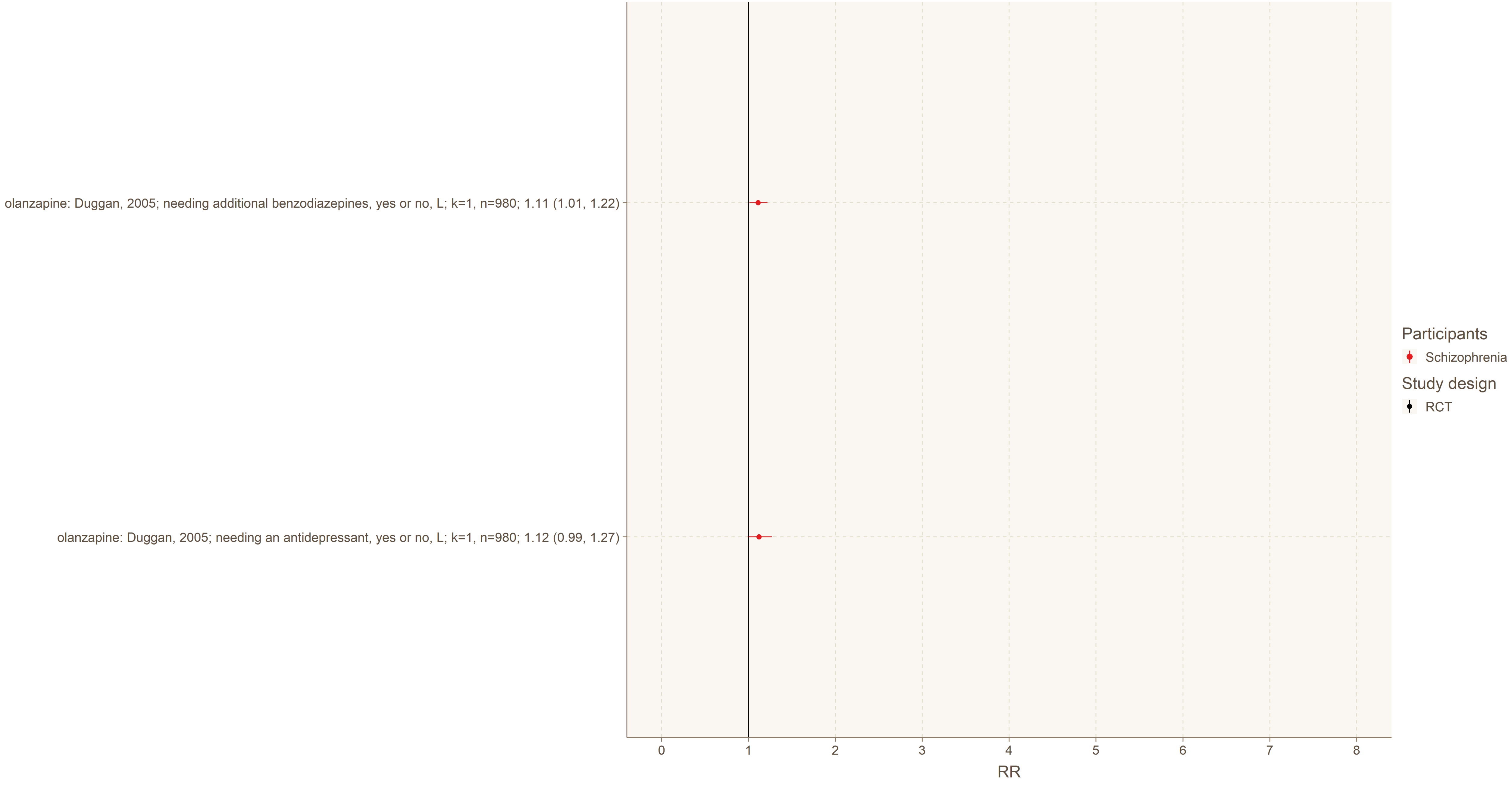

S 5 Blood pressure as continuous outcome

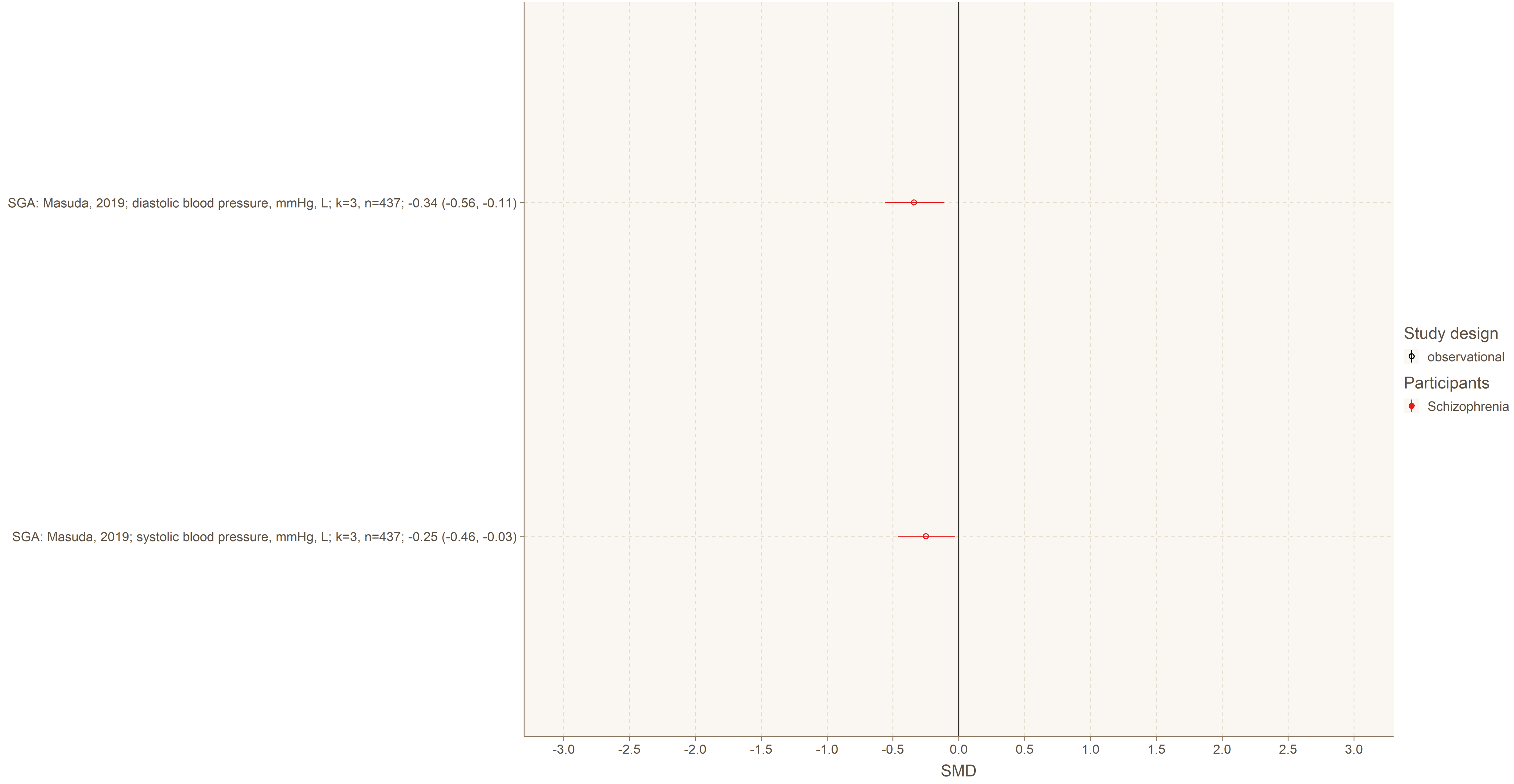

S 6 Cognitive side effects as continuous outcome

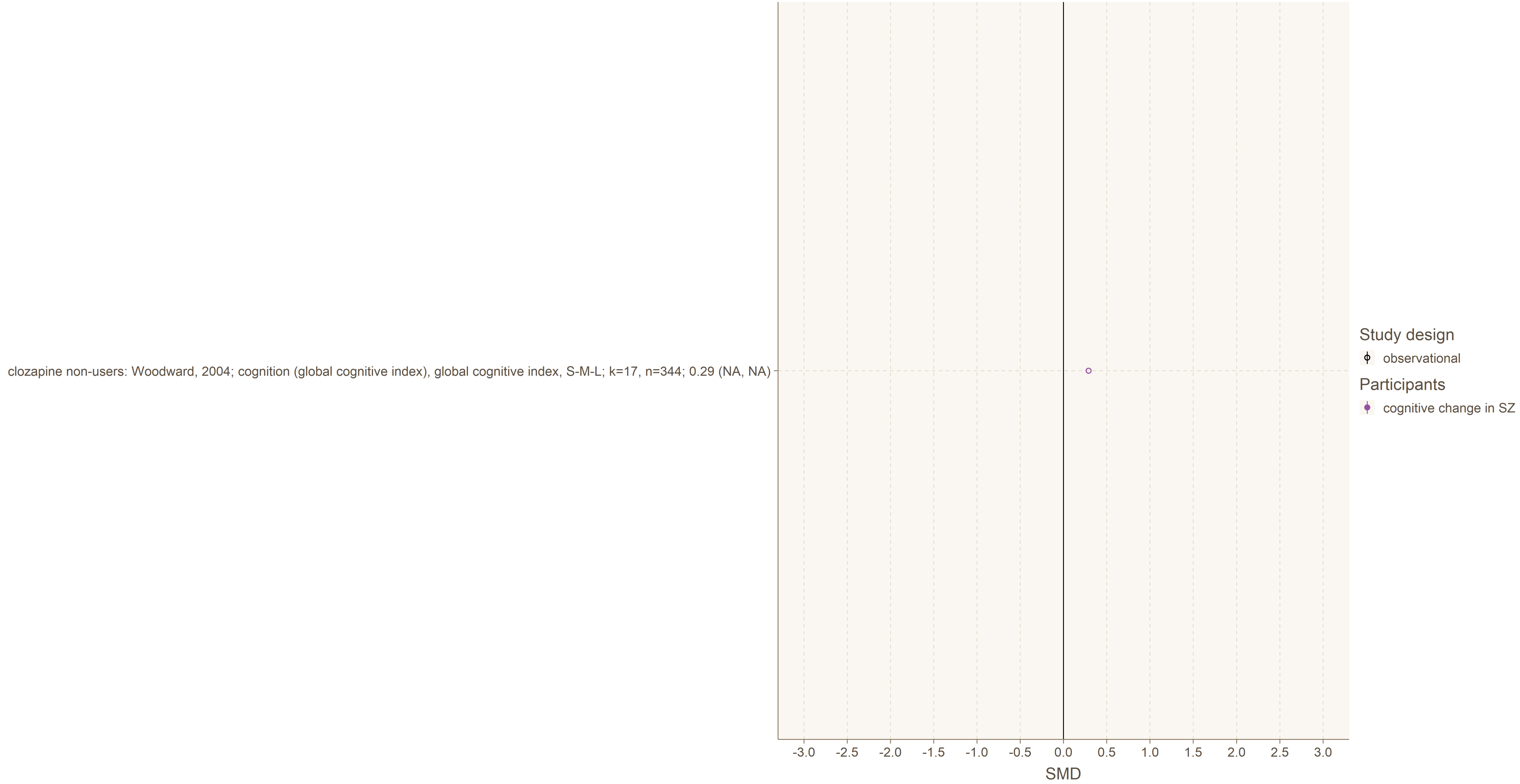

S 7 Prolactin levels as continuous outcome

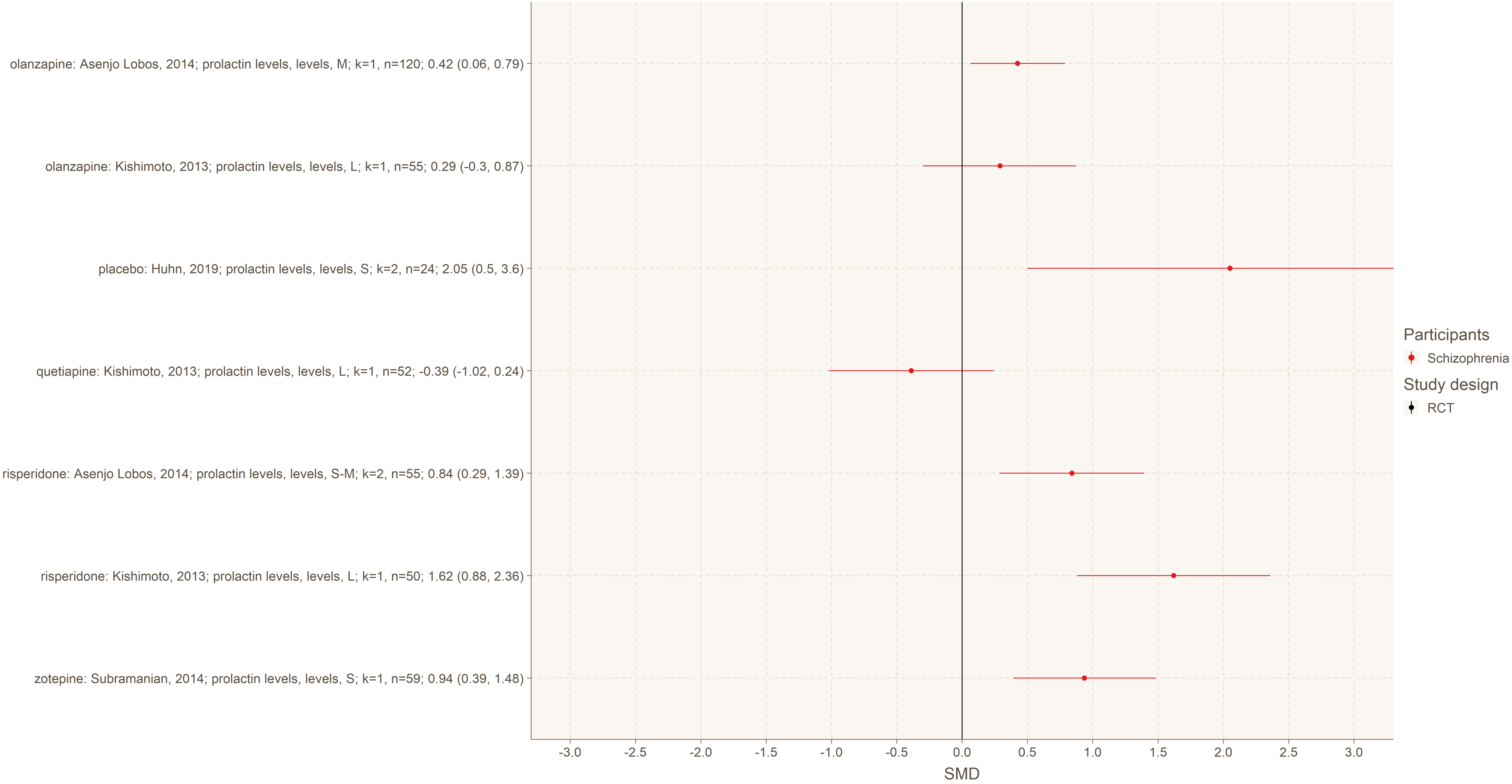

S 8 Motor function as continuous outcome

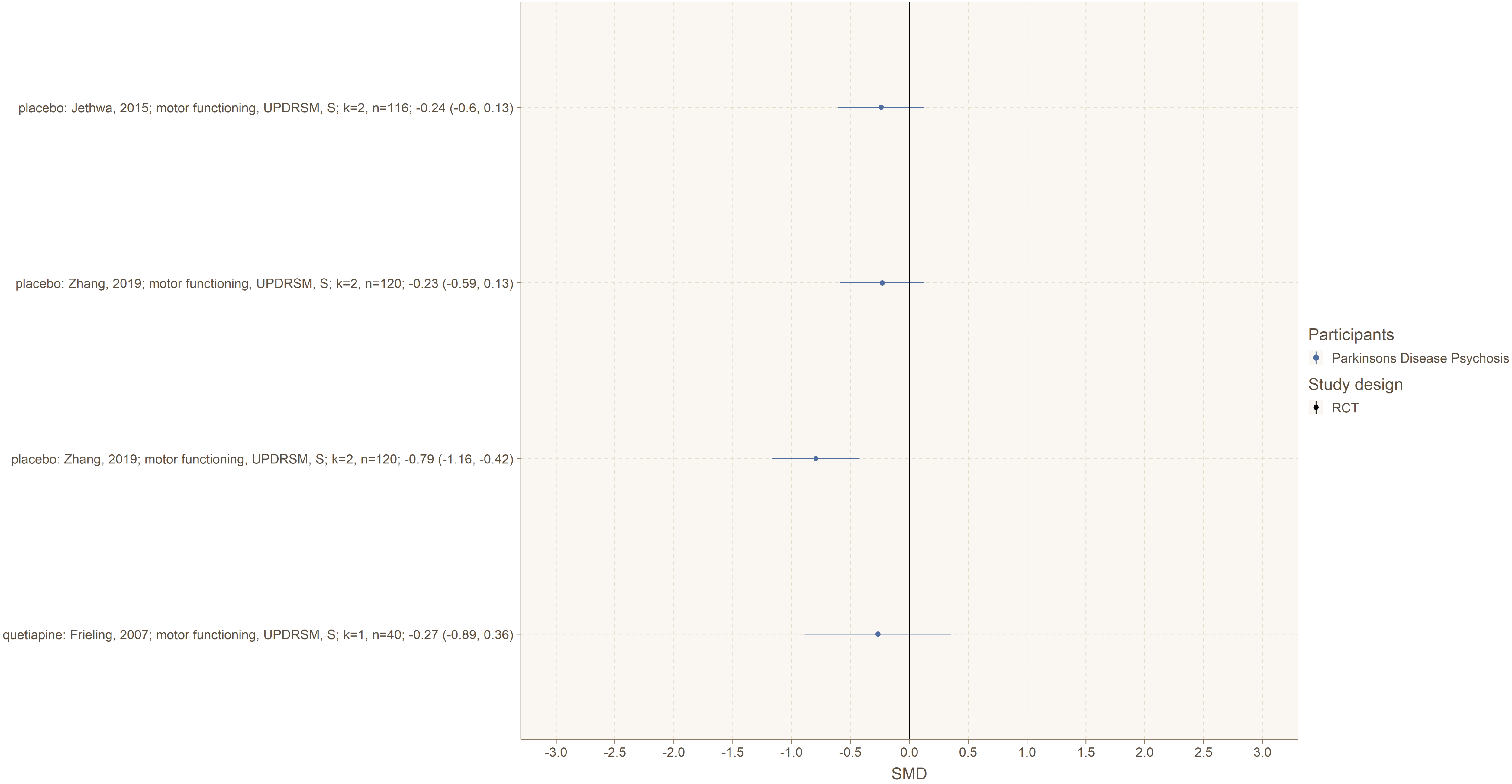

S 9 Diabetes as dichotomous outcome

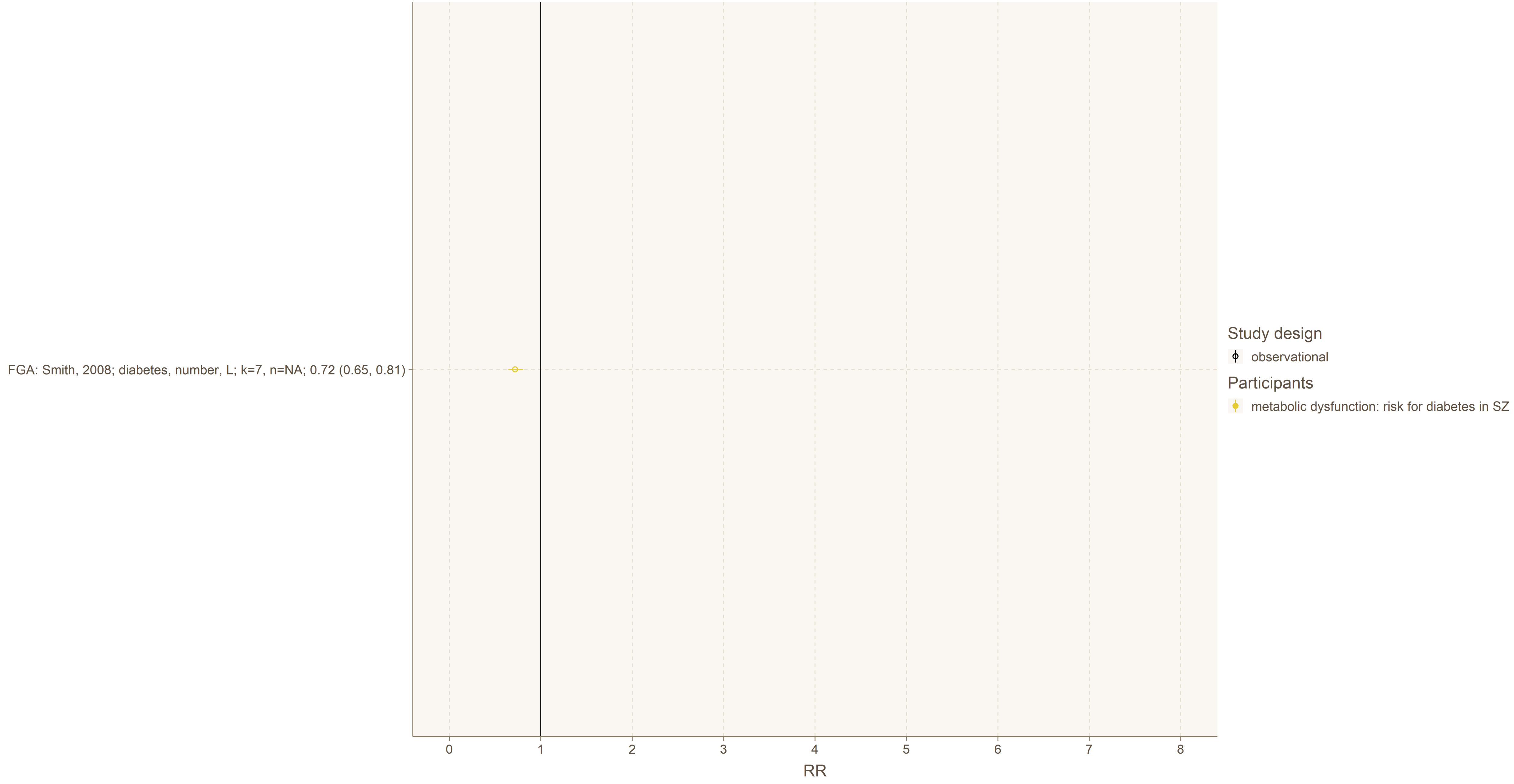

S 10 High temperature as dichotomous outcome

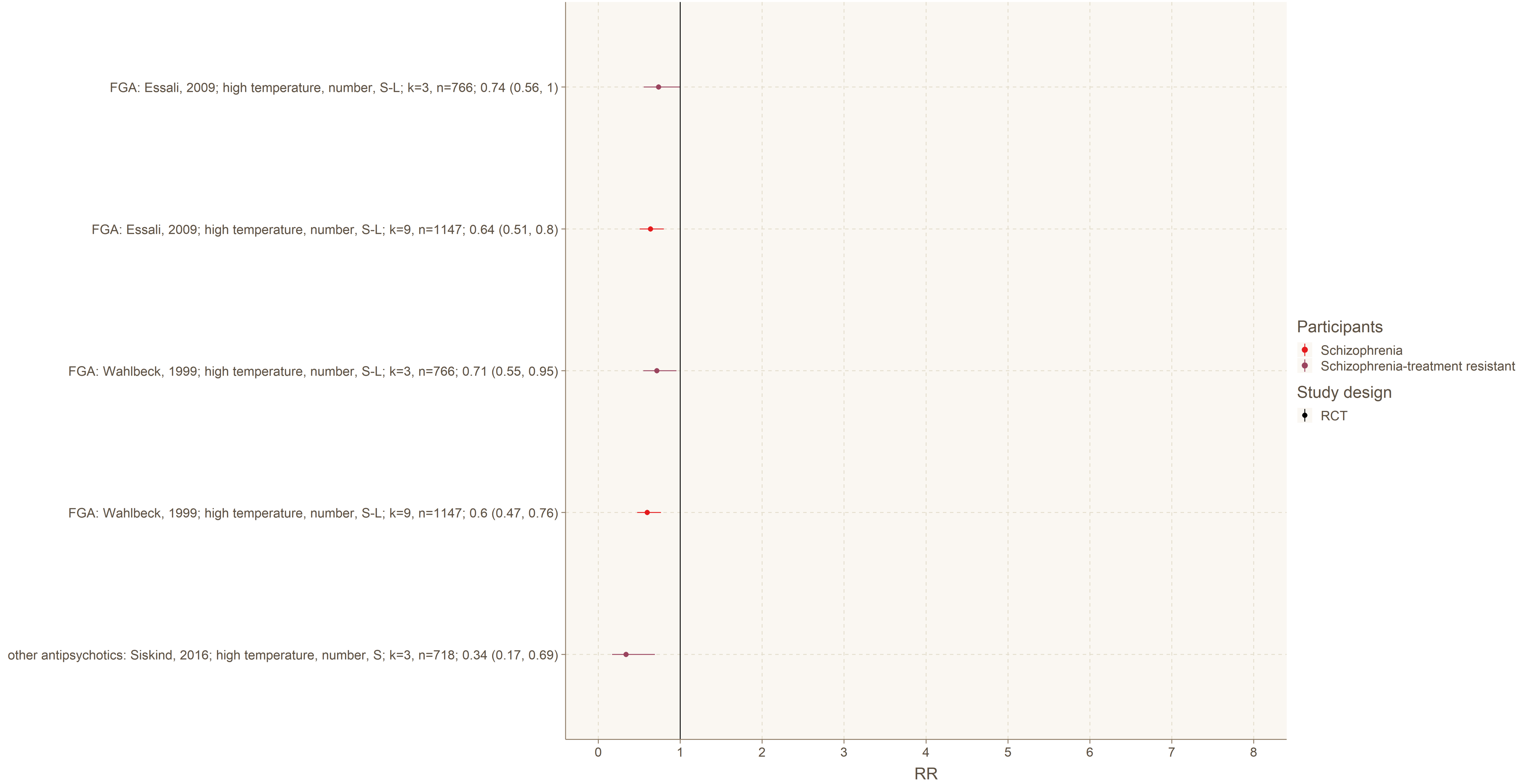

S 11 Occupation as dichotomous outcome

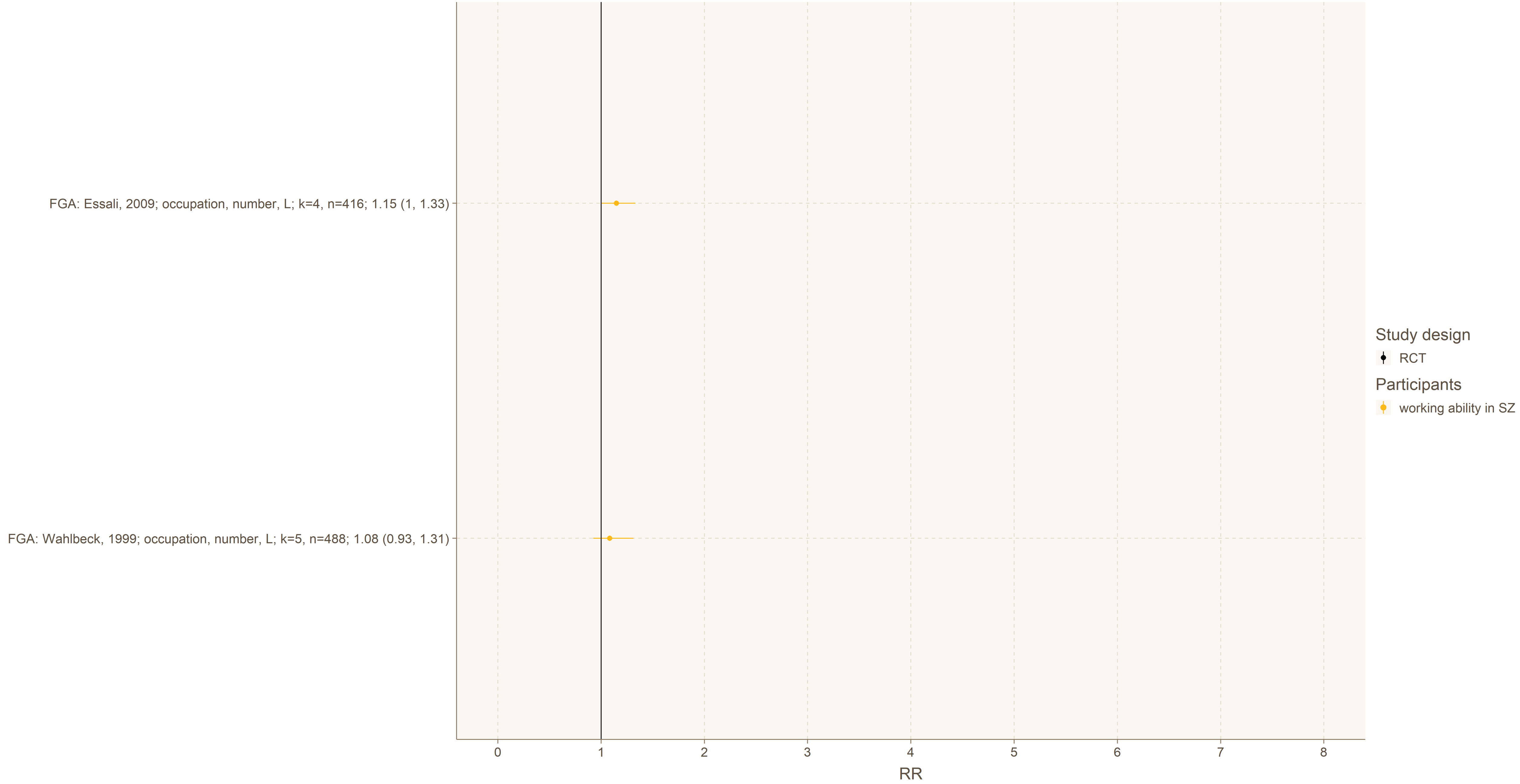

S 12 Gastrointestinal symptoms and sign (liver functions, nausea/vomiting) as dichotomous outcome

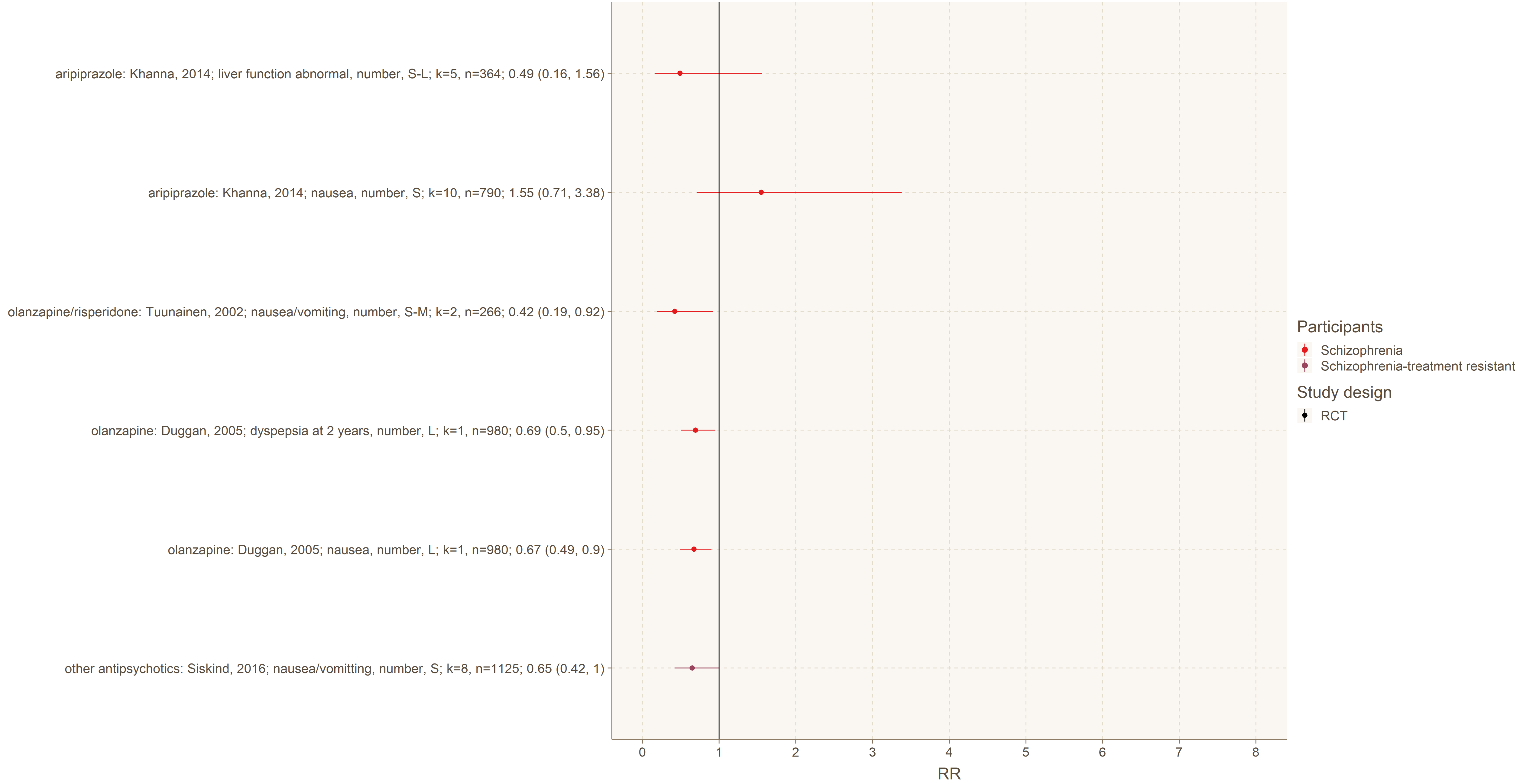

S 13 Mortality as dichotomous outcome

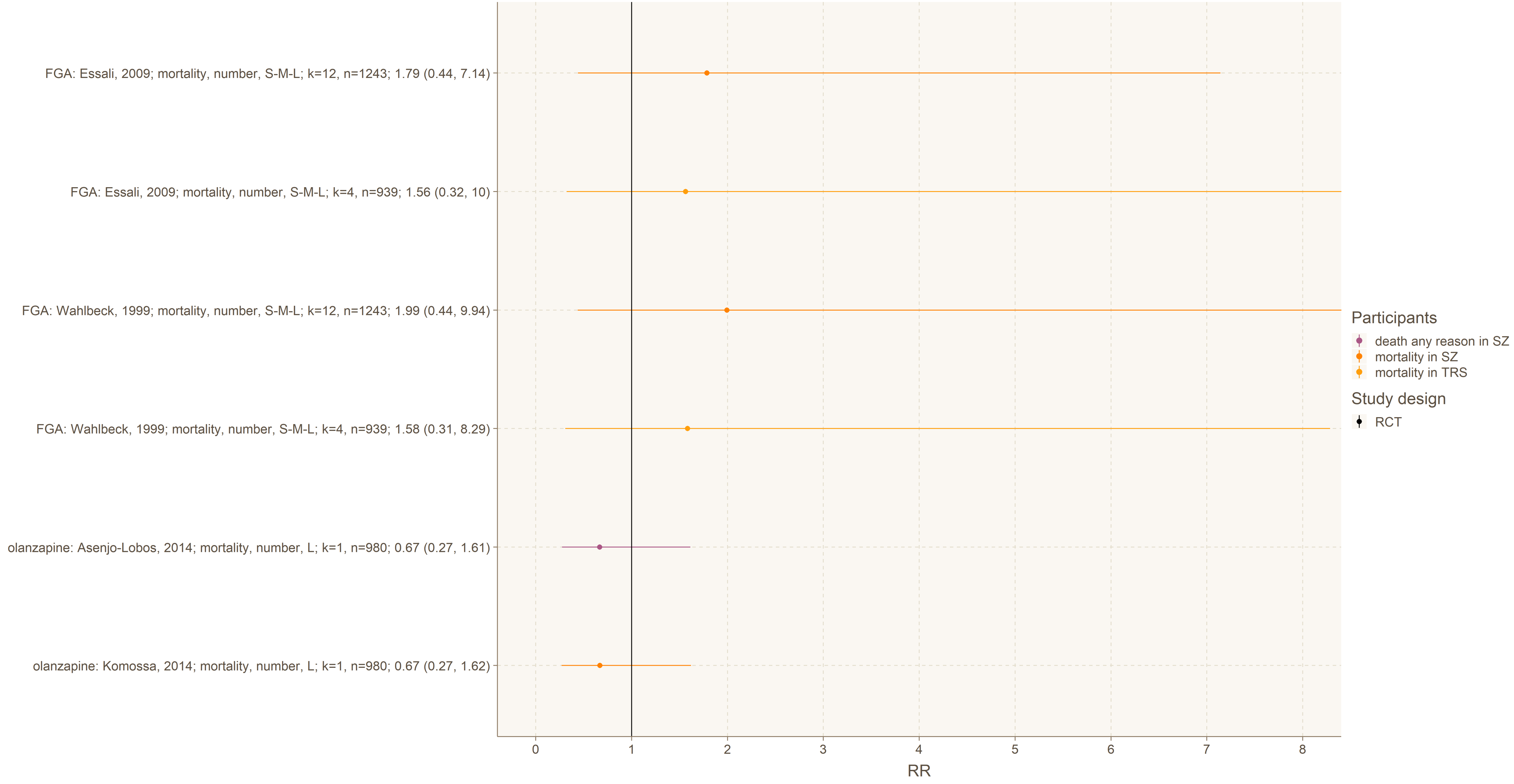

S 14 Sexual and reproductive side effects as dichotomous outcome

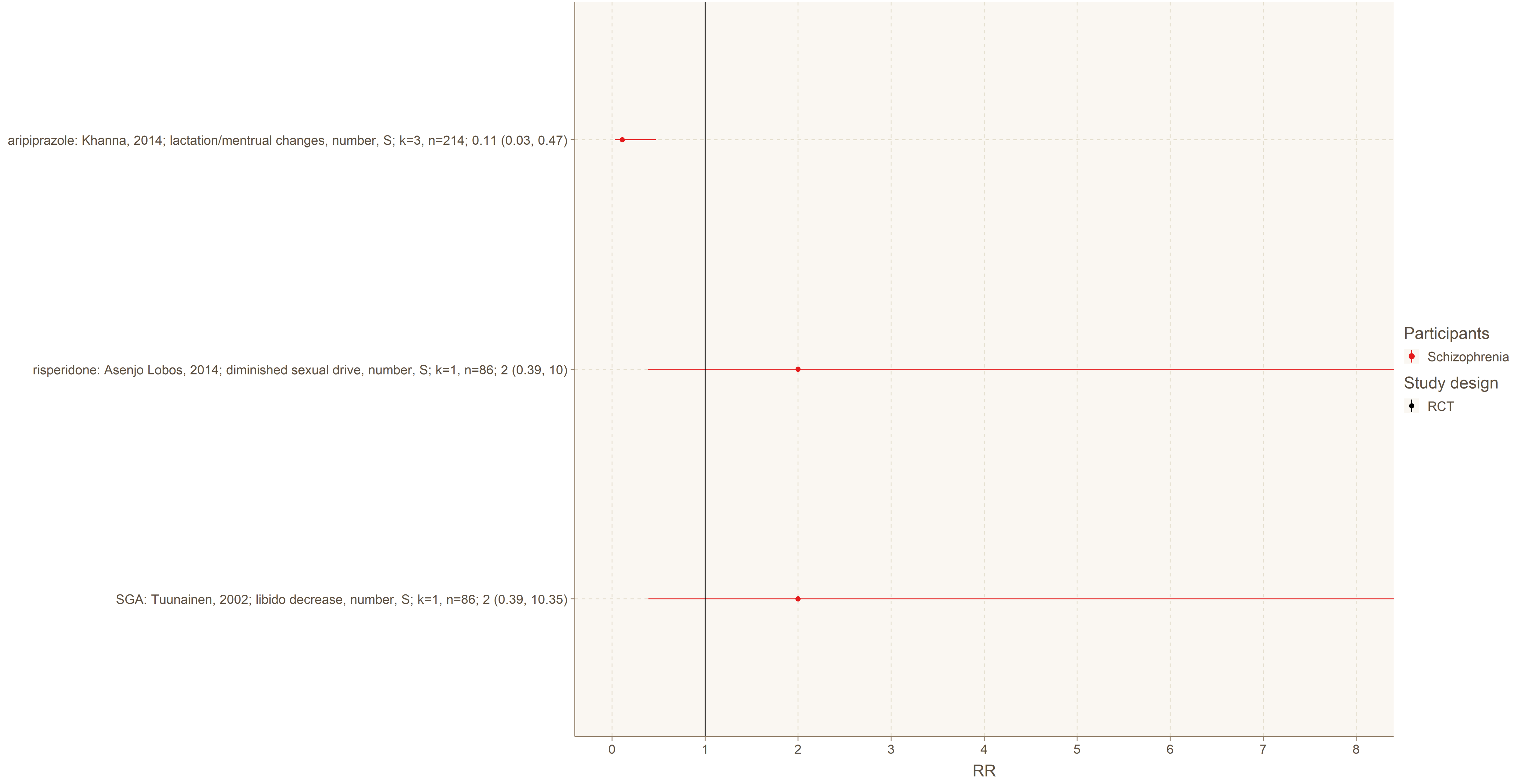

S 15 Glucose abnormalities as dichotomous outcome

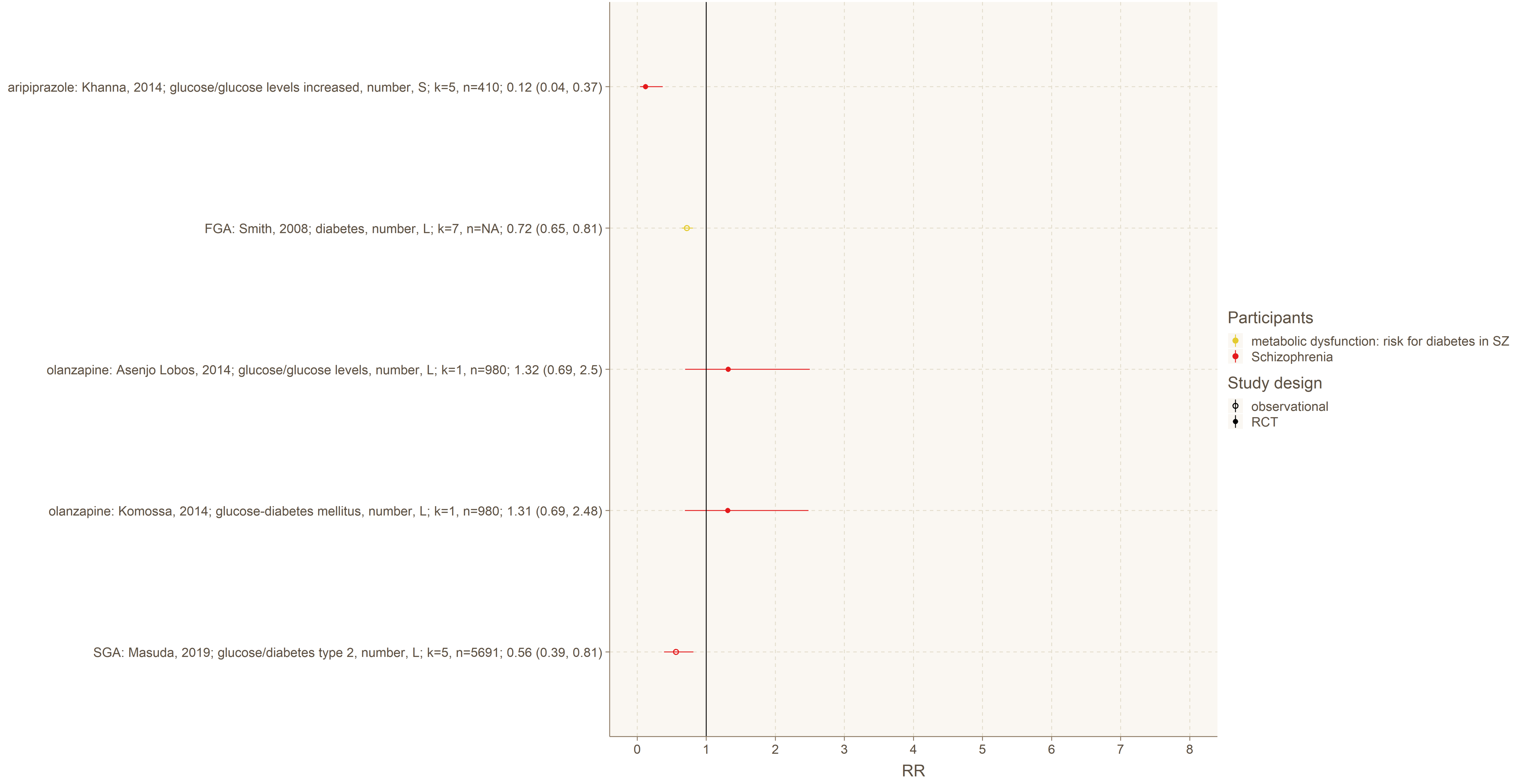

S 16 Hospitalization as dichotomous outcome

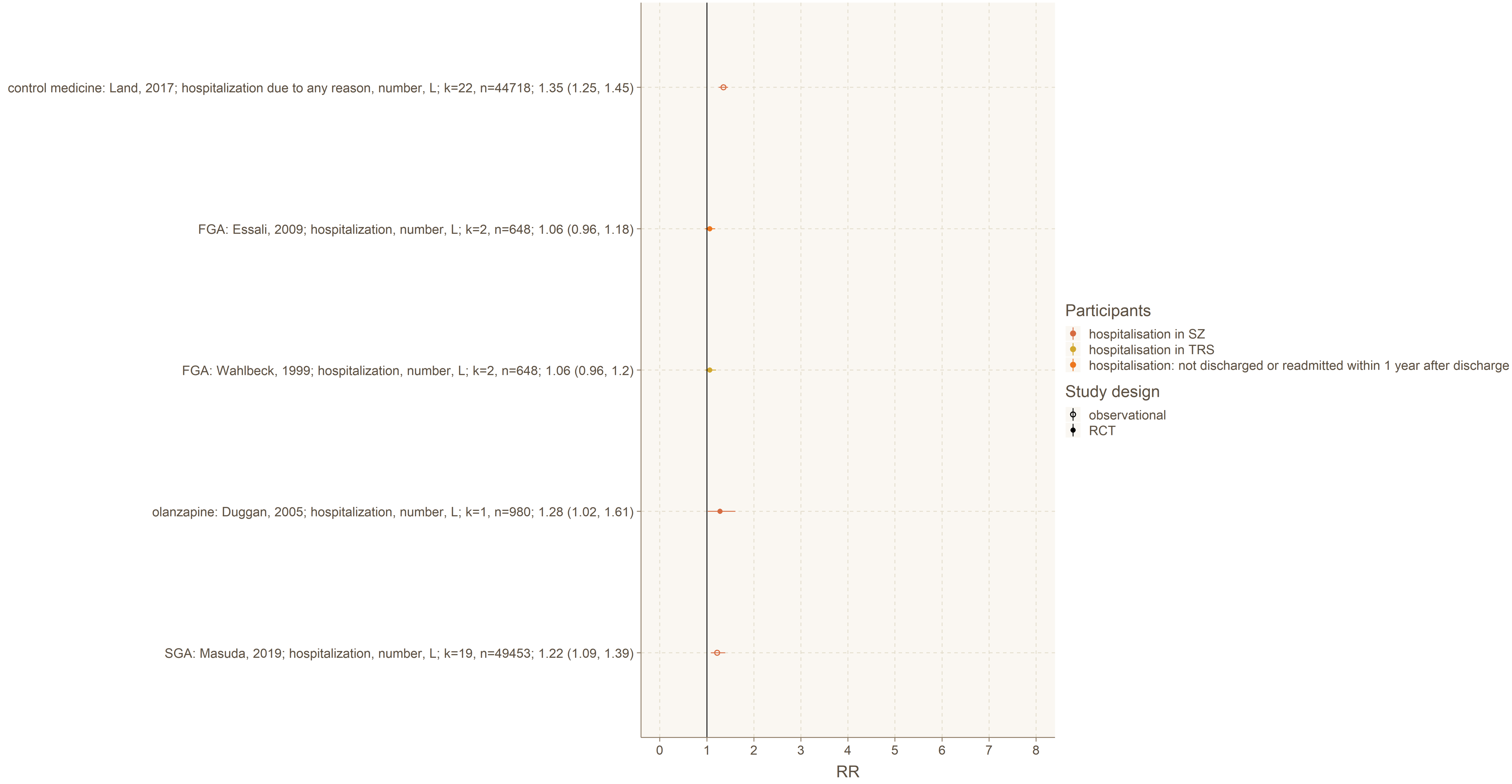

S 17 Suicidality as dichotomous outcome

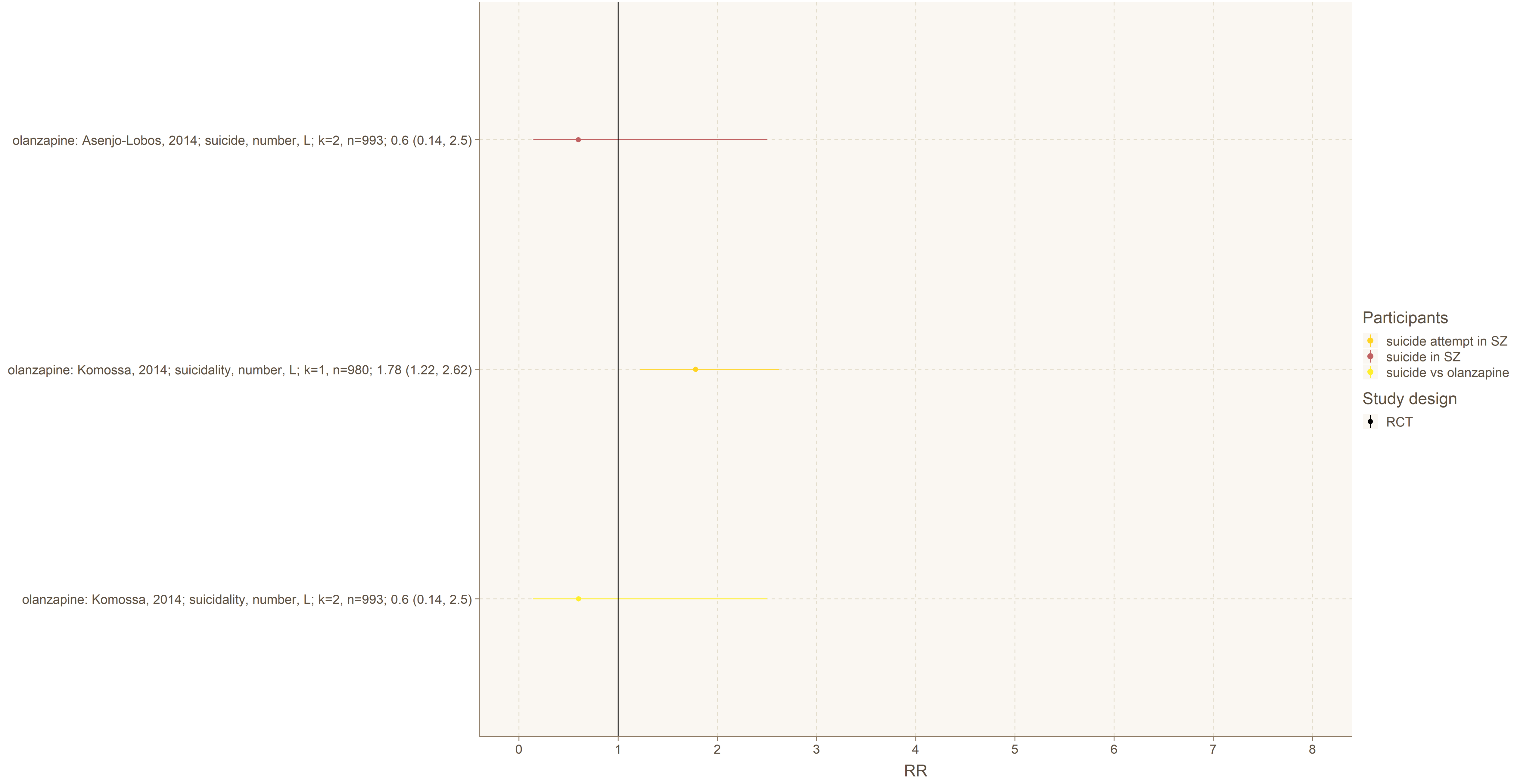

S 18 Efficacy of treatment for hypersalivation as continuous outcome

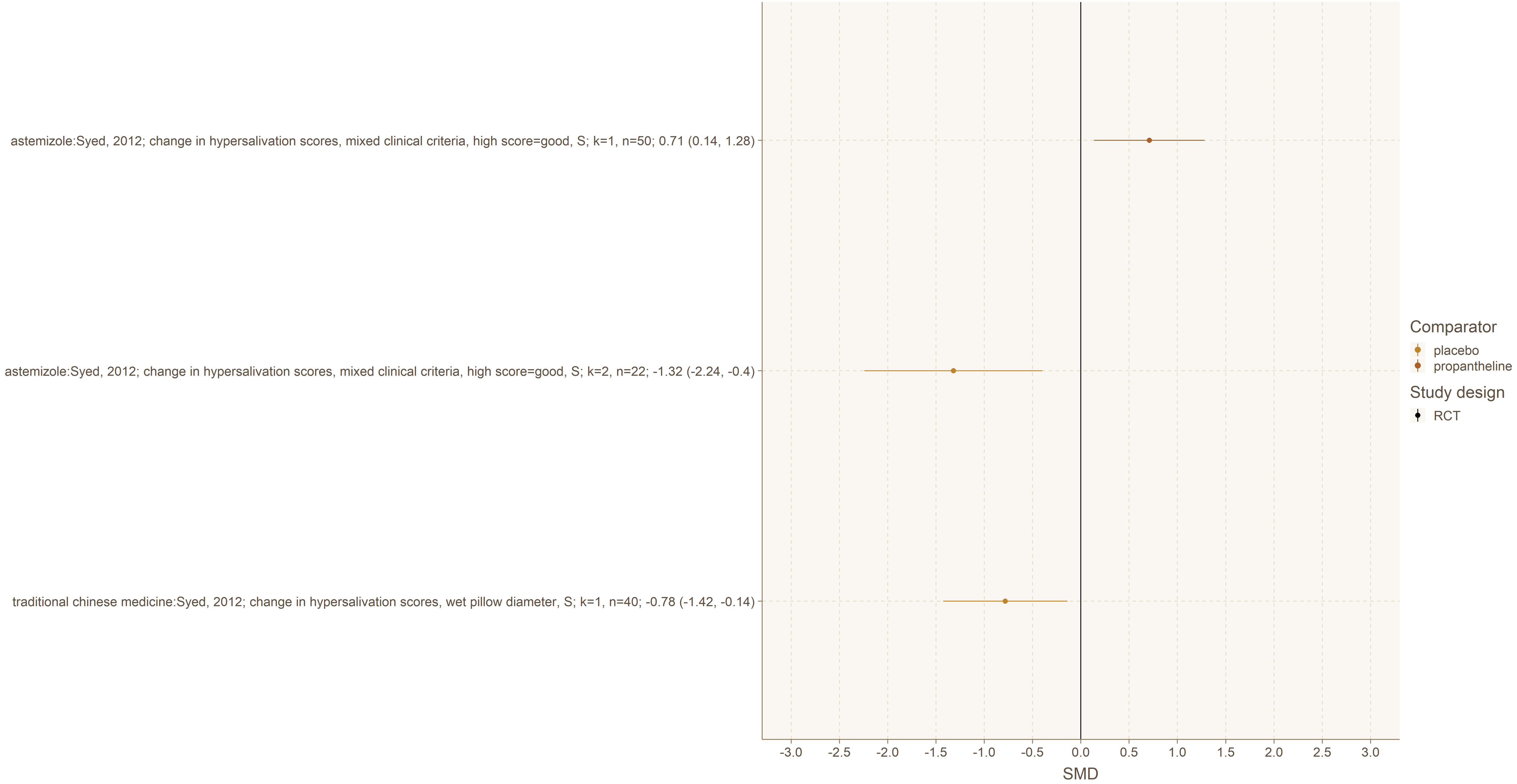

S 19 Efficacy of treatment for constipation as dichotomous outcome

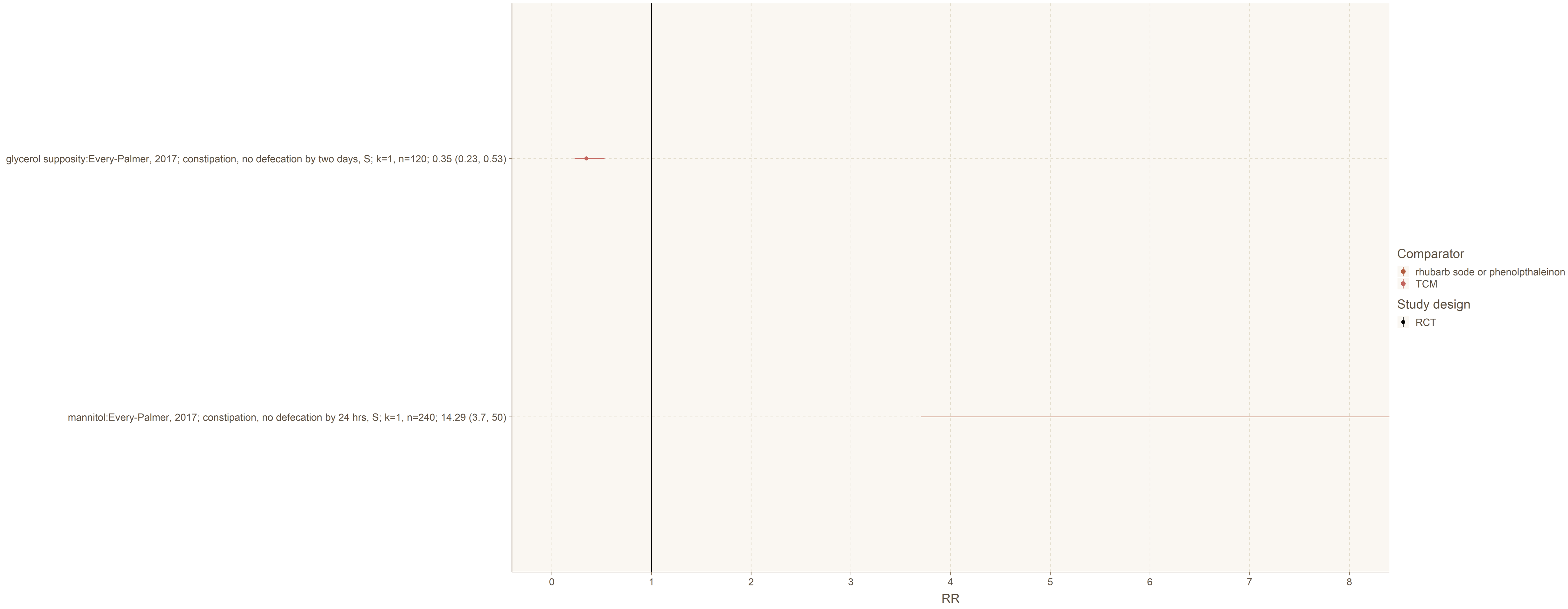

Supplement: Supplementary file 4 — Supplementary Figures S1-S19 [file 41398_2021_1613_MOESM4_ESM.pdf]
